# Bambus[4,6]urils as Dual Scaffolds for Multivalent Iminosugar Presentation and Ion Transport: Access to Unprecedented Glycosidase-Directed Anion Caging Agents

**DOI:** 10.3390/molecules27154772

**Published:** 2022-07-26

**Authors:** Marine Lafosse, Yan Liang, Jérémy P. Schneider, Elise Cartier, Anne Bodlenner, Philippe Compain, Marie-Pierre Heck

**Affiliations:** 1 Département Médicaments et Technologies pour la Santé (DMTS), Université Paris-Saclay, CEA, INRAE, SCBM, 91191 Gif-sur-Yvette, France; marine.lafosse@orange.fr (M.L.); elise.cartier@cea.fr (E.C.); 2 Equipe de Synthèse Organique et Molécules Bioactives (SYBIO), Laboratoire d’Innovation Moléculaire et Applications (LIMA), University of Strasbourg|University of Haute-Alsace|CNRS (UMR 7042), 67087 Strasbourg, France; yolandaly168@outlook.com (Y.L.); jeremy.schneider@live.fr (J.P.S.)

**Keywords:** bambusuril, multivalency, iminosugars, CuAAC, enzyme inhibition, DNJ, α-mannosidase

## Abstract

Bambusurils, BU[4] and BU[6], were used for the first time as multivalent scaffolds to link glycosidases inhibitors derived from 1-deoxynojirimycin (DNJ). Two linear DNJ ligands having six or nine carbon alkyl azido linkers or a trivalent DNJ dendron were grafted onto octapropargylated BU[4] and dodecapropargylated BU[6] using copper-catalyzed cycloaddition (CuAAC) to yield corresponding neoglycobambus[4] and neoglycobambus[6]urils bearing 8 to 24 iminosugars. The inhibition potencies of neoglycoBU[4], neoglycoBU[6] and neoglycoBU[6] caging anions were evaluated against Jack Bean α-mannosidase and compared to monovalent DNJ derivatives. Strong affinity enhancements per inhibitory head were obtained for the clusters holding trivalent dendrons with inhibitory constants in the nanomolar range (*K_i_* = 24 nM for BU[4] with 24 DNJ units). Interestingly, the anion (bromide or iodide) encapsulated inside the cavity of BU[6] does not modify the inhibition potency of neoglycoBU[6], opening the way to water-soluble glycosidase-directed anion caging agents that may find applications in important fields such as bio(in)organic chemistry or oncology.

## 1. Introduction

Bambus[*n*]urils, R_2*n*_BU[*n*] (*n* = 4, 6), are synthetic neutral cyclooligomers composed of R_2_ disubstituted glycoluril units connected by *n*-methylene bridges [1]. BU[4,6] show a rigid conformation in an alternate arrangement that looks similar to a double-cup jigger. The well-defined cavity of BU[6] is of ideal size to entrap anions [1,2,3]. This strong anion-caging property of BU[6] was utilized for anion sensing, [4,5] transport [6,7] and extraction [8,9,10] as well as in photoinduced electron transfer [11]. However, BU[4], having a smaller internal cavity than BU[6] derivatives, is unable to include any anions and so far does not have any applications. Nevertheless, the modular valency of 8 to 12 for R_8_BU[4] and R_12_BU[6], respectively, their topology allowing ligands to be grafted on upper and lower rims, their arm flexibility and, even more importantly, their cheap and easy synthesis make BU[4] and BU[6] attractive scaffolds for the construction of multivalent derivatives. We have previously synthesized allylated bambus[4,6]urils that were submitted to thiol–ene click coupling (TEC) with thiosugars to generate the corresponding 8-12 thiosugar-functionalized bambus[4,6]urils [12,13]. As a proof of concept, we recently developed an alternative strategy using propargylated bambus[4,6]urils able to be post-functionalized by click chemistry (CuAAC) so as to afford multivalent architectures bearing 8 or 12 glucose units [14].

In the present work, we report the first examples of multivalent bambus[*n*]urils–iminosugar conjugates. *N*-alkyl analogs of 1-deoxynojirimycin (DNJ) were chosen as inhitopes (inhibiting epitopes) since the best inhibitory multivalent effects were reported with these motifs on Jack Bean α-mannosidase [15,16,17,18,19,20]. Despite the large number of scaffolds used for multimerization of iminosugar inhitopes [15,16,17,18,19,20,21,22,23,24,25,26], starting from C_60_, polyols, cyclodextrins, porphyrins, calixarenes and gold nanoparticles to the most effective cyclopeptoids, it is still relevant to study new structures that might have different topologies and contact points with glycosidases together with other physical properties of interest, such as anion binding ability. Indeed, it was shown that the binding modes of Jack Bean α-mannosidases with iminosugar-based multivalent inhibitors not only depend on the nature and number of bioactive inhitopes, but more importantly on the shape and size of the multivalent scaffold as well as ligand density [15,16,17,18,19,20]. Multimerization of inhitopes proved to be an attractive strategy, enabling valency-corrected inhibition enhancements of up to three [26] to four [27] orders of magnitude, inhibition selectivity refining or enzyme activation [28,29]. Multivalent drugs on nanoparticles also proved efficient for escaping efflux pumps [30]. Originally discovered with glycosidases [15,16,17,18,19,20], the inhibitory multivalent effect has been generalized to other enzymes such as glycosyltransferases and carbonic anhydrases [31,32,33,34]. In the case of enzyme inhibition, multivalent inhibitors may interact with enzymes by numerous mechanisms, such as the bind-and-recapture process, the chelate effect or receptor clustering [15,16,17,18,19,20,35].

Since anions such as bromide and iodide can easily and strongly be complexed inside BU[6], we have explored whether they would impact the rigidity and shape of the scaffold, and thus possibly the interaction with the model glycosidase used for studying inhibitory multivalent effects. Our objective was to access unprecedented water-soluble glycosidase-directed anion caging agents. Here, we describe the full details of our study, from the synthesis of bambus[*n*]urils–iminosugar conjugates to the quantification of host–guest interaction of these neoglycoclusters with iodide anion and their evaluation as Jack Bean α-mannosidase inhibitors.

## 2. Results and Discussion

### 2.1. Preparation of DNJ-Functionalized Bambusuril neoglyco_8_BU[4], neoglyco_12_BU[6] and X@neoglyco_12_BU[6]

**Figure 1:**
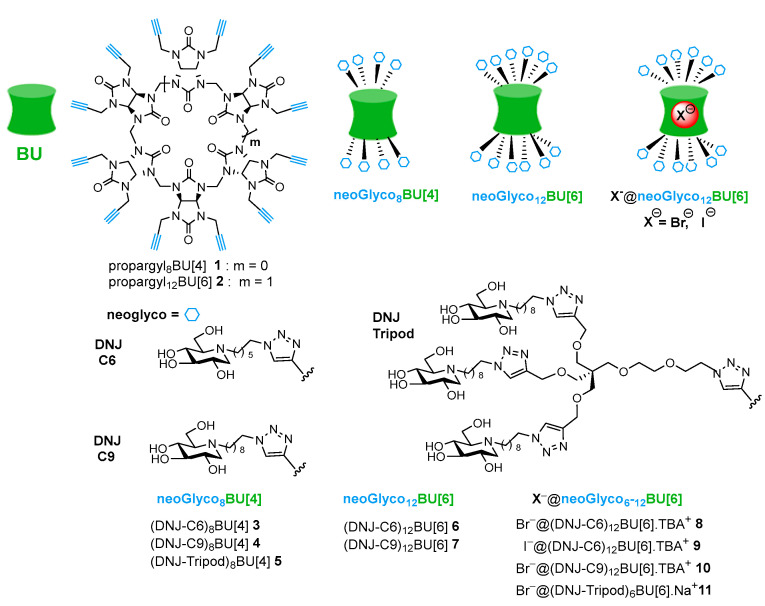
Structures of propargyl_8_BU[4] **1**, propargyl_12_BU[6] **2**, neoglycobambusurils BU[4] **3**–**5** and BU[6] **6**, **7** and Br^−^@BU[6].TBA^+^ **8**, **10**–**11** and I^-^@BU[6].TBA^+^ **9** bearing DNJ ligands studied in this paper.

**Scheme 1:**
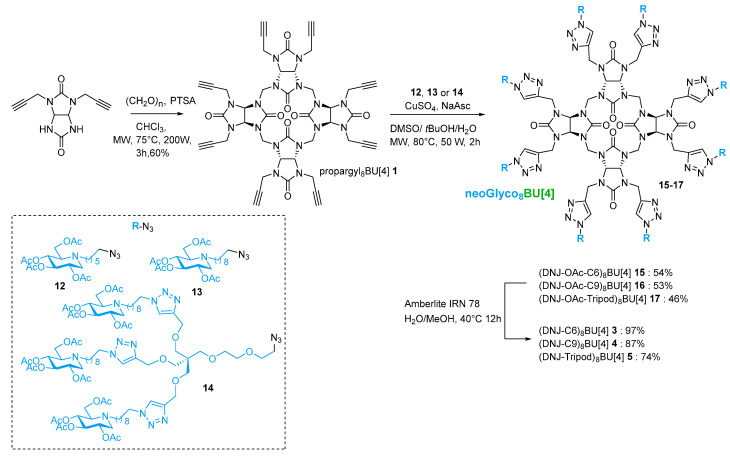
Synthesis of neoglycobambus[4]urils BU[4] **3**, **4** and**5** decorated with C-6, C-9 monovalent DNJ and C-9 trivalent tripod DNJ, respectively.

The best multivalent effects in glycosidase inhibition using non-polymeric inhibitors were obtained thanks to a study relying on a gradual increase in cyclopeptoid scaffold size and valency, which led to small aggregates between two enzymes and the multimeric inhibitor being highlighted as the best cluster of the series [26,36]. Important click partners of this study were azide-armed N-C6 and N-C9 alkyl DNJ [37] and tripod DNJ derivatives [38]. These building blocks were consequently selected for the synthesis of the first examples of bambus[*n*]urils-based iminosugars **3**–**11** (Figure 1). Based on our recent results [14], octapropargylated BU[4] **1** and dodecapropargylated BU[6] **2** were used as clickable platforms to generate architectures decorated with 8 and 12 DNJ inhitopes, respectively. For this purpose, azide-armed *N*-hexyl DNJ **12**, *N*-nonyl DNJ **13** and *N*-nonyl-trivalent DNJ-dendron (tripod) **14** were chosen as peripheral ligands to generate corresponding neoglyco_8_BU[4] **3–5** and neogluco_12_BU[6] **6**–**11** derivatives. The syntheses of DNJ-functionalized bambusuril BU[4] **3**–**5** and the structures of azide DNJ ligands **12–14** are shown in Scheme 1.

First, the dipropargylated BU[4] platform **1** was prepared in one step and with 60% yield by condensation of propargylglycoluril and formaldehyde following our reported procedure [14]. Propargyl_8_BU[4] **1** was then reacted with peracetylated azido-functionalized C6-DNJ **12** [37,39] through a copper(I)-catalyzed azide alkyne cycloaddition reaction (CuAAC) in the presence of sodium ascorbate in DMSO/*t*BuOH/H_2_O to acquire protected (DNJ-OAc-C6)_8_BU[4] **15** with 54% yield. Use of microwave irradiations shortened the reaction time and increased yield. Subsequently, deacetylation of **15** was set up using anion exchange (OH^−^) amberlite IRN 78 resin as described [37,40] to generate (DNJ-C6)_8_BU[4] **3** with 97% yield. Following the same synthetic procedure, (DNJ-OAc-C9)_8_BU[4] **16** was isolated with 53% yield from the CuAAC reaction of propargyl_8_BU[4] **1** and peracetylated azido-functionalized C9-DNJ **13** [37]. Basic cleavage of OAc groups of **16** afforded (DNJ-C9)_8_BU[4] **4** decorated with 8 DNJ (C-9 linker) with 87% yield. Grafting of 24 DNJ derivatives was made possible by the click coupling of protected azido trivalent-C9-DNJ dendron **14** [38,41] on BU[4] **1** to give (DNJ-OAc-Tripod)_8_BU[4] **17** with 46% yield. Basic treatment of **17** generated (DNJ-Tripod)_8_BU[4] **5** with 74% yield.

**Scheme 2:**
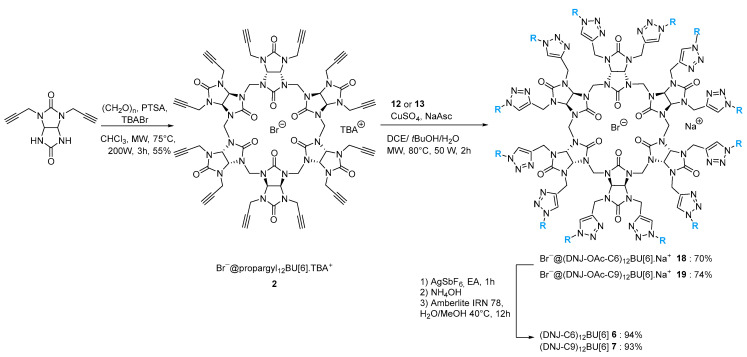
Synthesis of neoglyco_12_BU[6] **6**, **7** bearing 12 (C-6, C-9) monovalent DNJ ligands.

A larger platform, Br^−^@propargyl_12_BU[6].TBA^+^ **2**, that was able to be decorated with 12 iminosugar inhitopes was then submitted to azide-armed DNJ derivatives **12** and **13** to generate, after decomplexation and deprotection steps, the corresponding 12-valent clusters (DNJ-C6)_12_BU[6] **6** and (DNJ-C9)_12_BU[6] **7**, respectively (Scheme 2).

First, following our reported procedure, condensation of dipropargyl glycoluril with formaldehyde and TBABr as template promoted the formation of Br^−^@propargyl_12_BU[6]BU.TBA^+^ **2** with 55% yield [14]. A subsequent CuAAC reaction of **2** with azide-armed DNJ **12** (C6 linker) afforded fully functionalized Br^-^@(DNJ-C6-OAc)_12_BU[6].Na^+^ **18** with 70% yield. Click reaction of BU **2** with azide-armed DNJ **13** (C9 linker) provided 12-valent Br^−^@(DNJ-C9-OAc)_12_BU[6].Na^+^ **19** with 74% yield. Decomplexation reactions of Br^-^@BU **18** and **19** were then performed with AgSbF_6_ [13] to generate the corresponding anion-free 12-valent iminosugars that were directly submitted to basic resin for the final deprotection step, yielding (DNJ-C6)_12_BU[6] **6** (94% yield) and (DNJ-C9)_12_BU[6] **7** (93% yield), respectively.

**Scheme 3:**
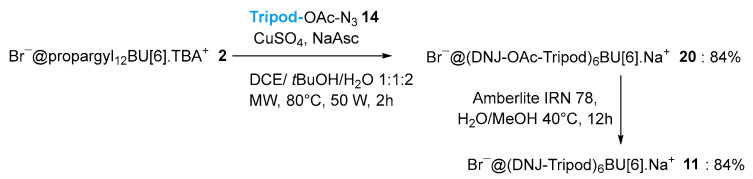
Synthesis of Br^−^@(DNJ-Tripod)_6_BU[6].Na^+^ **11**.

To obtain the 12 × 3-valent DNJ-BU[6] conjugate, Br^-^@propargylBU[6].TBA^+^ **2** was reacted with clickable dendron **14** [38] in the presence of CuSO_4_ and sodium ascorbate using microwave irradiations to promote full click coupling (Scheme 3). However, using our general click procedure, only partial functionalization of **2** was observed, affording 6 × 3 valent-DNJ-Tripod-BU[6] **20** with 84% yield. Despite many attempts to optimize the reaction conditions (solvent, stoichiometry, microwave power, temperature, reaction time), it was not possible to obtain fully functionalized BU[6]. We hypothesized that the steric hindrance of the DNJ Tripod **14** did not allow the total functionalization of BU[6] **2**. Mass spectra analysis allowed for quantification of the presence of six grafted tripods. The six remaining alkyne functions were observable on the ^1^H NMR spectrum of **20**. Despite its partial functionalization, Br^−^@(DNJ-Tripod-OAc)_6_ BU[6].Na^+^ **20** was submitted to basic resin deprotection to give Br^−^@(DNJ-Tripod)_6_ BU[6].Na^+^ **11** with 84% yield. This interesting cluster bearing 18 DNJ units has been also evaluated as a glycosidase inhibitor.

**Scheme 4:**
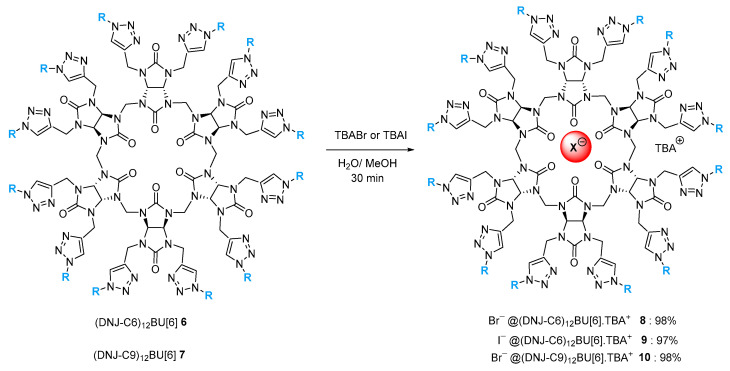
Synthesis of Br^-^@(DNJ-C6)_12_BU[6].TBA^+^ **8**, I^−^@(DNJ-C6)_12_BU[6].TBA^+^ **9** and Br^−^@(DNJ-C9)_12_BU[6] TBA^+^ **10**.

Interestingly, bambusurils BU[6] are known to complex halides, especially iodides, with very high affinity forming a rigid assemblage [1,42]. We hypothesized that such a rigid structure could influence the glycosidase inhibition power of iminosugar–BU conjugates. Towards this goal, TBABr was added to anion-free (DNJ-C6-OH)_12_BU[6] **6** in H_2_O/MeOH to generate Br^-^@(DNJ-C6-OH)_12_BU[6].TBA^+^ **8** (98% yield). Similarly, addition of TBAI to (DNJ-C6-OH)_12_BU[6] **6** yielded I^-^@(DNJ-C9-OH)_12_BU[6].TBA^+^ **9** (97% yield) and reaction of anion-free (DNJ-C9-OH)_12_BU[6] **7** with TBABr gave Br^−^@(DNJ-C9-OH)_12_BU[6].TBA^+^ **10** (98% yield). The synthesis of anion@BU[6] **8**–**10** is reported in Scheme 4.

The hostguest interaction of anion-free (DNJ-C6)_12_BU[6] **6** with iodide anion was evaluated using a direct isothermal titration calorimetry (ITC) method [4,14]. BU[6] **6** bearing 12 DNJ arms (C6 linker) was found to have a good affinity for iodide (*K_a_* = 4.8 × 10^5^ M^−1^ in water) (Supplementary Materials Figure S1), similar to our reported results for (D-glucose)_12_BU[6] derivatives (*K_a_* = 2.2 × 10^5^ and 1.7 × 10^5^ M^−1^ for iodide complexation in H_2_O) [14]. Interestingly, this study shows that C6 linkers, or the nature of the peripheral head (DNJ compared to D-glucose), did not affect complexation. In addition, ITC data clearly indicate that the formation of the iodide complex **9** is driven by enthalpy (ΔH = −33, 47 kJ/mol) and that **9** displays a 1:1 stoichiometry in water.

### 2.2. Glycosidase Inhibition Study

**Figure 2:**
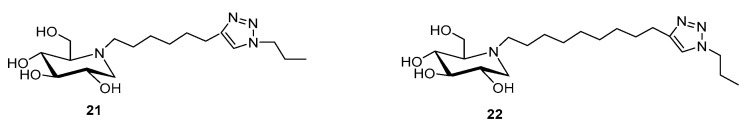
Structure of monovalent inhibitors **21**–**22**.

All the new clusters were evaluated against Jack Bean α-mannosidase, a glycosidase highly sensitive to multivalent inhibitor presentation [15,16,17,18,19,20]. The corresponding inhibition constants are summarized in Table 1. The evaluated DNJ-bambusuril conjugates acted as competitive inhibitors, with the notable exception of the series of 12-valent clusters with the shorter C6 linker obtained with the BU[6] scaffold which behaved as mixed inhibitors (Table 1, entries 6–8). The inhibition potency of those new clusters was compared to their corresponding monovalent inhibitors, **21** [22] and **22** [37] (Figure 2), to calculate their relative potency (*rp*). Dividing the relative potency by the number of active units gives the relative potency per inhitope (*rp/n*).

As in a previous study with calix[8]arene-based clusters [43], the scaffold has a strong impact on inhibition with the C6 shorter linker. Whereas the inhibitory heads of cluster **3** based on the smaller BU[4] scaffold are individually four times more potent than the reference (*rp/n* = 4), the ones of clusters **6**, **8** and **9** based on BU[6] are less active than the monovalent reference (*rp/n* < 1) and show a different inhibition mode (Table 1 entries 6-8). Noticeably, the inhibition mode and power of the three BU[6] binding bromide or iodide ions (or with a free cavity) are similar, suggesting an overall similar shape. We previously obtained crystals of Cl^−^, Br^−^ I^−^ @allyl_12_BU[6].TBA^+^ [12,13] and of Br^−^, I^−^ @propargyl_12_BU[6].TBA^+^ [14], showing that the halides are included in the cavity and that the geometry of these CH···halide bonds are similar, with distances varying with the halogen ionic radius which thus indicates that the macrocycle retains some flexibility [12,13,14]. Similarly, with C9 linkers, the activities and inhibition mode are not impacted by the presence of anions in the bambusuril cavity (Table 1 entries 9-10). With the longer C9 linker, the impact of the central scaffold, whatever its size, is abolished, giving similar affinity enhancement per DNJ (entries 4, 9 and 10) with C9 enhancement being at least one order of magnitude higher than for the C6 series. This result is in line with previous findings using other scaffolds [15,16,17,18,19,20,43]. The best results were obtained with clusters **5** and **11** grafted with the trivalent dendron with affinity enhancements one order of magnitude higher than those of compounds **4**, **7** and **10**. The affinity enhancement per inhibitory head of compound **5** is in the same range as for the 24-valent cyclopeptoid bearing the same trivalent dendron [26]. Altogether, those results show that bambusuril scaffolds allow for efficient grafting of glycosidase inhibitors and that this multimerization induces strong affinity enhancements. Moreover, the fact that activity is not influenced by anion binding opens the way to potential therapeutic applications by inducing accumulation of the encaged anion, and potentially radioactive iodide, in a glycosidase rich environment. It is, for example, well-known that β-glucuronidases are present at high concentration in the microenvironment of most solid tumours [44] and that radioactive iodine is used for the treatment of cancers, including thyroid cancers [45,46,47].

## 3. Material and Methods

### 3.1. General Information and General Experimental Procedures for the Syntheses

Commercially available reagents were used without further purification. All reactions were performed under inert atmosphere using anhydrous solvents which were dried and distilled before being used. Thin-layer chromatograms (TLC) and flash chromatography separations were respectively performed on precoated silica gel 60 F254 plates (0.25 mm) and on Merck Kieselgel 60 (grading 40–63 μm). Microwave syntheses were conducted using a CEM Focused Microwave Discover^®^ SP-X System reactor. The reactions were performed for 2–4 h under magnetic stirring in 10 or 35 mL sealed Discovered SP vessels closed with Activent® caps. The dynamic control method was used for all microwave reactions where the temperature and the pressure were set (P = 50 W, T = 80 °C, PowerMax on). ^1^H NMR spectra (400 MHz) and ^13^C NMR spectra (100 MHz) were recorded on a Brucker Avance 400 MHz spectrometer. Chemical shifts and coupling constants are reported in parts per million (ppm) and in Hertz (Hz), respectively. HRMS and electrospray mass spectra (ESIMS) were obtained from an LCT Premier XE using electro spray ionization coupled with a time flight analyzer (ESI-TOF). Infrared spectra (IR) were recorded on a Perkin Elmer UAR Two Spectrum spectrometer. Electrospray mass spectra were obtained using an ESI-Quadripole autopurify Waters (pump: 2545, mass: ZQ2000) mass Spectrometer. Optical rotations were measured on a JASCO P-2000 Polarimeter. Melting points were measured on a Büchi Melting point B540.

#### 3.1.1. General Procedure A for CuAAC Reaction of Propargyl_8_BU[4]

To a solution of octapropargyl bambus[4]uril **1** (10 to 15 mg, 1 eq.) and azide ligand (9 eq.) in DMF/H_2_O (0.6 mL, 5/1) in a microwave reactor, a solution of sodium ascorbate 1 M in water (0.4 eq.) and a solution of CuSO_4_ 5H_2_O 1M in H_2_O (0.8 eq.) were added. The reaction was subjected to microwave irradiations under magnetic stirring from 30 min to 4 h at 80 °C. A solution of CH_3_CN/H_2_O/30w% NH_4_OH (16 mL, 15/0.5/0.5, *v*/*v*/*v*) was then added to the crude and the mixture was filtered on a small pad of SiO_2_ and eluted with a solution of CH_3_CN/H_2_O/30w%-NH_4_OH (15:0.5:0.5, *v*/*v*/*v*). The filtrate was concentrated under vacuum and purified by flash silica gel chromatography (SiO_2_; CH_2_Cl_2_/MeOH, 99:1 to 95:5) to afford the corresponding 8-clicked iminosugars BU[4].

#### 3.1.2. General Procedure B for CuAAC Reaction of Propargyl_12_BU[6]

To a solution of dodecapropargybambus[6]uril *tetra*-butyl bromide **2** (10 to 15 mg, 1 eq.) and azide ligand (16 eq.) in DCE/H_2_O/*t*BuOH (0.5/1/1.2 mL) in a microwave reactor, a solution of sodium ascorbate 1 M in water (4 eq.) and a solution of CuSO_4_.5H_2_O 1M in H_2_O (2 eq.) were added. The reaction was subjected to microwave irradiations under magnetic stirring for 2 h at 80 °C. The mixture was then concentrated under vacuum and diluted with CH_3_CN/H_2_O/30w% NH_4_OH (9/1/1, 11 mL). The mixture was filteredon a small pad of SiO_2_ (typically 1 cm thick) that was washed with this same eluent, CH_3_CN/H_2_0/30w%-NH_4_OH (9/1/1, 25 mL). The filtrate was concentrated under vacuum and purified by flash silica gel chromatography (SiO_2_; CH_2_Cl_2_/MeOH, 99:1 to 90) to afford the corresponding 12-clicked iminosugars BU[6].

#### 3.1.3. General Procedure C for Deacetylation of Sugars

Amberlite IRN 78 (HO^−^) (n g/mmol with n = number of OAc) was added to a solution of acetylated-iminosugars bambusuril (1 eq.) in H_2_O/MeOH (1/1, 600 μL). The suspension was stirred from 4 h to 12 h at 40 °C. The resin was removed by filtration and washed with methanol and water. The filtrate was concentrated under vacuum to give BU-iminosugars.

#### 3.1.4. Synthesis (DNJ-OAc-C6)_8_BU[4] **15**

Following general procedure A and starting with propargyl_8_bambus[4]uril **1** (10.1 mg, 10.9 µmol, 1 eq.) and azidoiminosugar **12** (60 mg, 132 µmol, 12 eq.), (DNJ-OAc-C6)_8_BU[4] **15** (27.1 mg, 54% yield) was obtained as a colorless oil. ^1^H NMR (300 MHz, CDCl_3_): δ (ppm) 7.56 (s, 8H, H13), 5.74 (s, 6H, H17), 5.10–4.99 (m, 16H, H3, H4), 4.99–4.89 (m, 8H, H2), 4.66–4.44 (m, 24H, H15, H19), 4.42–4.21 (m, 16H, H12), 4.20–4.04 (m, 16H, H6), 3.17 (dd, *J* = 11.0, 5.0 Hz, 8H, H1a), 2.78–2.66 (m, 8H, H7a), 2.66–2.58 (m, 8H, H5), 2.58–2.47 (m, 8H, H7b), 2.29 (dd, *J* = 11.0, 10.5 Hz, 8H, H1b), 2.10–1.96 (singlets, 96H, CH_3_-C=O), 1.93–1.81 (m, 16H, H11), 1.53–1.08 (m, 48H, H8, H9, H10); ^13^C NMR (75 MHz, CDCl_3_): δ (ppm) 170.9, 170.4, 170.1, 169.8 (CH_3_-C=O), 159.7, 158.3 (C16, C18), 143.8 (C14), 122.8 (C13), 74.8 (C3), 71.6 (C17), 69.6 (C4), 69.5 (C2), 61.7 (C5), 59.7 (C6), 53.0 (C1), 51.7 (C7), 50.4 (C12), 39.0 (C15), 30.3 (C11), 26.8, 26.6 (C9, C10), 25.0 (C8), 21.0, 20.85, 20.8 (CH_3_-C=O), C-9 could not be observed with 1024 scans; IR (neat, ν_max_/cm^−^^1^) 1744 (CH_3_-C=O), 1710 (NC=O); HRMS (ESI+): *m*/*z* calcd for C_204_H_297_N_48_Na_2_O_72_ [M + H + 2Na]^3+^: 1539.0278, found 1539.0257; [α]_D_^20^ = +2.2 (c 1, CH_3_OH).

#### 3.1.5. Synthesis of (DNJ-OAc-C9)_8_BU[4] **16**

Following general procedure A and starting with propargyl_8_bambus[4]uril **1** (10 mg, 10.9 µmol, 1 eq.) and azidoiminosugar **13** (65 mg, 130 µmol, 12 eq.), (DNJ-OAc-C9)_8_BU[4] **16** (28.3 mg, 53% yield) was isolated as a colorless oil. ^1^H NMR (400 MHz, CDCl_3_): δ (ppm) 7.55 (br s, 8H, H16), 5.74 (br s, 6H, H20), 5.10–4.99 (m, 16H, H3, H4), 4.95–4.90 (m, 8H, H2), 4.65–4.51 (m, 24H, H18, H22), 4.38–4.23 (m, 16H, H15), 4.19–4.09 (m, 16H, H6), 3.18 (dd, *J* = 11.2, 4.9 Hz, 8H, H1a), 2.76–2.66 (m, 8H, H7a), 2.65–2.60 (m, 8H, H5), 2.59–2.50 (m, 8H, H7b), 2.32 (dd, *J* = 11.2, 10.3 Hz, 8H, H1b), 2.07 (s, 24H, CH_3_-C=O), 2.0–1.97 (singlets, 72H, CH_3_-C=O), 1.89–1.83 (m, 16H, H14), 1.50–1.11 (m, 96H, H8, H9, H10, H11, H12, H13); ^13^C NMR (100 MHz, CDCl_3_): δ (ppm) 171.0, 170.5, 170.1, 169.8 (CH_3_-C=O), 159.7, 158.4 (C-19, C-21), 143.8 (C-17), 122.7 (C-16), 74.8 (C-3), 71.5 (C-20), 69.7 (C-4), 69.6 (C-2), 61.6 (C-5), 59.7 (C-6), 53.0 (C-1), 51.9 (C-7), 50.6 (C-15), 38.9 (C-18), 30.3 (C-14), 29.8, 29.6, 29.1, 27.3, 26.7 (C-9, C-10, C-11, C-12, C-13), 24.8 (C-8), 21.0, 20.97, 20.88, 20.81 (CH_3_-C=O), C22 could not be observed with 1024 scans; IR (neat, ν_max_/cm^−^^1^) 1742 (CH_3_-C=O), 1705 (NC=O); HRMS (ESI): *m*/*z* calcd for C_228_H_347_N_48_O_72_ [M + 3H]^3+^: 1636.4983, found 1636.5006; [α]_D_^20^ = +2.6 (c 1, CH_3_OH).

#### 3.1.6. Synthesis of (DNJ-OAc-Tripod)_8_BU[4] **17**

Following general procedure A and starting with propargyl_8_bambus[4]uril **1** (3 mg, 3.3 µmol, 1 eq.) and azido-tripod **14** (84 mg, 45.6 µmol, 14 eq.), (DNJ-OAc-tripod)_8_BU[4] **16** (23.7 mg, 1.5 μmol, 46% yield) was isolated as a colorless oil. ^1^H NMR (400 MHz, CDCl_3_): δ (ppm) 7.69–7.67 (br s, 8H, H26), 7.65–7.52 (br s, 24H, H16), 5.76 (br s, 6H, H30), 5.11–4.99 (m, 48H, H3, H4), 4.99–4.90 (m, 24H, H2), 4.60–4.44 (m, 88H, H18, H25, H28, H32), 4.31 (t, *J* = 7.4Hz, 48H, H15), 4.17–4.11 (br s, 48H, H6), 3.88–3.75 (m, 16H, H24), 3.62–3.35 (m, 96H, H19, H21, H22, H23), 3.18 (dd, *J* = 11.2, 5.1 Hz, 24H, H1a), 2.76–2.66 (m, 24H, H7a), 2.66–2.60 (m, 24H, H5), 2.60–2.48 (m, 24H, H7b), 2.32 (dd, *J* = 11.2, 10.5 Hz, 24H, H1b), 2.07–1.98 (several singlets, 288H, CH_3_-C=O), 1.93–1.82 (m, 48H, H14), 1.48–1.12 (m, 288H, H8, H9, H10, H11, H12, H13); ^13^C NMR (CDCl_3_, 100 MHz): δ (ppm) 171.0, 170.5, 170.1, 169.9 (CH_3_-C=O), 159.8, 158.4 (C29, C31), 145.3 (C27), 143.7 (C17), 123.9 (C26), 122.7 (C16), 74.8 (C3), 71.0, 70.3, 70.0 (C21, C22, C23), 69.6 (C4, C24), 69.5 (C2), 69.3 (C19), 65.1 (C18), 61.5 (C5), 59.6 (C6), 53.0 (C1), 51.9 (C7), 50.4 (C15, C25), 45.5 (C20), 38.7 (C28), 30.5 (C14), 29.8, 29.6, 29.1, 27.3, 26.7 (C9, C10, C11, C12, C13), 24.7 (C8), 21.01, 20.98, 20.88, 20.8 (CH_3_-C=O); IR (neat, ν_max_/cm^−^^1^) 1743 (strong, (CH_3_-C=O)); HRMS (ESI, deconvoluted): *m*/*z* calcd for C_740_H_1164_N_136_O_240_ [M + 12H]^12+^: 1317.15, found 1317.16; [α]_D_^20^ = +1.4 (c 1, CH_3_OH).

#### 3.1.7. Synthesis of (DNJ-C6)_8_BU[4] **3**

Following general procedure C and starting with (DNJ-OAc-C6-triazol)_8_BU[4] **15** (27.4 mg, 6.0 µmol), corresponding (DNJ-C6-triazol)_8_BU[4] **3** (18.8 mg, 5.8 µmol, 97% yield) was obtained as a colorless oil. ^1^H NMR (400 MHz, D_2_O): δ (ppm) 7.91 (br s, 8H, H13), 5.81 (br s, 6H, H17), 4.70–4.58 (m, 16H, H15), 4.53–4.39 (m, 8H, H19), 4.40–4.22 (m, 16H, H12), 3.87–3.77 (m, 16H, H6), 3.53 (td, *J* = 10.0 and 4.8 Hz, 8H, H2), 3.40–3.34 (m, 8H, H4), 3.26–3.21 (m, 8H, H3), 2.96 (dd, *J* = 10.3, 4.8 Hz, 8H, H1a), 2.75–2.60 (m, 8H, H7a), 2.60–2.48 (m, 8H, H7b), 2.29–2.14 (m, 16H, H1b, H5), 1.87–1.71 (m, 16H, H11), 1.45–1.31 (m, 16H, H8), 1.27–1.10 (m, 32H, H9, H10); ^13^C NMR (100 MHz, D_2_O): δ (ppm) 160.0, 159.3 (H16, H18), 143.1 (H14), 123.9 (H13), 78.4 (H3), 71.3 (H17), 70.0 (H4), 68.8 (H2), 65.1 (H5), 57.5 (H6), 55.4 (H1), 51.9 (H7), 50.3 (H12), 48.5 (C19), 38.3 (C15), 29.4 (C11), 26.2, 25.5 (C9, C10), 22.6 (C8); IR (neat, ν_max_/cm^−^^1^) 1694 (strong, NC=O); HRMS (ESI): *m*/*z* calcd for C_140_H_235_N_48_O_40_ [M + 3H]^3+^: 1076.2605, found 1076.2629; [α]_D_^20^ = −12.3 (c 1, H_2_O + TFA (1 drop)).

#### 3.1.8. Synthesis of (DNJ-C9)_8_BU[4] **4**

Following general procedure C and starting with (DNJ-OAc-C9)_8_BU[4] **16** (23.5 mg, 4.8 µmol), corresponding (DNJ-C9)_8_BU[4] **4** (14.9 mg, 4.2 µmol, 87%) was obtained as a colorless oil. ^1^H NMR (400 MHz, MeOD): δ (ppm) 7.89 (br s, 8H, H16), 5.80 (br s, 6H, H20), 4.66–4.60 (br s, 16H, H18), 4.59–4.53 (br s, 8H, H22), 4.38 (t ap, *J* = 6.9 Hz, 16H, H15), 3.86–3.82 (m, 16H, H6), 3.47 (td, *J* = 9.4, 4.6 Hz, 8H, H2), 3.40–3.35 (m, 8H, H4), 3.13 (dd, *J* = 9.0 Hz, 8H, H3), 2.98 (dd, *J* = 11.7, 5.0 Hz, 8H, H1a), 2.83–2.71 (m, 8H, H7a), 2.62–2.49 (m, 8H, H7b), 2.16 (dd, *J* = 11.1 Hz, 8H, H1b), 2.10 (d, *J* = 9.1 Hz, 8H, H5), 1.93–1.84 (m, 16H, H14), 1.53–1.42 (m, 16H, H8), 1.39–1.21 (m, 80H, H9, H10, H11, H12, H13); ^13^C NMR (125 MHz, MeOD, 2218 scans, cryogenic probe): δ (ppm) 161.3, 160.1 (C19, C21), 144.7 (C17), 124.7 (C16), 80.4 (C3), 72.6 (C20), 71.9 (C4), 70.6 (C2), 67.2 (C5), 59.4 (C6), 57.6 (C1), 53.7 (C7), 51.7 (C22), 51.5 (C15), 39.6 (C18), 31.3 (C14), 30.5, 30.1, 30.0, 28.6, 27.5 (C9, C10, C11, C12, C13), 24.9 (C8); IR (neat, ν_max_/cm^−^^1^) 3343 (strong broad, OH), 1696 (strong, NC=O); HRMS (ESI, deconvoluted): *m*/*z* calcd for C_164_H_287_N_48_O_40_ [M + 7H]^7+^: 509.8837, found 509.8893; *m*/*z* calcd for C_164_H_286_N_48_O_40_ [M + 6H]^6+^: 594.6964, found 594.6979; *m*/*z* calcd for C_164_H_285_N_48_O_40_ [M + 5H]^5+^: 713.4341, found 713.4314; [α]_D_^20^ = −7.1 (c 1, CH_3_OH/H_2_O 94/6).

#### 3.1.9. Synthesis of (DNJ-Tripod)_8_BU[4] **5**

Following general procedure C and starting with (DNJ-OAc-tripod)_8_BU[4] **17** (23.5 mg, 1.5 µmol), corresponding (DNJ-tripod)_8_BU[4] **7** (12.9 mg, 1.1 µmol, 74% yield) was obtained as a colorless oil. ^1^H NMR (400 MHz, MeOD): δ (ppm) 8.03–7.92 (br s, 32H, H16, H26), 5.88–5.72 (br s, 6H, H30), 4.60–4.44 (m, 80H, H18, H25, H28), 4.42–4.27 (m, 56H, H15, H32), 3.93–3.76 (m, 64H, H6, H24), 3.56–3.33 (m, 144H, H2, H4, H19, H21, H22, H23), 3.19 (t, *J* = 9.2 Hz, 24H, H3), 2.97 (dd, *J* = 11.1, 4.9 Hz, 24H, H1a), 2.78–2.66 (m, 24H, H7a), 2.64–2.52 (m, 24H, H7b), 2.21 (t, *J* = 10.8 Hz, 24H, H1b), 2.15 (dt, *J* = 9.4, 2.6 Hz, 24H, H5), 1.88–1.76 (m, 48H, H14), 1.49–1.37 (m, 48H, H8), 1.31–1.15 (m, 240H, H9, H10, H11, H12, H13); ^13^C NMR (125 MHz, MeOD, 12288 scans): δ (ppm) 170.9, 170.0 (C29, C31), 146.1 (C17, C27), 125.2 (C16, C26), 80.4 (C3), 71.9 (C4), 70.6 (C2), 73.1, 72.5, 72.1, 71.3, 70.4, 70.1, 69.5 (C19, C21, C22, C23, C24, C30), 67.1 (C5), 65.4 (C18), 59.5 (C6), 57.5 (C1), 53.8 (C7), 51.5 (C15, C25), 50.7 (C32), 46.5 (C20), 40.1 (C28), 31.3 (C14), 30.6, 30.1, 28.6, 27.5 (C9, C10, C11, C12, C13), 24.9 (C8); IR (neat, ν_max_/cm^−^^1^) 3359 (strong broad, OH), 1703 (strong, NC=O); HRMS (ESI, deconvoluted): *m*/*z* calcd for C_548_H_970_N_136_O_144_ [M + 10H]^10+^: 1176.8321, found 1176.8032; [α]_D_^20^ = −4.7 (c 0.8, CH_3_OH).

#### 3.1.10. Synthesis of Br^−^@(DNJ-OAc-C6)_12_BU[6].Na^+^ **18**

According to procedure B and starting with propargyl_12_bambus[6]uril **2** (8.4 mg, 4.90 µmol, 1 eq.) and azidoiminosugar **12** (35.9 mg, 78.60 µmol, 16 eq.), sodium ascorbate (19.7 µL, 19.70 µmol, 4 eq.) and copper sulfate (9.8 µL, 9.80 µmol, 2 eq.) were added. After purification, Br^-^@(DNJ-OAc-C6)_12_BU[6].Na^+^ **18** was obtained (23.8 mg, 70% yield). ^1^H NMR (400 MHz, CDCl_3_): δ (ppm) 7.49 (s, 12H, H13), 5.79 (s, 12H, H17), 5.06–4.93 (m, 60H, H2, H3, H4, H15), 4.66–4.58 (br s, 12H, H19), 4.20–4.14 (m, 48H, H6, H12), 3.18–3.16 (m, 12H, H1′a), 2.63–2.61 (m, 12H, H7a), 2.60–2.52 (m, 12H, H5), 2.51–2.49 (m, 12H, H7b), 2.28 (t, *J* = 10.8 Hz, 12H, H1b), 2.05–2.02–2.01–2.00 (s, 144H, CH_3_-C=O), 1.85–1.82 (br s, 24H, H11), 1.39–1.24 (m, 72H, H8, H9, H10); ^13^C NMR (100 MHz, CDCl_3_): δ (ppm) 170.9–170.4–170.1–169.8 (CH_3_-C=O), 159.0 (C16, C18), 145.6 (C14), 122.8 (C13), 74.6 (C4), 69.3 (C17), 69.1 (C3), 61.7 (C5), 59.5 (C6), 53.6 (C1), 52.8 (C7), 51.9 (C12), 50.4 (C2), 47.6 (C19), 39.4 (C15), 30.2 (C11), 26.7 (C9), 26.6 (C10), 24.7 (C8), 21.0, 20.9, 20.8 (CH_3_-C=O); IR (neat, ν_max_/cm^−^^1^) 2939 (CH_2_), 1742 (CH_3_-C=O), 1703 (NC=O), 1478 (CH_2_), 1218 (C-N); HRMS (ESI+): *m*/*z* calculated for [C_306_H_444_N_72_O_108_+6H^+^]^6+^: 1143.5317, found 1143.5304; [α]_D_^20^ = 7.2 (c 0.18, CHCl_3_).

#### 3.1.11. Synthesis of Br^−^@(DNJ-OAc-C9)_12_BU[6] Na^+^ **19**

According to procedure B, propargyl_12_bambus[6]uril **2** (7.9 mg, 4.60 µmol, 1 eq.), azidoiminosugar **13** (36.8 mg, 73.80 µmol, 16 eq.), sodium ascorbate (18.4 µL, 18.40 µmol, 4 eq.) and copper sulfate (9.2 µL, 9.20 µmol, 2 eq.) were added. After purification, Br^-^@(DNJ-OAc-C9)_12_BU[6].Na^+^ **19** was obtained (25.1 mg, 74%). ^1^H NMR (400 MHz, CDCl_3_): δ (ppm) = 7.46 (br s, 12H, H16), 5.78 (br s, 12H, H20), 5.06–4.94 (m, 48H, H2, H3, H4, H22), 4.62 (br s, 24H, H18), 4.18–4.13 (m, 48H, H6, H15), 3.17 (dd, *J* = 11.4, 5.0 Hz, 12H, H1a), 2.74–2.68 (m, 12H, H7a), 2.63–2.58 (m, 12H, H5), 2.57–2.52 (m, 12H, H7b), 2.31 (t, *J* = 11.0 Hz, 12H, H1b), 2.06, 2.01, 2.01, 1.99 (s, 144H, CH_3_-C=O), 1.84–1.74 (m, 24H, H14), 1.37–1.23 (m, 144H, H8, H9, H10, H11, H12, H13); ^13^C NMR (100 MHz, CDCl_3_): δ (ppm) = 171.0, 170.5, 170.1, 169.9 (CH_3_-C=O), 160.6, 159.0 (C19, C21), 145.4 (C17), 122.2 (C16), 74.8 (C4), 69.5 (C2, C3, C20), 61.5 (C5), 59.6 (C6), 53.0 (C1), 51.9 (C7), 50.4 (C15), 47.8 (C22), 39.1 (C18), 30.3 (C14), 29.6, 29.1, 27.3, 26.8 (C9, C10, C11, C12, C13), 24.7 (C8), 21.0, 20.9, 20.8, 20.7 (CH_3_-C=O); IR (neat, ν_max_/cm^−^^1^) 2930 (CH_2_), 1743 (CH_3_-C=O), 1704 (NC=O), 1478 (CH_2_), 1225 (C-N), 1032 (C-O); HRMS (ESI+): *m*/*z* calculated for [C_342_H_516_N_72_O_108_+7H^+^]^7+^: 1052.3944, found 1052.3943; [α]_D_^20^ = 6.0 (c 0.24, CHCl_3_).

#### 3.1.12. Synthesis of (DNJ-C6)_12_BU[6] **6**

To a solution of Br^−^@(DNJ-OAc-C6)_12_BU[6].Na^+^ **18** (18.8 mg, 2.7 µmol, 1 eq.) in EtOAc (2 mL), AgSbF_6_ (1.6 mg, 5.4 µmol, 2 eq.) was added and the mixture was sonicated at 25 °C for 1 h to generate a white precipitate of AgBr. NH_4_OH (2 mL, 30% aqueous solution) was then added and the organic solution was separated, washed with water (2 × 2 mL) and concentrated under vacuum to give corresponding anion-free BU that was directly solubilized in H_2_O/MeOH (1/1, 2 mL). Amberlite IRN 78 (HO^−^) (125 mg) was added and the suspension was heated for 12 h at 40° C. The mixture was diluted with MeOH (1 mL) and the resin was removed by filtration before being washed with H_2_O (1 mL) and MeOH (1 mL). The filtrate was concentrated under vacuum to afford anion-free (DNJ-C6)_12_BU[6] **6** (12.2 mg, 94% yield). ^1^H NMR (400 MHz, D_2_O): δ (ppm) = 7.93 (br s, 12H, H13), 5.73 (br s, 12H, H17), 4.67–4.35 (m, 36H, H15, H19), 4.34–4.13 (m, 24H, H12), 3.89–3.77 (m, 24H, H6), 3.53 (td, *J* = 10.2, 5.0 Hz, 12H, H2), 3.38 (t, *J* = 9.6 Hz, 12H, H3), 3.25 (t, *J* = 9.4 Hz, 12H, H4), 2.97 (dd, *J* = 11.1, 4.6 Hz, 12H, H1a), 2.75–2.61 (m, 12H, H7a), 2.59–2.47 (m, 12H, H7a), 2.28–2.17 (m, 24H, H1b, H5), 1.86–1.72 (m, 24H, H11), 1.43–1.31 (m, 24H, H8), 1.23–1.12 (m, 48H, H9, H10); ^13^C NMR (100 MHz, D_2_O): δ (ppm) = 159.8, 158.8 (C16, C18), 144.4 (C14), 123.3 (C13), 78.3 (C4), 69.9 (C17), 68.8 (C3), 68.5 (C2), 64.9 (C5), 57.4 (C6), 55.4 (C1), 51.8 (C7), 50.3 (C12), 49.2 (C19), 38.9 (C15), 29.3 (C11), 26.1 (C9), 25.5 (C10), 22.6 (C8); IR (neat, ν_max_/cm^−^^1^) 3337 (OH), 2923 (CH_2_), 1703 (NC=O), 1484 (CH_2_), 1216 (C-N), 1064 (C-O); HRMS (ESI+): *m*/*z* calculated for [C_210_H_348_N_72_O_60_+6H^+^]^6+^: 807.4472, found 807.4462; [α]_D_^20^ = 1.7 (c 0.16, H_2_O).

#### 3.1.13. Synthesis of (DNJ-C9)_12_BU[6] **7**

To a solution of (DNJ-OAc-C9)_12_BU[6] **19** (23.4 mg, 3.1 µmol, 1 eq.) in EtOAc (2 mL), AgSbF_6_ (1.83 mg, 6.3 µmol, 2 eq.) was added and the mixture was sonicated at 25 °C for 1 h to generate a white precipitate of AgBr. NH_4_OH (2 × 1 mL, 30% aqueous solution) was then added and the organic solution was separated, washed with water (2 × 1 mL) and concentrated under vacuum to give corresponding anion-free BU that was solubilized in H_2_O/MeOH (1/1, 2 mL). Amberlite IRN 78 (HO^−^) (135 mg) was added and the suspension was heated for 12 h at 40 °C. The mixture was diluted with MeOH (1 mL) and the resin was removed by filtration and washed with H_2_O (1 mL) and MeOH (1 mL). The filtrate was concentrated under vacuum to afford anion-free (DNJ-C9)_12_BU[6] **7** (15.6 mg, 93% yield). ^1^H NMR (400 MHz, D_2_O/MeOD 0.3/0.2): δ (ppm) = 7.84 (br s, 12H, H16), 5.72 (br s, 12H, H20), 4.63–4.29 (m, 36H, H18, H22), 4.26–4.16 (m, 24H, H15), 3.83–3.78 (m, 24H, H6), 3.52–3.48 (m, 12H, H4), 3.37–3.31 (m, 12H, H2), 3.22–3.18 (m, 12H, H3), 2.95 (d, *J* = 6.3 Hz, 12H, H1a), 2.70–2.59 (m, 12H, H7a), 2.58–2.48 (m, 12H, H7b), 2.24–2.14 (m, 24H, H1b, H5), 1.78–1.72 (m, 24H, H14), 1.42–1.35 (m, 24H, H8), 1.24–0.95 (m, 120H, H9, H10, H11, H12, H13); ^13^C NMR (150 MHz, D_2_O/MeOD 0.3/0.2): δ (ppm) = 161.3, 160.3 (C19-C21), 146.2 (C17), 124.8 (C16), 79.9 (C4), 71.4 (C20), 70.2 (C2, C3), 66.8 (C5), 58.9 (C6), 57.0 (C1), 53.9 (C7), 51.8 (C22), 40.5 (C15, C18), 31.2 (C14), 30.5, 30.0, 28.5, 27.5 (C9, C10, C11, C12, C13), 24.5 (C8); IR (neat, ν_max_/cm^−^^1^) 3370 (OH), 2926 (CH_2_), 1708 (NC=O), 1472 (CH_2_), 1215 (C-N), 1046 (C-O); HRMS (ESI+): *m*/*z* calculated for [C_246_H_420_N_72_O_60_+9H^+^]^9+^: 594.6965, found 594.6973; [α]_D_^20^ = 5.9 (c = 0.18 H_2_O/MeOH 3/7).

#### 3.1.14. Synthesis of Br^−^@(DNJ-C6)_12_BU[6].TBA^+^ **8**

To a solution of (DNJ-C6)_12_BU[6] **6** (9.1 mg, 1.88 µmol, 1 eq.) in 0.5 mL of H_2_O, a solution of TBABr in H_2_O (64.1 µL, c = 29.3 μM/mL, 1 eq.) was added. The mixture was sonicated for 30 min and was lyophilized to afford Br^−^@(DNJ-C6)_12_BU[6].TBA^+^ **8** (9.5 mg, 98% yield). ^1^H NMR (400 MHz, D_2_O): δ (ppm) = 7.93 (br s, 12H, H13), 5.81 (br s, 12H, H17), 4.72–4.53 (m, 36H, H15, H19), 4.32–4.28 (m, 24H, H12), 3.82–3.78 (m, 24H, H6), 3.58–3.47 (m, 12H, H2), 3.37 (t, *J* = 9.5 Hz, 12H, H3), 3.29–3.24 (m, 12H, H4), 3.24–3.15 (8H, m, N-(CH_2_-CH_2_-CH_2_-CH_3_)_4_, 2.98–2.93 (m, 12H, H1a), 2.74–2.63 (m, 12H, H7a), 2.52–2.41 (m, 12H, H7b), 2.32–2.18 (m, 32H, H1b, H5, N-(CH_2_-CH_2_**-**CH_2_-CH_3_)_4_), 1.91–1.78 (m, 24H, H11), 1.68–1.59 (m, 8H, N-(CH_2_-CH_2_-CH_2_-CH_3_)_4_) 1.38–1.16 (m, 72H, H8, H9, H10), 0.94 (t, *J* = 7.3 Hz, 12H, N-(CH_2_-CH_2_-CH_2_-CH_3_)_4_; ^13^C NMR (100 MHz, D_2_O): δ (ppm) = 160.3, 159.6 (C16, C18), 145.3 (C14), 123.9 (C13), 78.9 (C4), 70.5 (C17), 69.4 (C3), 65.7 (C2), 58.7 (N-CH_2_**-**CH_2_-CH_2_-CH_3_), 57.9 (C5), 55.9 (C6), 52.5 (C1), 50.9 (C7), 48.1 (C12, C19), 39.8 (C15), 29.9 (N-CH_2_-CH_2_**-**CH_2_-CH_3_), 26.8 (C11), 26.2 (C9), 23.8 (C10), 23.3 (C8), 19.8 (N-CH_2_-CH_2_-CH_2_-CH_3_), 13.4 (N-CH_2_-CH_2_-CH_2_-CH_3_); IR (neat, ν_max_/cm^−^^1^) 3374 (OH), 2928 (CH_2_), 1701 (NC=O), 1481 (CH_2_), 1215 (C-N), 1055 (C-O); [α]_D_^20^ = + 3.5 (c 0.14, H_2_O).

#### 3.1.15. Synthesis of I^-^@(DNJ-C6)_12_BU[6].TBA^+^ **9**

To a solution of (DNJ-C6)_12_BU[6] **6** (8.5 mg, 1.75 µmol, 1 eq.) in 0.5 mL of H_2_O was added a solution of TBAI in H_2_O (65.9 µL, c = 26.5 μM/mL, 1 eq.). The reaction mixture was sonicated for 30 min and then lyophilized to afford I^-^@(DNJ-C6_12_BU[6].TBA^+^ **9** (8.8 mg, 97% yield). ^1^H NMR (400 MHz, D_2_O): δ (ppm) = 7.93 (br s, 12H, H13), 5.98 (br s, 12H, H17), 4.72–4.53 (m, 36H, H15, H19), 4.38–4.29 (m, 24H, H12), 3.83–3.80 (m, 24H, H6), 3.57–3.48 (m, 12H, H2), 3.37 (t, *J* = 9.6 Hz, 12H, H3), 3.28–3.21 (m, 20H, H4, N-(**CH_2_**-CH_2_-CH_2_-CH_3_)_4_), 3.01–2.95 (m, 12H, H1a), 2.70–2.59 (m, 12H, H7a), 2.58–2.49 (m, 12H, H7b), 2.25–2.18 (m, 32H, H1b, H5, N-(CH_2_-**CH_2_-**CH_2_-CH_3_)_4_), 1.88–1.70 (m, 24H, H11), 1.66–1.61 (m, 8H, N-(CH_2_-CH_2_-**CH_2_**-CH_3_)_4_), 1.39–1.17 (m, 72H, H8, H9, H10), 0.95 (t, *J* = 7.4 Hz, 12H, N-(CH_2_-CH_2_-CH_2_-**CH_3_**)_4_; ^13^C NMR (100 MHz, D_2_O): δ (ppm) = 160.9, 159.9 (C16, C18), 145.4 (C14), 123.9 (C13), 78.9 (C4), 70.6 (C17), 69.9 (C3), 65.7 (C2), 58.7 ((N-**CH_2_-**CH_2_-CH_2_-CH_3_), 58.1 (C5), 56.0 (C6), 52.5 (C1), 50.9 (C7), 47.9 (C12, C19), 40.0 (C15), 30.0 (N-CH_2_-**CH_2_-**CH_2_-CH_3_), 26.8 (C11), 26.2 (C9), 23.8 (C10), 23.3 (C8), 19.8 (N-CH_2_-CH_2_-**CH_2_**-CH_3_), 13.4 (N-CH_2_-CH_2_-CH_2_-**CH_3_**); IR (neat, ν_max_/cm^−^^1^) 3331 (OH), 2920 (CH_2_), 1702 (NC=O), 1482 (CH_2_), 1216 (C-N), 1067 (C-O); [α]_D_^20^ = + 4.2 (c 0.12, H_2_O).

#### 3.1.16. Synthesis of Br^−^@(DNJ-C9)_12_BU[6].TBA^+^ **10**

To a solution of anion-free (DNJ-C9)_12_BU[6] **7** (10.8 mg, 2.02 µmol, 1 eq.) in H_2_O (1 mL) was added a solution of TBABr in H_2_O (63.6 µL, c = 31.8 μM/mL 1 eq.). The reaction mixture was sonicated for 30 min and then was lyophilized to afford Br^-^@(DNJ-C9)_12_BU[6].TBA^+^ **10** (11.2 mg, 98% yield). ^1^H NMR (400 MHz, D_2_O/MeOD 0.35/0.2): δ (ppm) = 7.87 (br s, 12H, H16), 5.77 (br s, 12H, H20), 4.62–4.52 (m, 36H, H18, H22), 4.29–4.24 (m, 24H, H15), 3.85–3.81 (m, 24H, H6), 3.55–3.48 (m, 12H, H4), 3.37–3.33 (m, 12H, H2), 3.24–3.19 (m, 20H, H3, N-(**CH_2_**-CH_2_-CH_2_-CH_3_)_4_), 2.98 (d, *J* = 6.7 Hz, 12H, H1a), 2.77–2.63 (m, 12H, H7a), 2.62–2.50 (m, 12H, H7b), 2.33–2.12 (m, 24H, H1b, H5), 1.82–1.78 (m, 24H, H14), 1.66–1.59 (m, 8H, N-(CH_2_-CH_2_-**CH_2_**-CH_3_)_4_, 1.50–1.31 (m, 32H, H8, N-(CH_2_-**CH_2_-**CH_2_-CH_3_)_4_), 1.25–1.16 (m, 120H, H9, H10, H11, H12, H13), 0.96 (t, *J* = 7.4 Hz, 12H, N-(CH_2_-CH_2_-CH_2_-**CH_3_**)_4_); ^13^C NMR (176 MHz, D_2_O/MeOD 0.35/0.2): δ (ppm) = 160.8, 160.0 (C19-C21), 145.9 (C17), 124.3 (C16), 79.5 (C4), 71.0 (C20), 69.9 (C2, C3), 66.4 (C5), 59.1 (N-**CH_2_-**CH_2_-CH_2_-CH_3_), 58.5 (C6), 56.7 (C1), 53.4 (C7), 51.3 (C22), 40.3 (C15, C18), 30.8 (C14), 30.0 (N-CH_2_-**CH_2_-**CH_2_-CH_3_), 29.5–28.0–27.0–24.3–23.9 (C8, C9, C10, C11, C12, C13), 20.3 (N-CH_2_-CH_2_-**CH_2_**-CH_3_), 13.9 ((N-CH_2_-CH_2_-CH_2_-**CH_3_**). IR (neat, ν_max_/cm^−^^1^) 3375 (OH), 2922 (CH_2_), 1702 (NC=O), 1482 (CH_2_), 1131 (C-N), 1057 (C-O). [α]_D_^20^ = + 3.8 (c 0.10, H_2_O/MeOH 0.3/0.2).

#### 3.1.17. Synthesis of Br^−^@(DNJ-OAc-Tripod)_6_BU[6] Na^+^ **20**

To a solution of propargylated bambusuril **2** (6.0 mg, 3.53 µmol, 1 eq.) in H_2_O/*t*BuOH (1.2 mL, 0.5/0.7) were added tripod-azide **14** (105.5 mg, 56.74 µmol, 16 eq.), sodium ascorbate (14.2 µL of 1 M aqueous solution, 14.20 µmol, 4 eq.) and copper sulfate (7.1 µL of 1 M aqueous solution, 7.10 µmol, 2 eq.). The reaction was stirred and heated under microwave irradiations for 3 h at 80 °C with a power of 50 W. A solution of MeCN/H_2_O/NH_4_OH (30w%) (1 mL, 15/0.5/0.5, *v*/*v*/*v*) was then added and the mixture was filteredon a small pad of SiO_2_. The filtrate was concentrated under vacuum and the crude product was purified on silica gel chromatography (CH_2_Cl_2_/MeOH, 99:1 to 95:5) to afford Br^−^@(DNJ-OAc-Tripod)_6_BU[6].Na^+^ **20** (37.5 mg, 84% yield). ^1^H NMR (400 MHz, CDCl_3_): δ (ppm*)* = 7.64–7.45 (m, 24H, H16, H26), 5.90–5.65 (br s, 12H, H33), 5.17–4.83 (m, 54H, H2, H3, H4), 4.55–4.22 (m, 120H, H15, H18, H25, H28, H30, H35), 4.18–4.10 (m, 36H, H6), 3.83–3.60 (m, 12H, H24), 3.48–3.28 (m, 72H, H19, H21, H22, H23), 3.24–3.10 (m, 18H, H1a), 2.80–2.47 (m, 54H, H5, H7), 2.38–2.25 (m, 18H, H1b), 2.11 (br s, 6H, H32), 2.10–1.88 (m, 252H, CH_3_-C=O, H14), 1.85–1.74 (m, 36H, H8), 1.51–1.29 (m, 36H, H8), 1.29–1.19 (m, 180H, H9, H10, H11, H12, H13); ^13^C NMR (176 MHz, CDCl_3_): δ (ppm) = 170.8, 170.3, 169.9, 169.7 (CH_3_-C=O), 159.2 (C29, C34), 145.2 (C17, C27), 123.9 (C16), 122.9 (C26), 74.1 (C3), 71.2–70.3–69.9 (C21, C22, C23), 69.3 (C4, C24), 68.8 (C2, C19, C33), 65.0 (C18), 61.6 (C5), 58.9 (C6), 52.1 (C1), 50.4 (C7, C15, C25), 47.8 (C35), 45.5 (C20), 39.4 (C28), 30.5 (C14), 29.8, 29.4, 29.1, 27.1, 26.6 (C9, C10, C11, C12, C13), 24.3 (C8), 21.0, 20.9, 20.8, 20.7 (CH_3_-C=O); IR (neat, ν_max_/cm^−^^1^) 2927 (CH_2_), 1745 (CH_3_-C=O), 1704 (NC=O), 1471 (C-N), 1225 (C-H), 1032 (C-O); HRMS (ESI+): *m*/*z* calculated for [C_588_H_894_N_114_O_186_+9H]^9+^: 1393.0446, found 1393.8317; [α]_D_^20^ = 9.9 (c 0.105, CHCl_3_).

#### 3.1.18. Synthesis of Br^−^@(DNJ-Tripod)_6_BU[6].Na^+^ **11**

To a solution of Br^-^@(DNJ-OAc-Tripod)_6_BU[6].Na^+^ **20** (19.5 mg, 1.54 µmol, 1 eq.) in H_2_O/MeOH (1 mL 1/1), amberlite IRN 78 (HO^−^) (59 mg) was added and the mixture was heated at 40 °C for 12 h. After concentration under vacuum, Br^-^@(DNJ-Tripod)_6_BU[6].Na^+^ **11** was obtained (12.5 mg, 84% yield). ^1^H NMR (D_2_O/MeOD 50/50, 400 MHz): δ (ppm) 7.94 (br s, 24H, H16, H26), 5.96–5.77 (m, 12H, H33), 4.50–4.26 (m, 90H, H2, H3, H4, H25, H28, H30), 3.85 (br s, 36H, H18), 3.69 (s, 12H, H35), 3.59–3.51 (m, 36H, H15), 3.43–3.28 (m, 84H, H19, H21, H22, H23, H24), 3.22 (t, *J* = 9.0 Hz, 36H, H6), 3.03–2.98 (m, 18H, H1a), 2.79–2.69 (m, 18H, H7a), 2.68–2.56 (m, 18H, H7b), 2.27–2.16 (m, 42H, H1, H5, H32), 1.86–1.78 (m, 36H, H14), 1.51–1.35 (m, 36H, H8), 1.37–1.11 (m, 180H, H9, H10, H11, H12, H13); ^13^C NMR (176 MHz, D_2_O/MeOD 50/50): δ (ppm) 159.4 (C29, C34), 145.6 (C17, C27), 125.2 (C16, C26), 79.7 (C3), 71.2 (C21, C22, C23), 70.0 (C4), 69.5 (C24), 66.4 (C2, C19, C33), 64.9 (C18), 58.7 (C5), 56.8 (C6), 53.4 (C1), 51.3 (C7, C15, C25), 46.1 (C35), 45.2 (C20), 40.1 (C28), 30.9 (C14), 30.1, 29.9, 29.6, 28.2, 27.1 (C9, C10, C11, C12, C13), 24.1 (C8); IR (neat, ν_max_/cm^−^^1^): 3550 (OH), 2922 (CH_2_), 1702 (NC=O), 1394 (C-N), 1249 (CH), 1065 (C-O); [α]_D_^20^ = +1.8 (c 0.055, H_2_O/MeOH 1/1); HRMS MS(ESI+): *m*/*z* calculated for (C_444_H_750_N_114_O_114_+13H]^13+^: 732.0492, found 732.8294.

### 3.2. Isothermal Titration Calorimetry Experiments

ITC measurements were performed with a VP-ITC microcalorimeter (Microcal, GEHealthcare). Experiments were carried out in water and in a solution of K_2_HPO_4_ (1.5 mM) in mili-Q water at 298.15 ± 0.1 K. Anion binding to BU was investigated via a classical isothermal titration experiment (10 µL additions) and a single injection method (SIM). Injection of sodium iodide solution was added automatically to the BU solution present in the calorimeter cell while stirring at 307 rpm. Integrated heat effects were analyzed by non-linear regression using a single-site binding model (Microcal Origin 7). The experimental data fitted to a theorical titration curve, giving the association constant *K_a_*, the enthalpy of binding ΔH° and the entropy ΔS°. The free energy ΔG° was calculated from the equation: ΔG° = ΔH° − *T*ΔS°, where *T* is the absolute temperature. The first smaller addition (2 µL), which was used to compensate for diffusion of the guest from the injector during equilibration, was discarded prior to data fitting.

### 3.3. Inhibition Assays on Jack-Bean α-Mannosidase

*p*-nitrophenyl-α-D-mannopyranoside and α-mannosidase (EC 3.2.1.24, from Jack Bean) were purchased from Sigma Aldrich, St. Louis, MO, USA. Inhibition constants were determined spectrophotometrically by measuring the residual hydrolytic activities of the α-mannosidase against *p*-nitrophenyl-α-D-mannopyranoside in the presence and absence of an inhibitor. Each well was filled with a total volume of 100 µL, containing 0.2 M acetate buffer pH 5, inhibitor, substrate and enzyme. All kinetics were performed between 25 °C and 27 °C and started by enzyme addition. After 30–50 min of incubation, the reaction was quenched by addition of 100 µL of 1M Na_2_CO_3_. The absorbance of the resulting solution was determined at 405 nm. *K*_i_ values were determined in triplicate, using the Dixon and Lineweaver–Burk graphical methods within Microsoft Excel. Stock solutions of inhibitors were prepared with DMSO/buffer for final well DMSO content under 5%. The stability of the enzyme in the presence of the same concentrations of DMSO was controlled and enzyme activity was unaffected.

## 4. Conclusions

Propargylated bambus[4,6]urils were fully functionalized by click chemistry with linear *N*-alkylDNJ ligands either having 6 or 9 carbon alkyl azido linkers or a trivalent DNJ derivative to generate multivalent clusters bearing up to 24 iminosugars. The novelty of this study lies in the unique combination of anion-transporting BU[6] scaffolds with iminosugar inhitopes that have already proven their efficiency towards the multivalent inhibition of glycosidases. For clusters grafted with DNJ ligands linked by short C6 arms, scaffold size showed a strong impact on α-mannosidase inhibition, with the best results obtained for BU[4] clusters. With nonyl chains, the enzyme affinity per inhibitory head increases drastically whichever platform is used, the best affinity increases being obtained with trivalent dendrons providing higher local inhibitor concentration. Bromide and iodide sequestered in BU[6] grafted by DNJ inhibitors were also prepared and their activity was compared to their corresponding anion-free counterparts, showing that the presence of anions neither modified the inhibition potency of the clusters nor their inhibition mode. Our study thus shows a first proof of concept for glycosidase-directed ion caging agents based on unprecedented bambusuril-based iminosugar clusters. Altogether, these results open the way to unique water-soluble probes/therapeutic agents with both strong glycosidase inhibition potency and ion receptor properties.

## Figures and Tables

**Table 1:** Inhibition activity of (DNJ)_8_BU[4] **3**–**5** and (DNJ)_12_BU[6] **6**–**11** towards Jack Bean α-mannosidase.

Entry	Compound	BU	Alkyl Chain Length	DNJ Units	*K_i_* (µM)	Inhibition Mode	*Rp* ^[a]^	*rp/n*
1	**21**	-	C6	1	322 [22]	competitive	-	-
2	**22**	-	C9	1	188 [37]	competitive	-	-
3	**3**	BU[4]	C6	8	9.7+/−2.9	competitive	33	4
4	**4**	BU[4]	C9	8	0.71+/−0.55	competitive	265	33
5	**5**	BU[4]	C9	24	0.024+/−0.004	competitive	7833	326
6	**6**	BU[6]	C6	12	68.2+/−10.3 147+/−21	mixed	4.7	0.4
7	**8**	Br^−^@BU[6].TBA^+^	C6	12	85.6+/−10.3 191+/−37	mixed	3.8	0.3
8	**9**	I^-^@BU[6].TBA^+^	C6	12	108+/−13 162+/−40	mixed	3.0	0.5
9	**7**	BU[6]	C9	12	1.06+/−0.34	competitive	177	15
10	**10**	Br^−^@BU[6].TBA^+^	C9	12	0.488+/−0.343	competitive	385	32
11	**11**	Br^−^@BU[6].Na^+^	C9	18	0.016+/−0.002	competitive	11750	~652

^[a]^*rp* = *K*_i_ (monovalent reference)/*K*_i_ (neoglycocluster), *n* = number of inhitope units.

## Data Availability

Not applicable.

## Supplementary Materials

The following supporting information can be downloaded at: https://www.mdpi.com/article/10.3390/molecules27154772/s1, Figure S1: NMR ^1^H and ^13^C spectra of compounds **3**, **4**, **5**, **6**, **7**, **8**, **9**, **10**, **11**, **15**, **16**, **17**, **18**, **19**, **20**, ITC of I^-^ binding to **6** and enzymatic study of **3**, **4**, **5**, **6**, **8**, **9**, **10** and **11**.
