# Supplementary material for: Bambus[4,6]urils as Dual Scaffolds for Multivalent Iminosugar Presentation and Ion Transport: Access to Unprecedented Glycosidase-Directed Anion Caging Agents
Source: Molecules. 2022 Jul 26;27(15):4772. doi: 10.3390/molecules27154772 (PMC9330389; doi:10.3390/molecules27154772)
Supplement: Supplementary file 1 [file molecules-27-04772-s001.zip › molecules-1816842-supplementary.pdf]

Bambus[4,6]urils As Dual Scaffolds for Multivalent Iminosugar Presentation and Ion Transport : Access to Unprecedented Glycosidase-directed Anion Caging Agents

Marie Lafosse [1], Yan Liang [2], Jérémy P. Schneider [2], Elise Cartier [1], Anne Bodlenner [2,*], Philippe Compain [2,*] and Marie-Pierre Heck [1,*]

Supporting Information

Table of Contents

^{1}H-NMR and ^{13}C-NMR spectra of (DNJ-OAc-C6)$_8$BU[4] **15** **S2**
^{1}H-NMR and ^{13}C-NMR spectra of (DNJ-OAc-C9)$_8$BU[4] **16** **S3**
^{1}H-NMR and ^{13}C-NMR spectra of (DNJ-OAc-tripod)$_8$BU[4] **17** **S4**
^{1}H-NMR and ^{13}C-NMR spectra of Br^-@(DNJ-OAc-C6)$_{12}$BU[6].Na^+ **18** **S5**
^{1}H-NMR and ^{13}C-NMR spectra of Br^-@(DNJ-OAc-C9)$_{12}$BU[6].Na^+ **19** **S6**
^{1}H-NMR and ^{13}C-NMR spectra of Br^-@(DNJ-OAc-Tripod)$_6$BU[6].Na^+ **20** **S7**
^{1}H-NMR and ^{13}C-NMR spectra of (DNJ-C6)$_8$BU[4] **3** **S8**
^{1}H-NMR and ^{13}C-NMR spectra of (DNJ-C9)$_8$BU[4] **4** **S9**
^{1}H-NMR and ^{13}C-NMR spectra of (DNJ-tripod)$_8$BU[4] **5** **S10**
^{1}H-NMR and ^{13}C-NMR spectra of (DNJ-C6)$_{12}$BU[6] **6** **S11**
^{1}H-NMR and ^{13}C-NMR spectra of (DNJ-C9)$_{12}$BU[6] **7** **S12**
^{1}H-NMR and ^{13}C-NMR spectra of Br^-@(DNJ-C6)$_{12}$BU[6].TBA^+ **8** **S13**
^{1}H-NMR and ^{13}C-NMR spectra of I^-@(DNJ-C6)$_{12}$BU[6].TBA^+ **9** **S14**
^{1}H-NMR and ^{13}C-NMR spectra of Br^-@(DNJ-C9)$_{12}$BU[6].TBA^+ **10** **S15**
^{1}H-NMR and ^{13}C-NMR spectra of Br^-@(DNJ-Tripod)$_6$BU[6].Na^+ **11** **S16**
Figure S1. Isothermal titration calorimetry of I^- binding to (DNJ-C6)$_{12}$BU[6] **6** **S17**
Figure S2 Lineweaver-Burk plot of (DNJ-C6)$_8$BU[4] **3** **S18**
Figure S3 . Lineweaver-Burk plot of compound (DNJ-C9)$_8$BU[4] **4** **S19**
Figure S4 Lineweaver-Burk plot of compound (DNJ-Tripod)$_8$BU[4] **5** **S20**
Figure S5. Lineweaver-Burk plot of compound (DNJ-C6)$_{12}$BU[6] **6** **S21**
Figure S6. Lineweaver-Burk plot of compound Br^-@(DNJ-C6)$_{12}$BU[6].TBA^+ **8** **S22**
Figure 7. Lineweaver-Burk plot of compound I^-@(DNJ-C6)$_{12}$BU[6].TBA^+ **9** **S23**
Figure S8 . Lineweaver-Burk plot of compound Br^-@(DNJ-C9)$_{12}$BU[6].TBA^+ **10** **S24**
Figure S9 Lineweaver-Burk plot of compound Br^-@(DNJ-Tripod)$_6$BU[6].Na^+ **11** **S25**

(DNJ-OAc-C6)$_8$BU[4] **15**

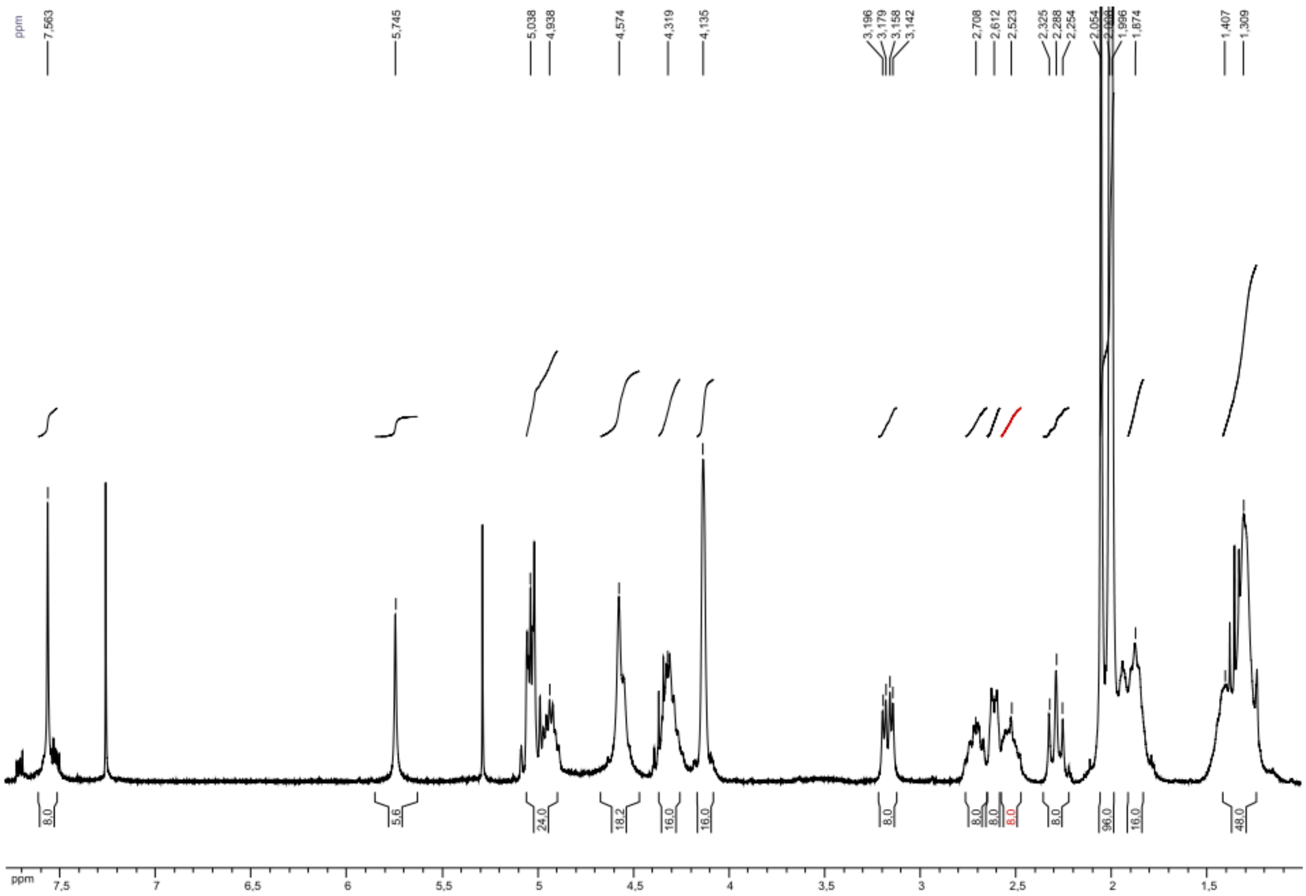

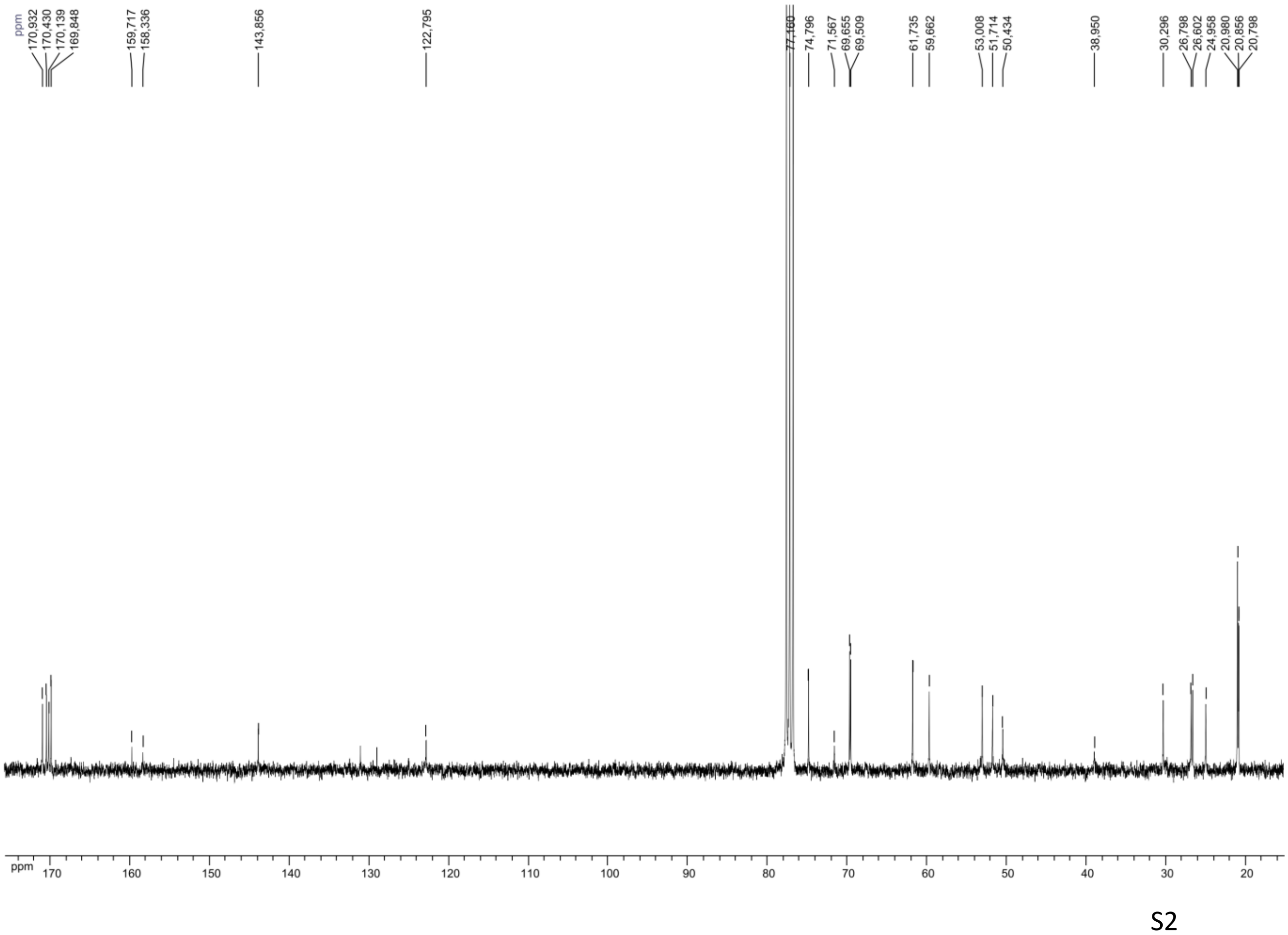

(DNJ-OAc-C9)$_8$BU[4] **16**

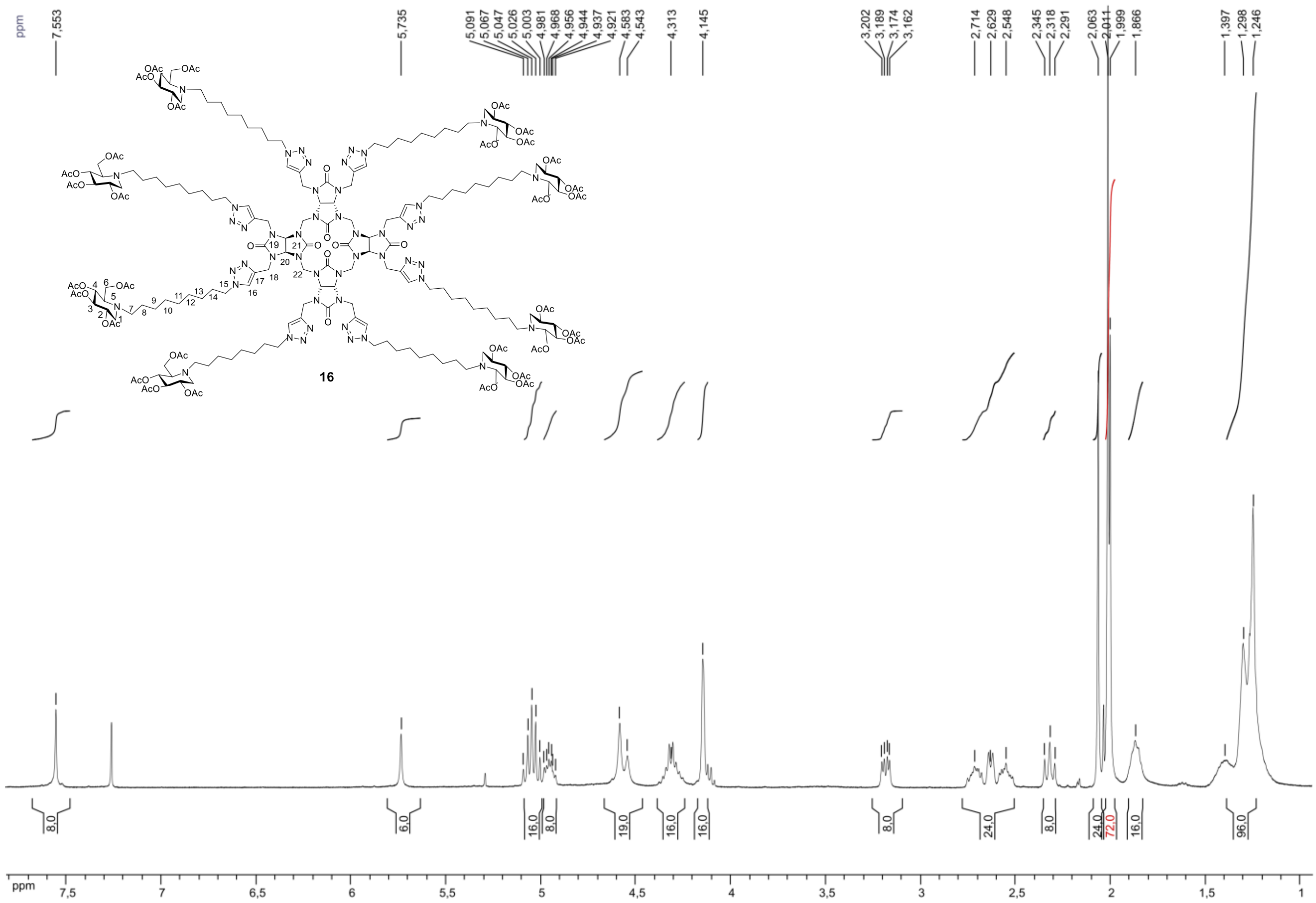

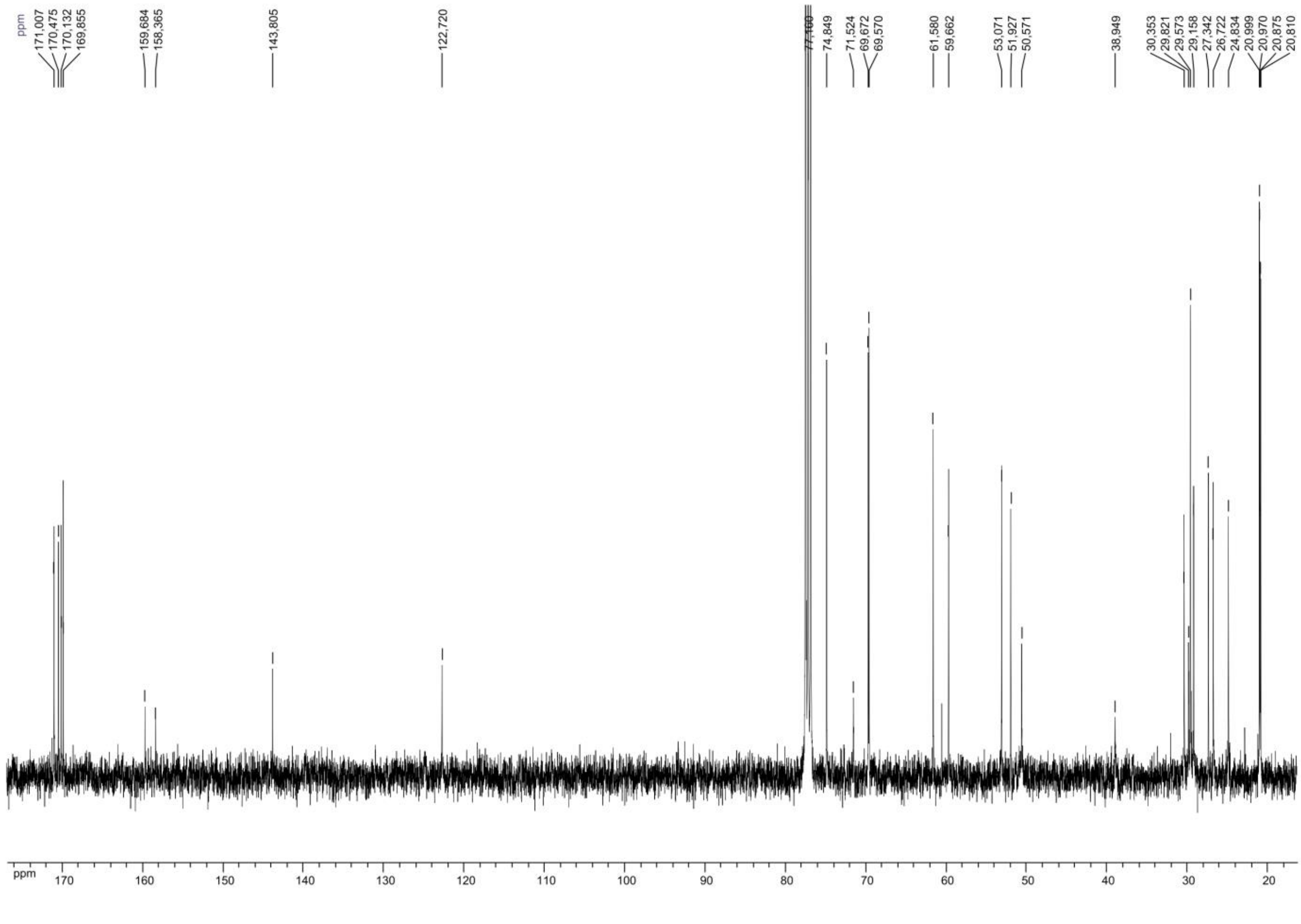

(DNJ-OAc-tripod)$_8$BU[4] **17**

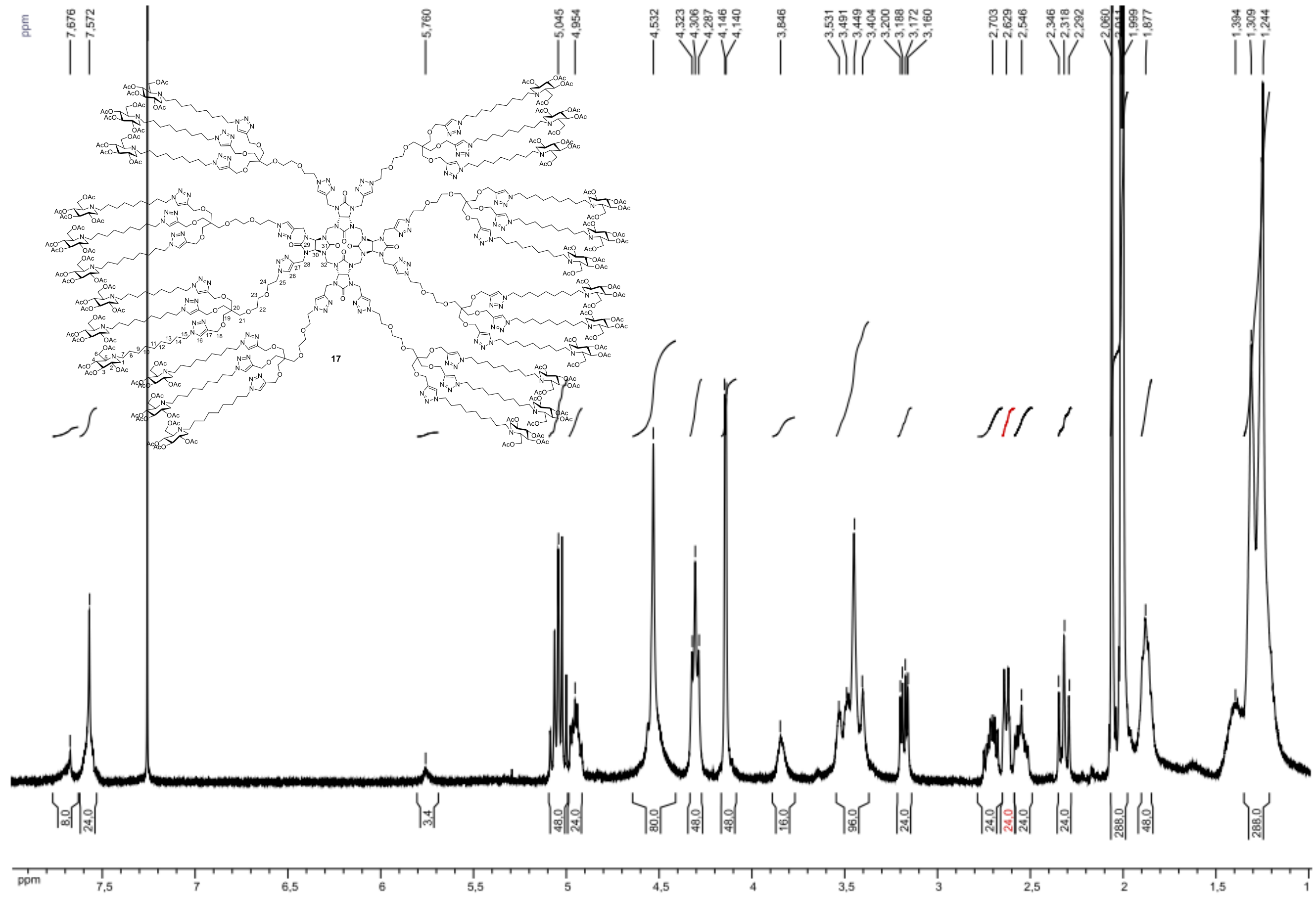

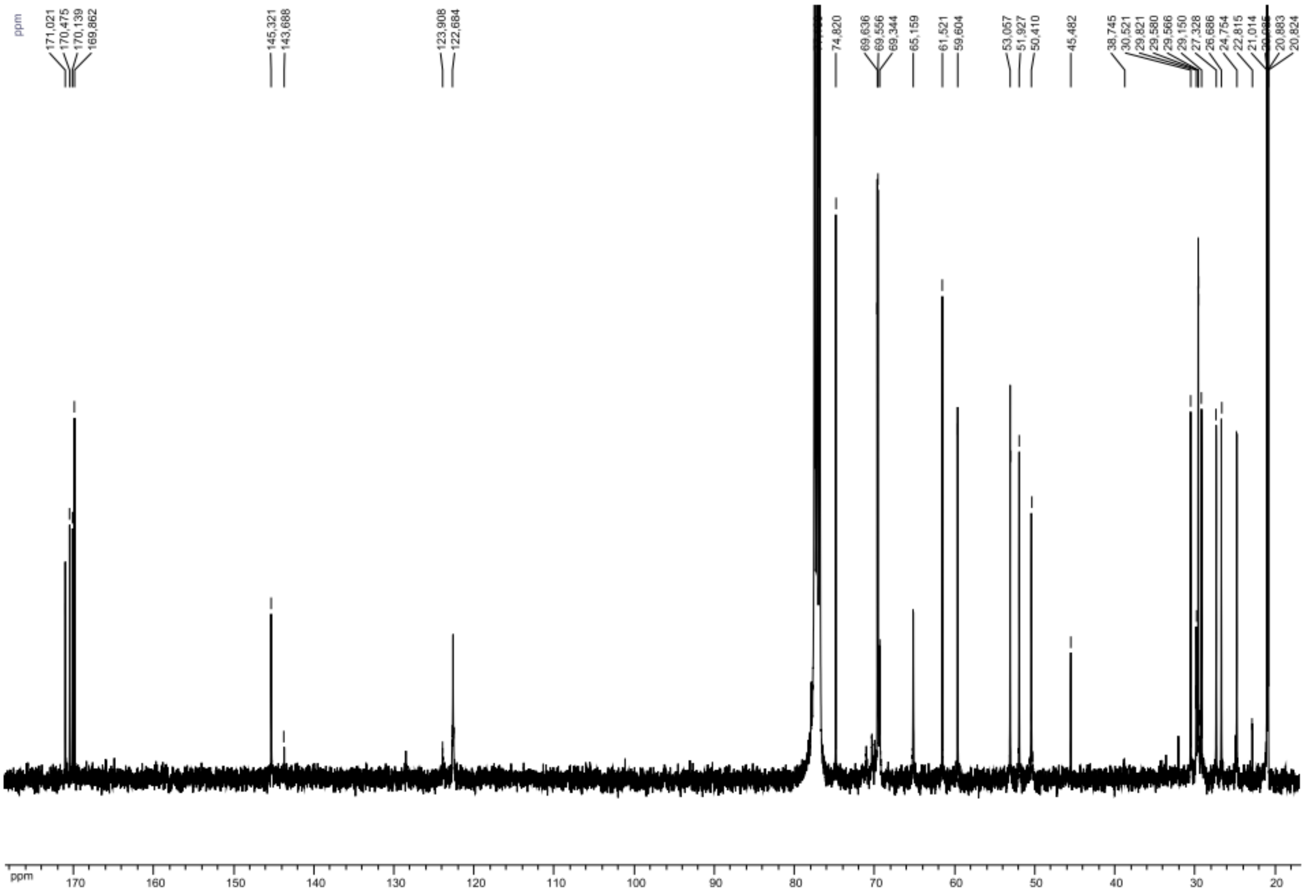

Br$^-$@(DNJ-OAc-C6)$_{12}$BU[6].Na$^+$ **18**

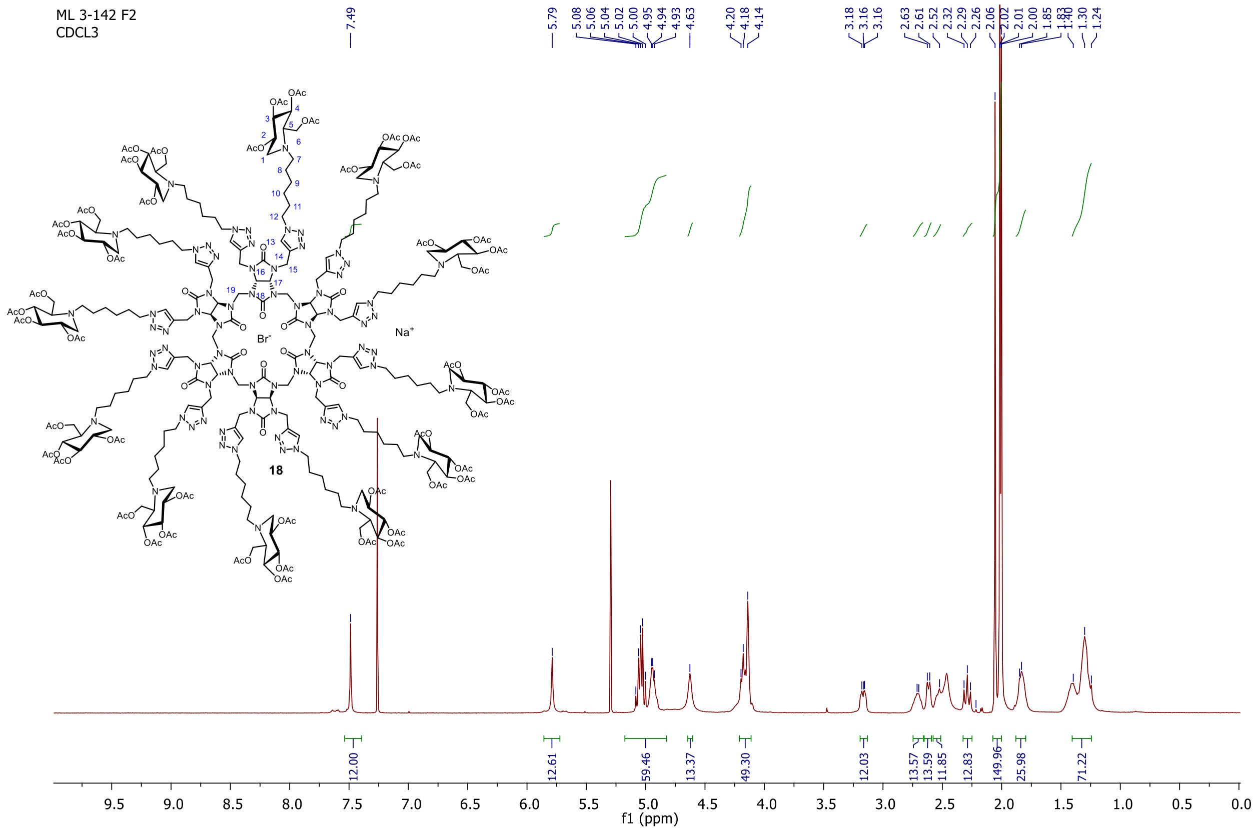

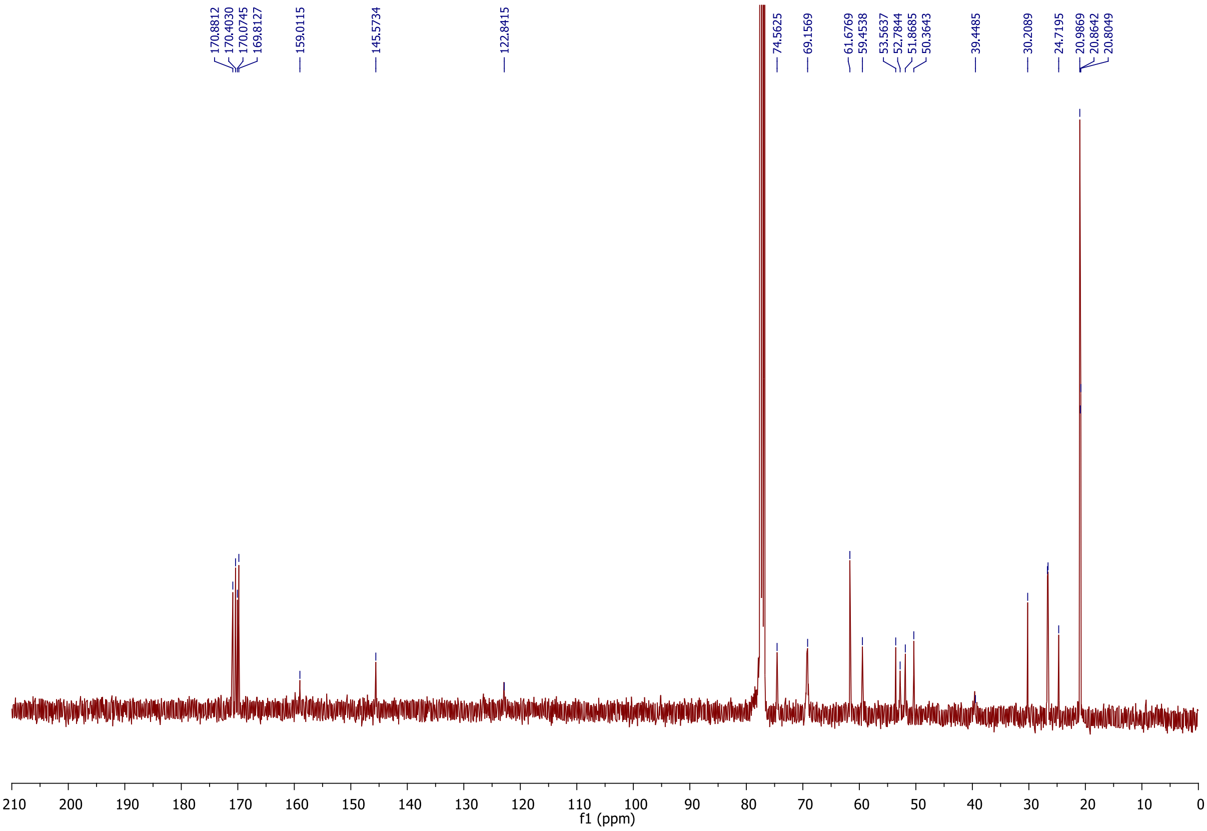

Br$^-$@(DNJ-OAc-C9)$_{12}$BU[6].Na$^+$ **19**

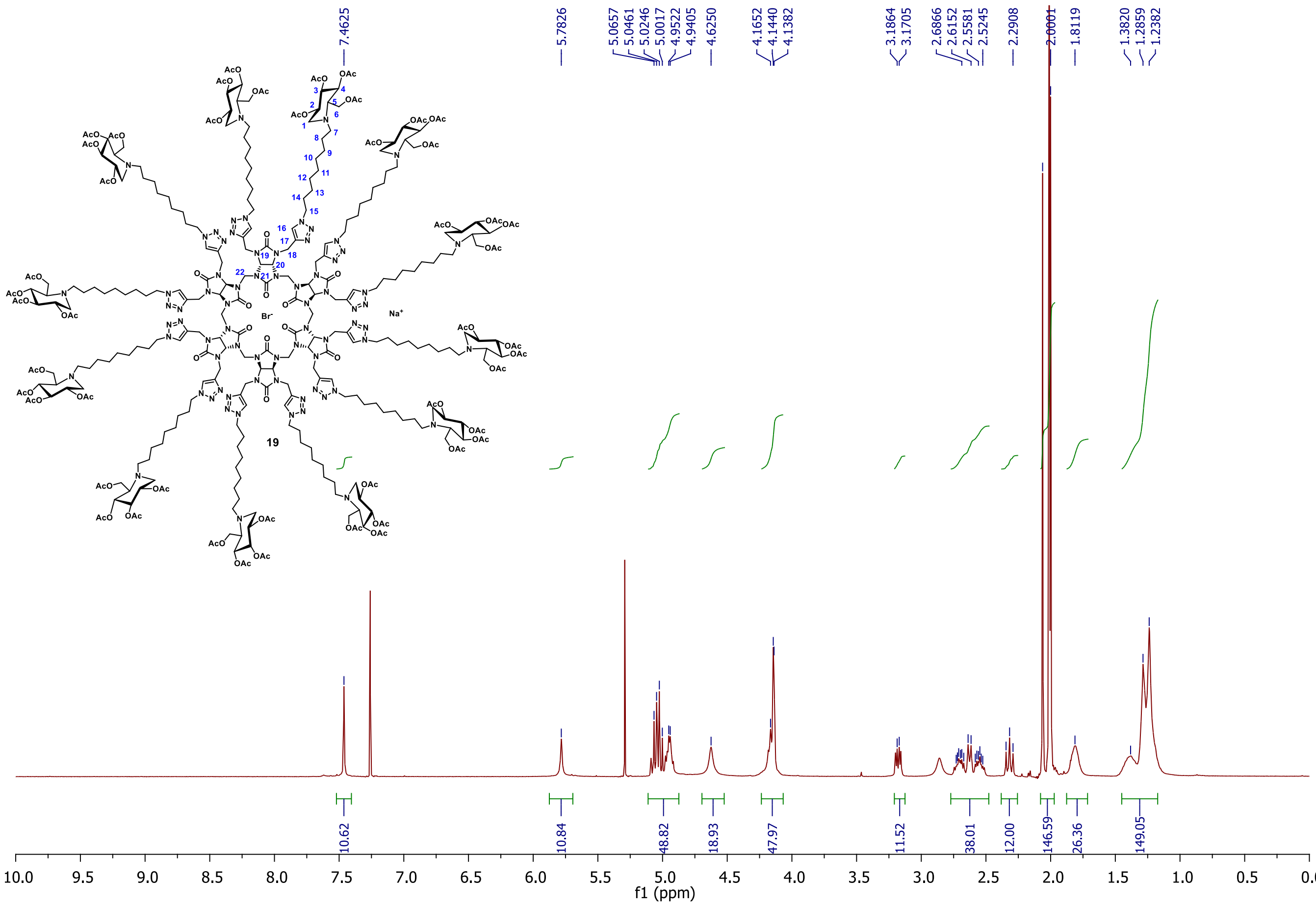

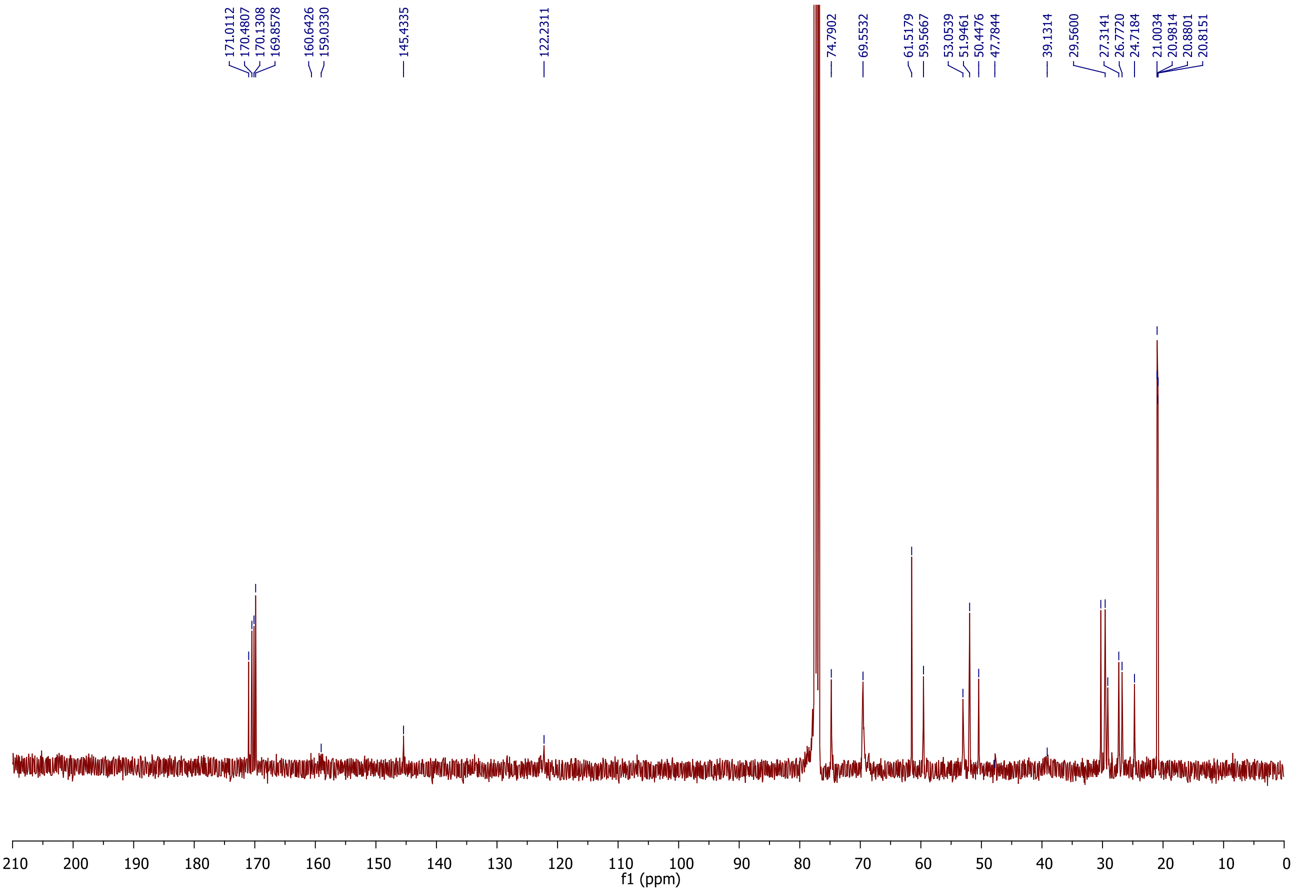

Br$^-$@(DNJ-OAc-Tripod)$_6$BU[6].Na$^+$ **20**[a)]

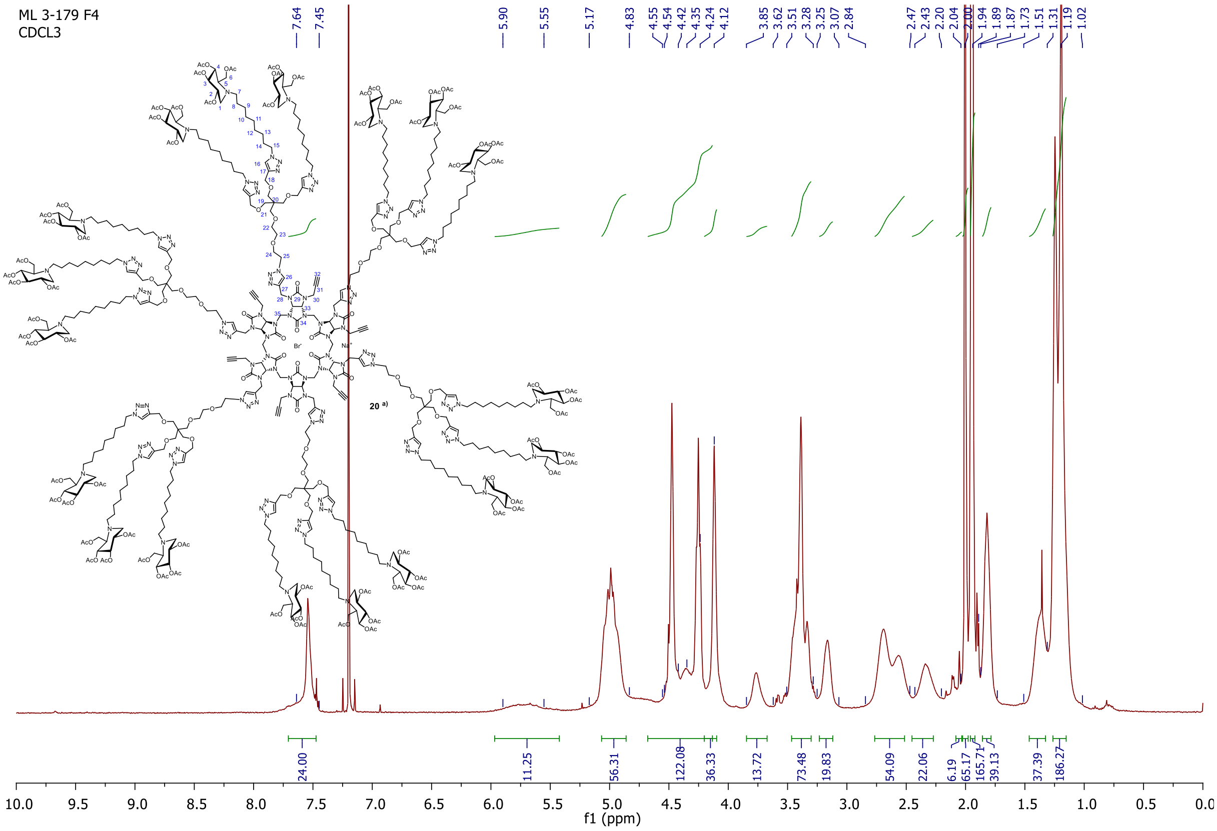

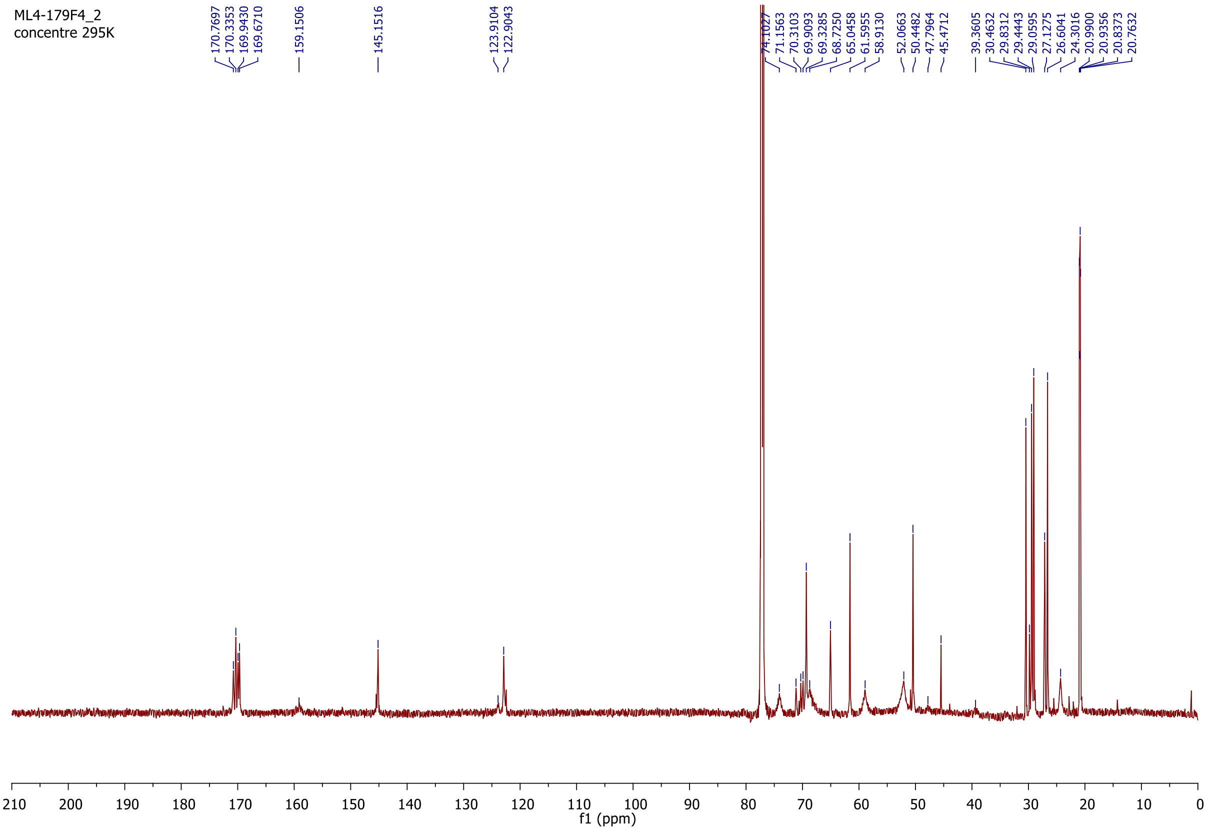

a) Structure of **20** is an "hypothetical representation"; physico chemical analyses only allowed to identify the grafting of six units of tripod **13** and the presence of six remaining alkyne functions (used of Mass Spectra analyses).

(DNJ-C6)$_8$BU[4] **3**

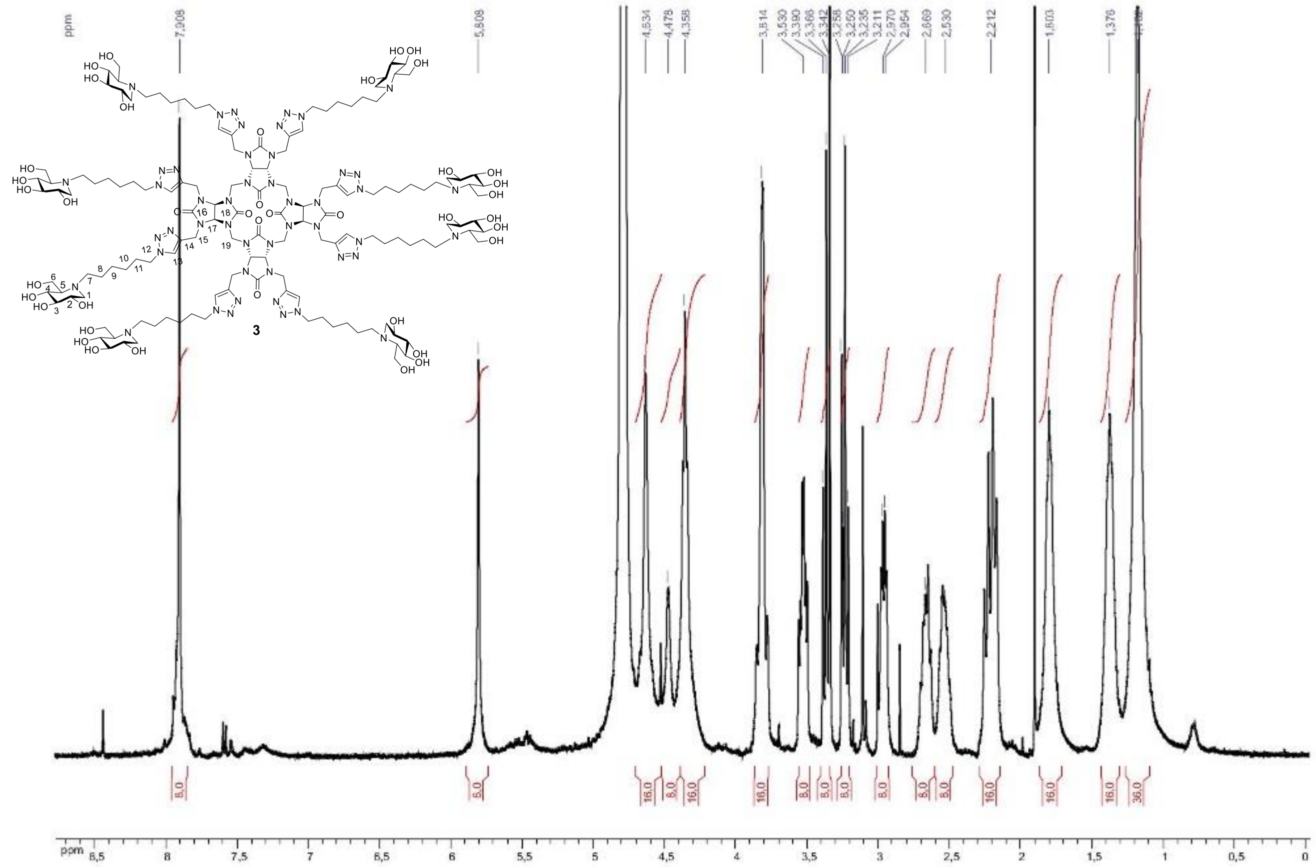

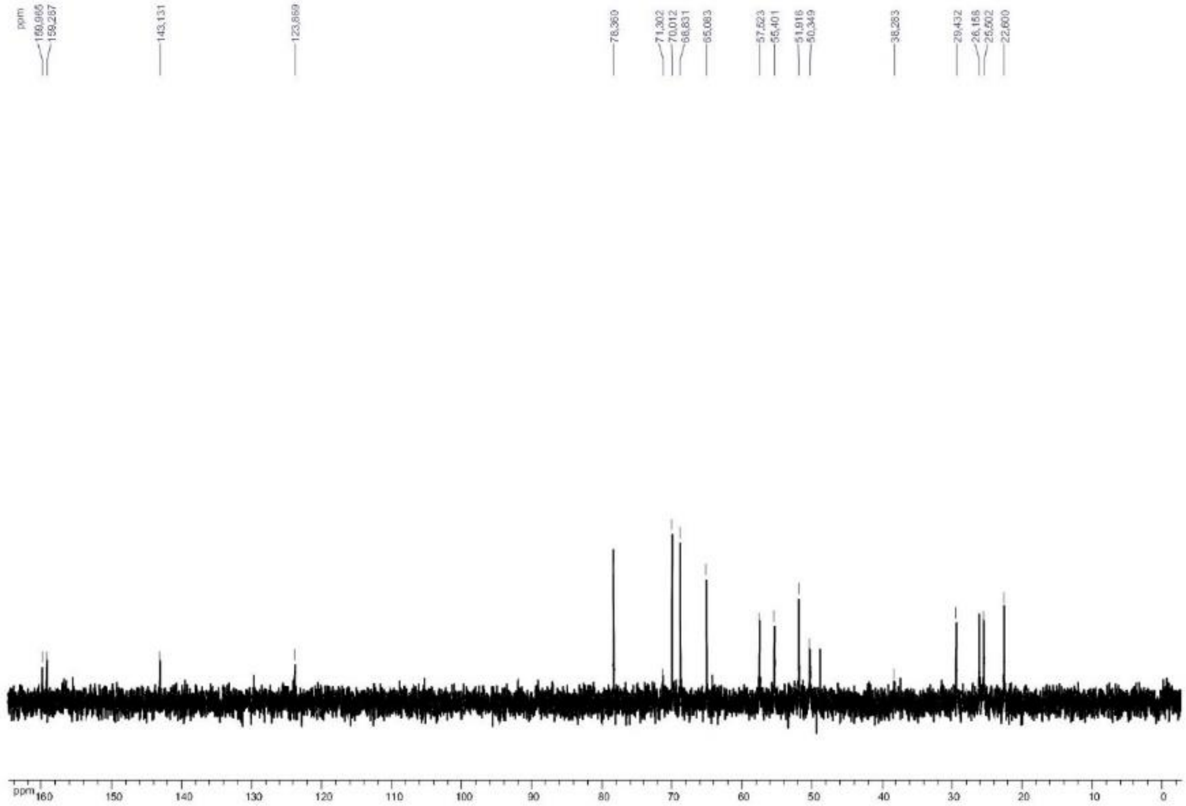

(DNJ-C9)$_8$BU[4] **4**

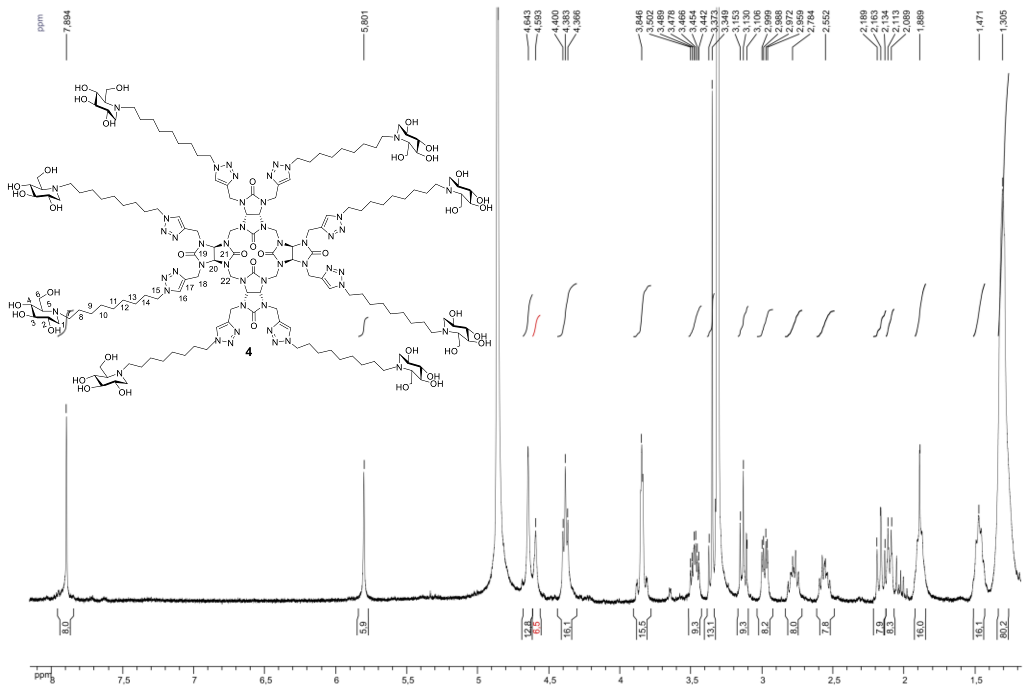
ppm
7,894
5,801
4,643
4,593
4,400
4,383
4,366
3,846
3,502
3,489
3,478
3,466
3,454
3,442
3,373
3,349
3,153
3,130
3,106
2,999
2,988
2,972
2,959
2,784
2,552
2,189
2,163
2,134
2,113
2,089
1,889
1,471
1,305
8,0
5,9
12,8
6,5
16,1
15,5
9,3
13,1
9,3
8,2
8,0
7,8
7,9
8,3
16,0
16,1
80,2
ppm 8 7,5 7 6,5 6 5,5 5 4,5 4 3,5 3 2,5 2 1,5
4

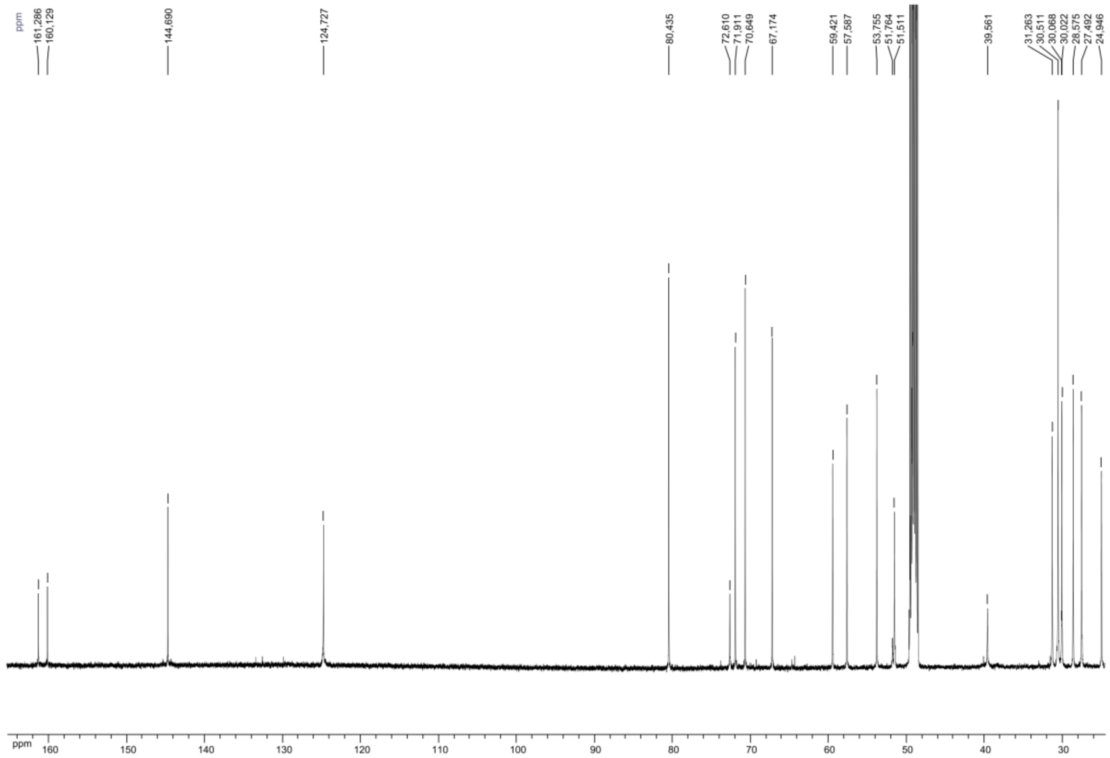
ppm
161,286
160,129
144,690
124,727
80,435
72,610
71,911
70,649
67,174
59,421
57,587
53,755
51,764
51,511
39,561
31,263
30,511
30,068
30,022
28,575
27,492
24,946
ppm 160 150 140 130 120 110 100 90 80 70 60 50 40 30

(DNJ-Tripod)$_8$BU[4] **5**

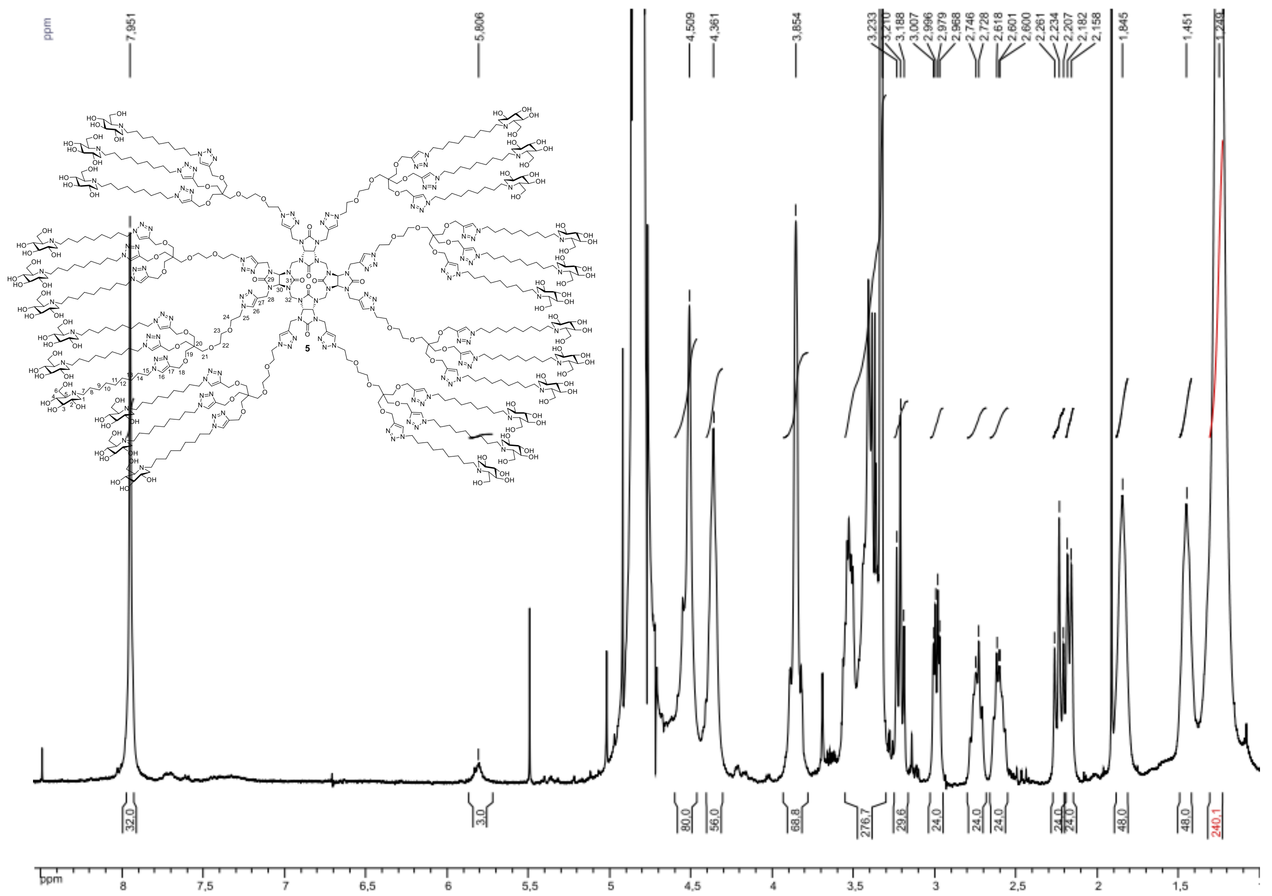

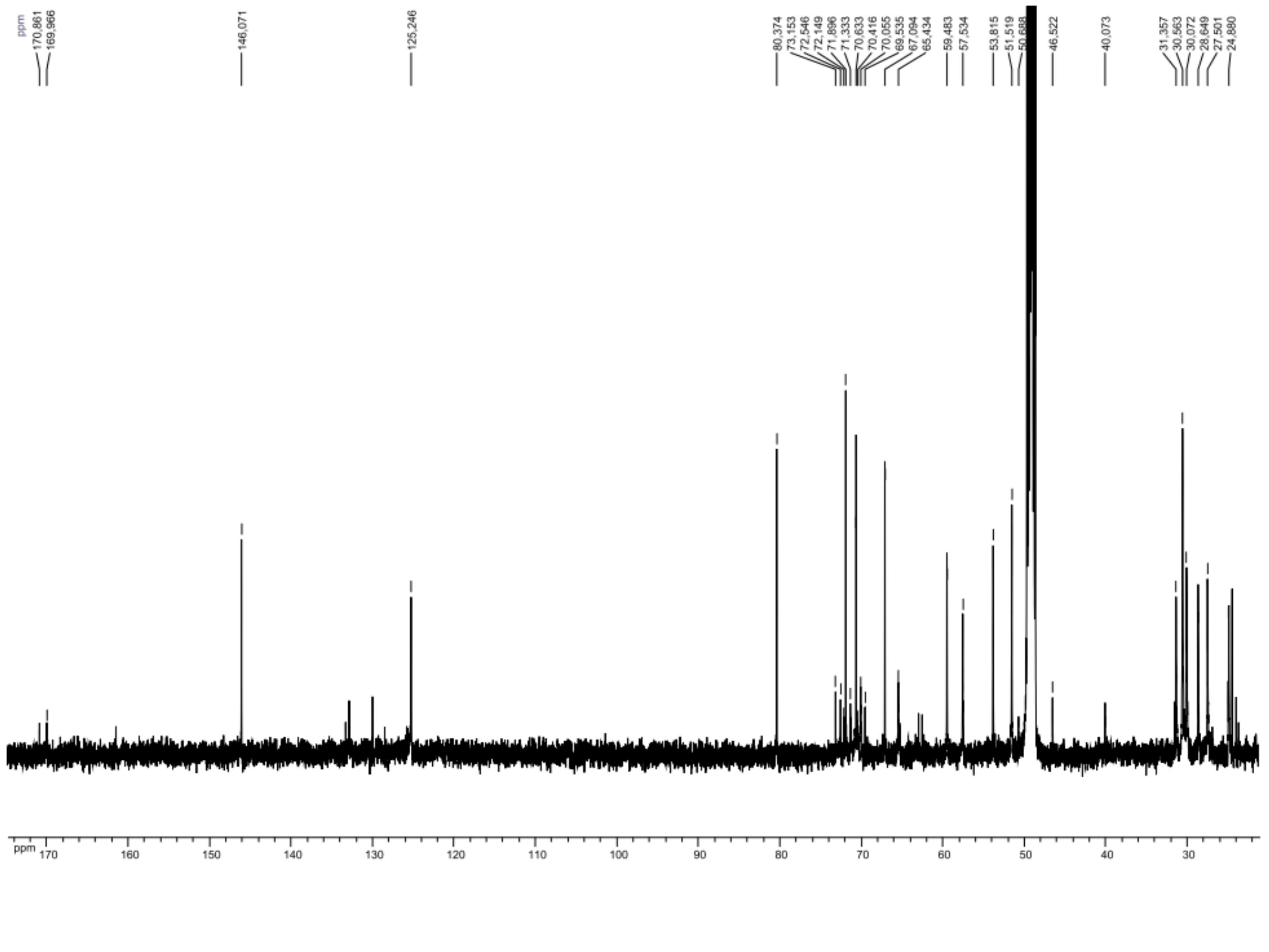

(DNJ-C6)$_{12}$BU[6] **6**

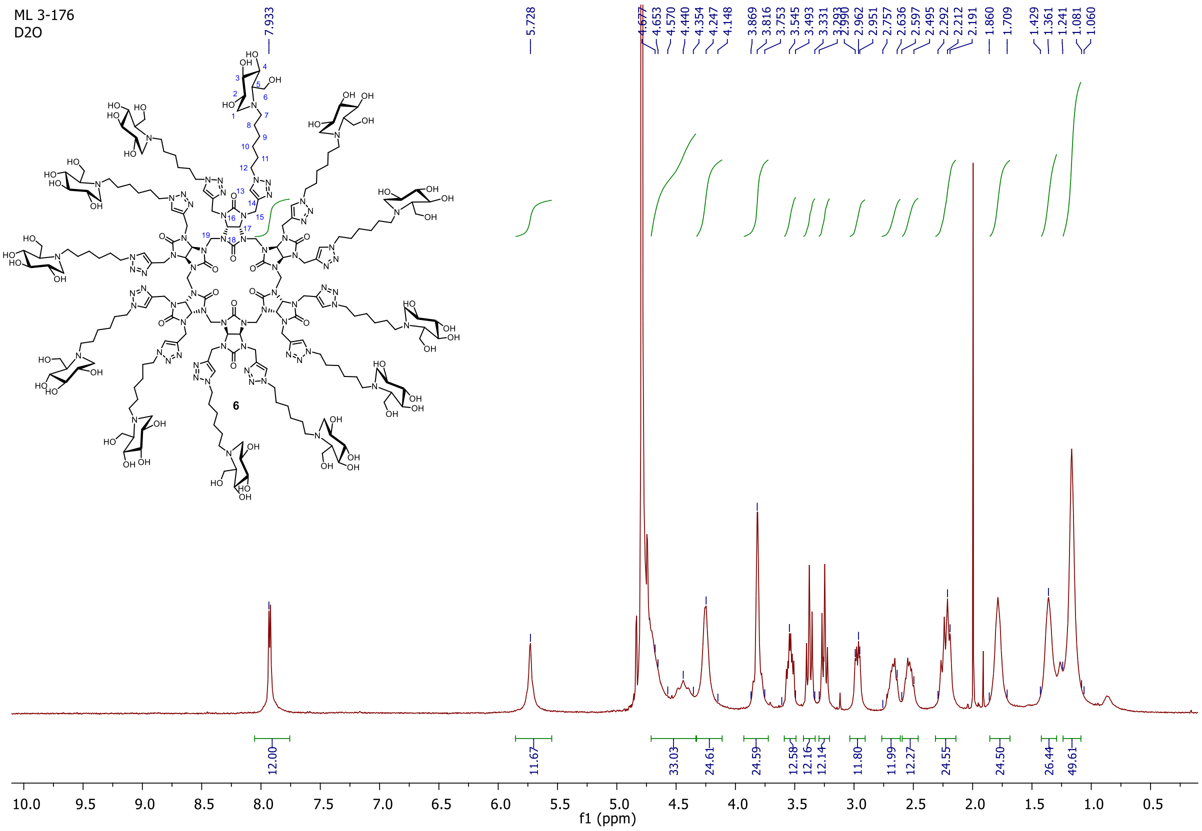

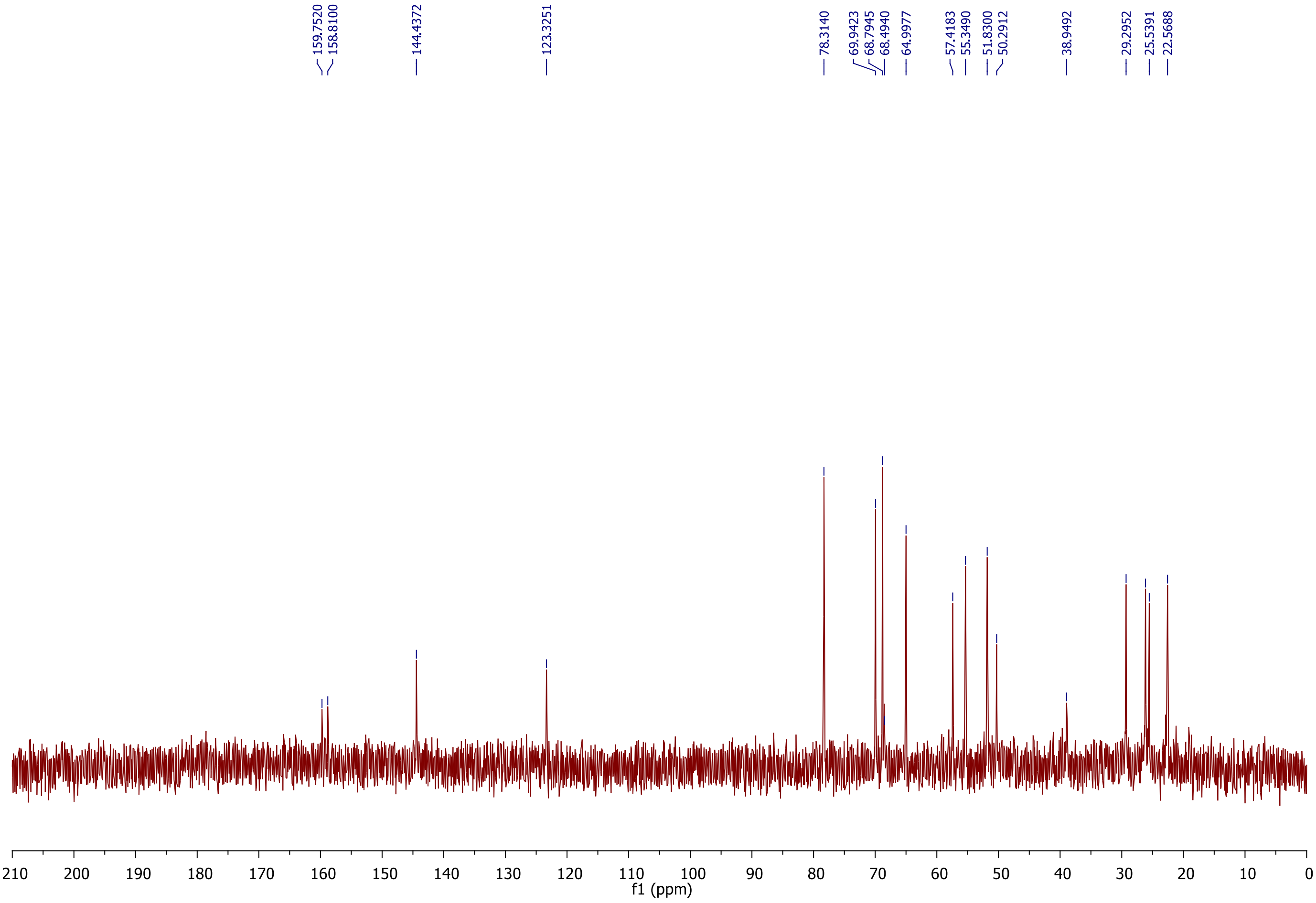

(DNJ-C9)$_{12}$BU[6] **7**

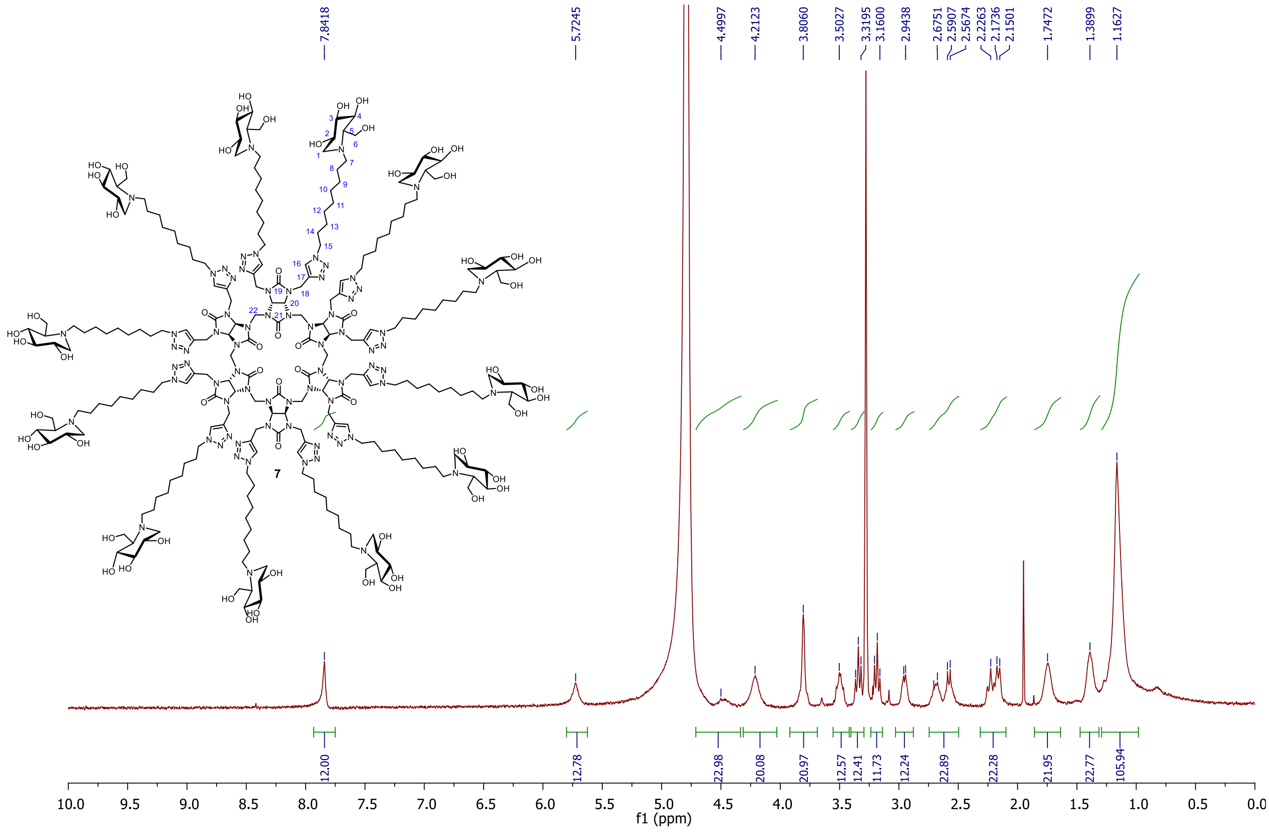

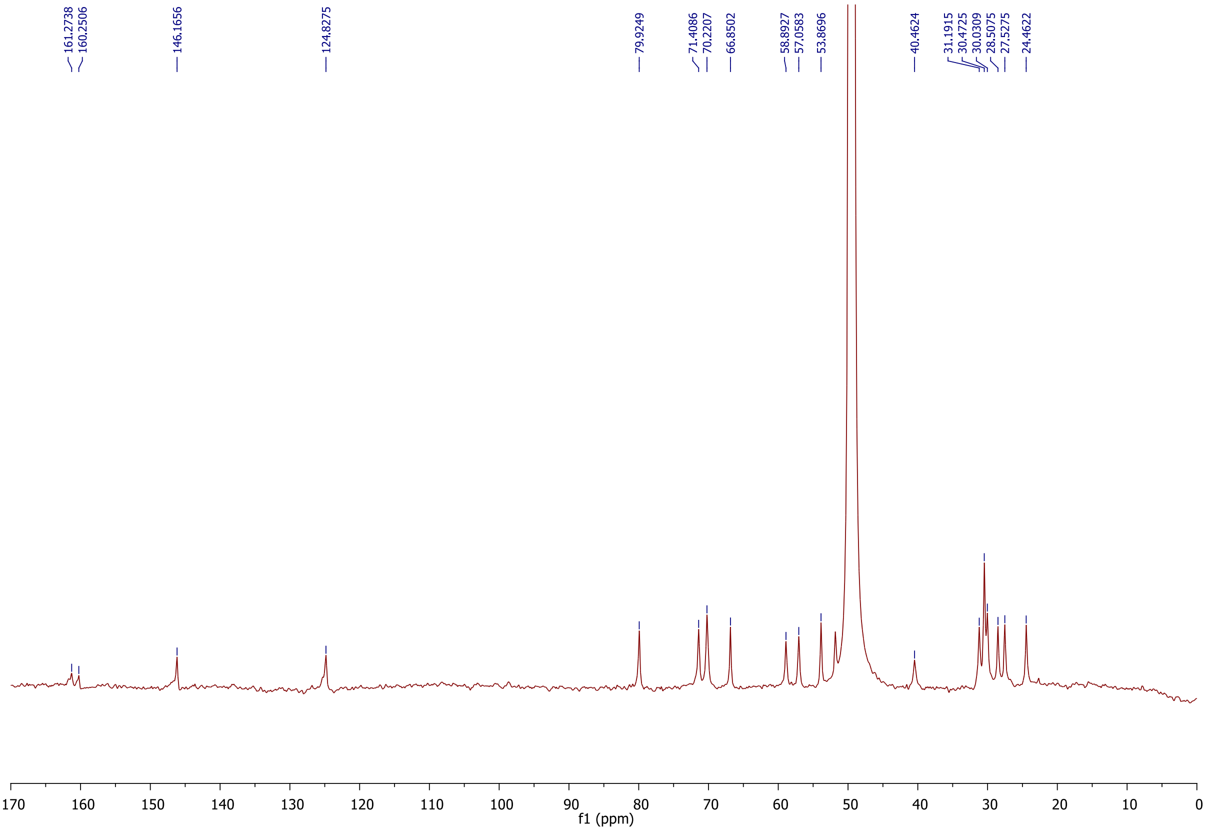

Br$^-$@(DNJ-C6)$_{12}$BU[6].TBA$^+$ **8**

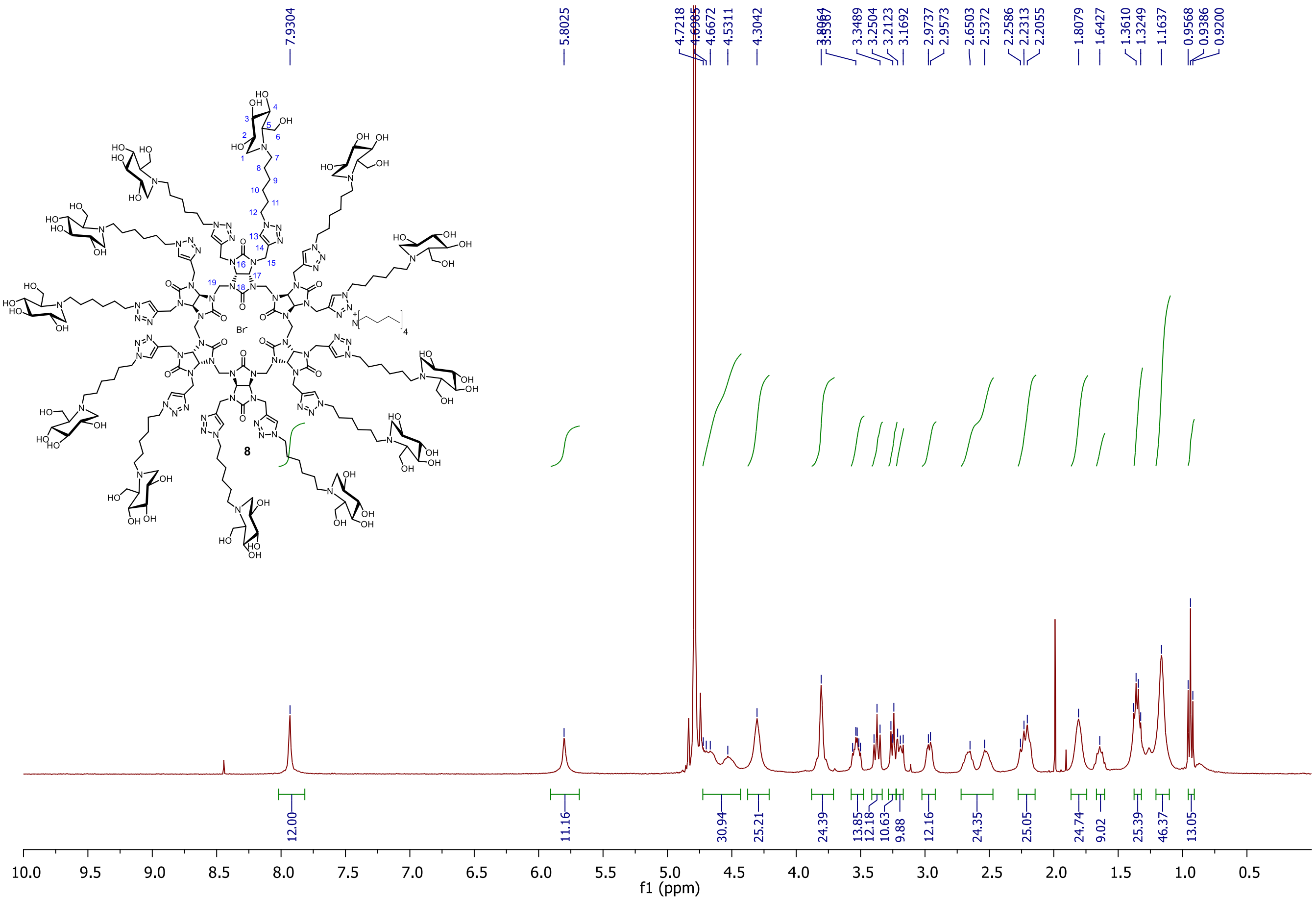

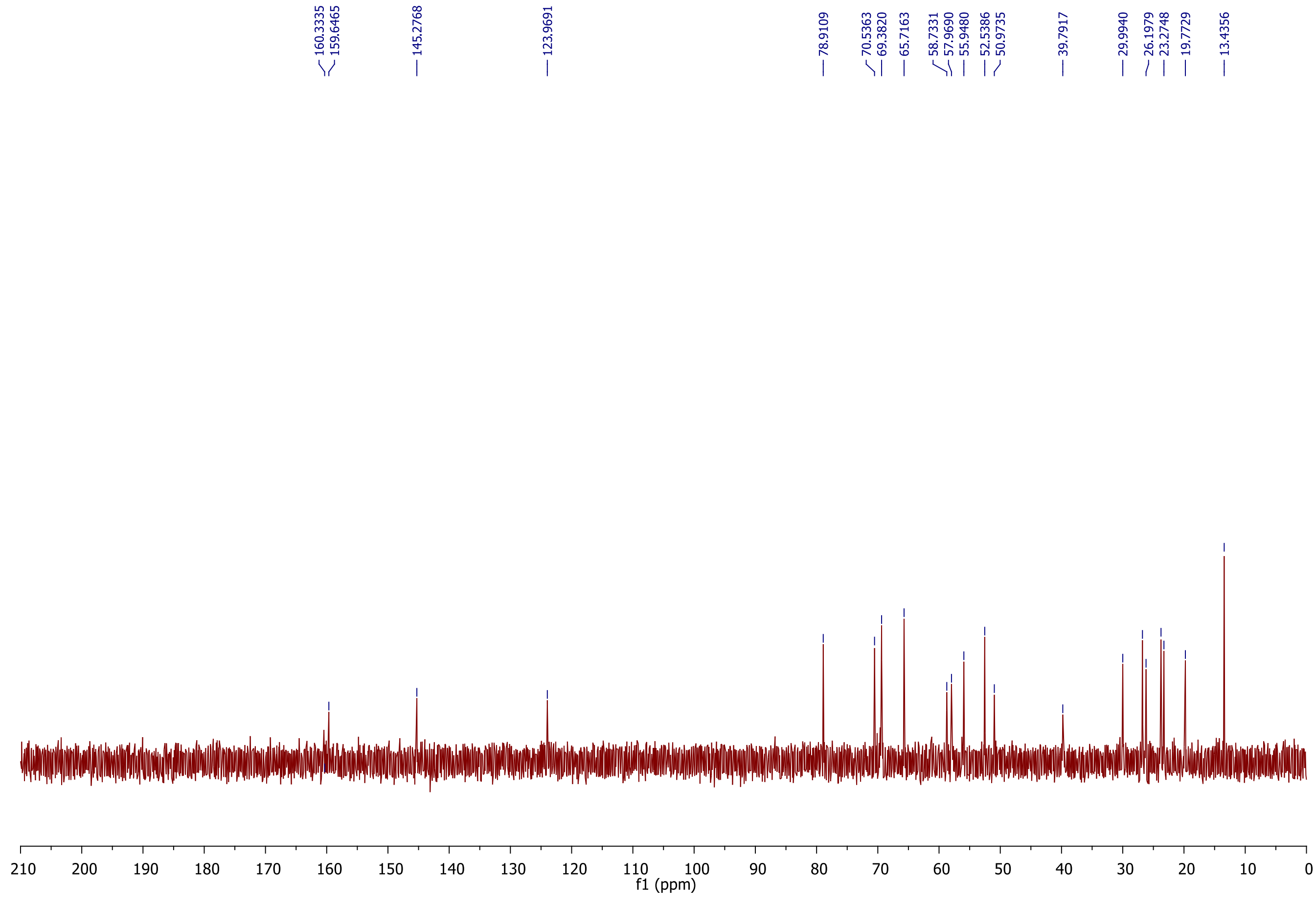

I-@(DNJ-C6)$_{12}$BU[6].TBA^{+} **9**

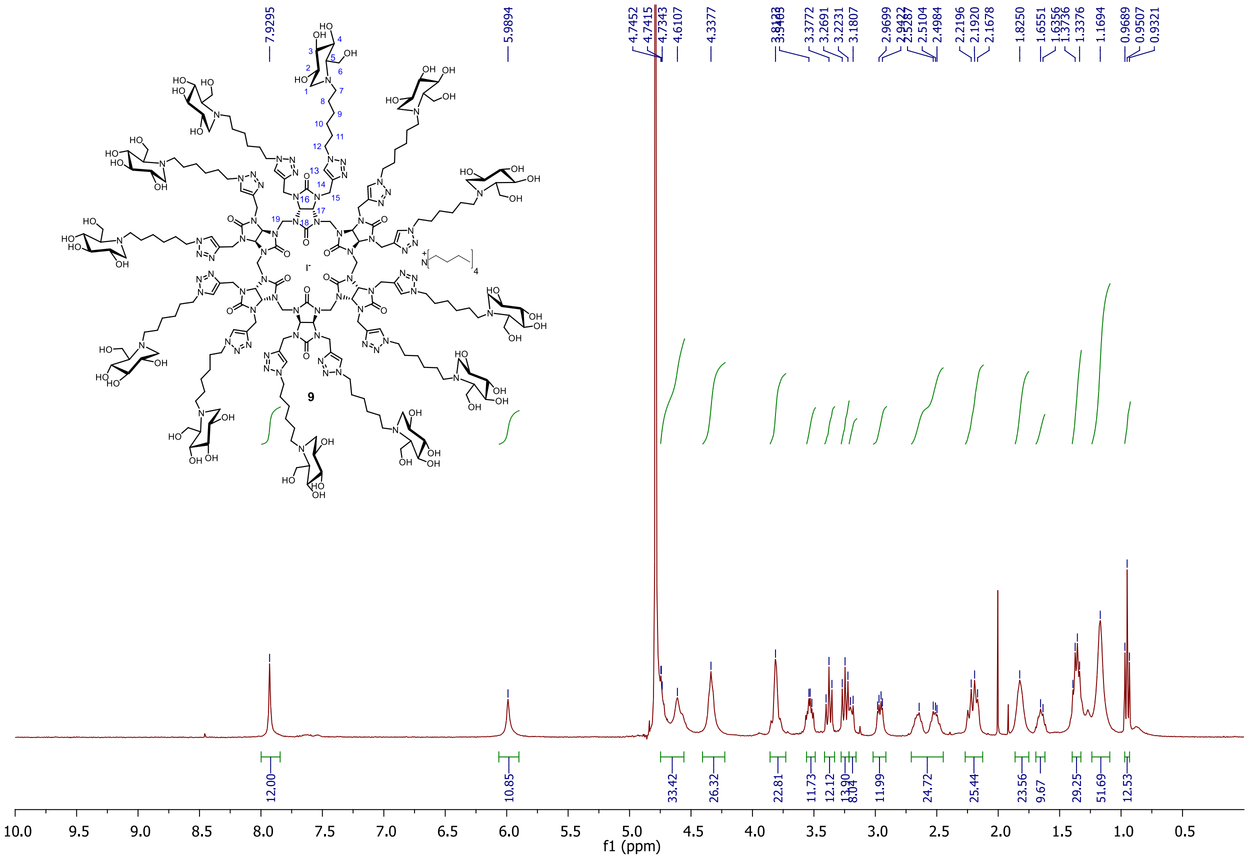

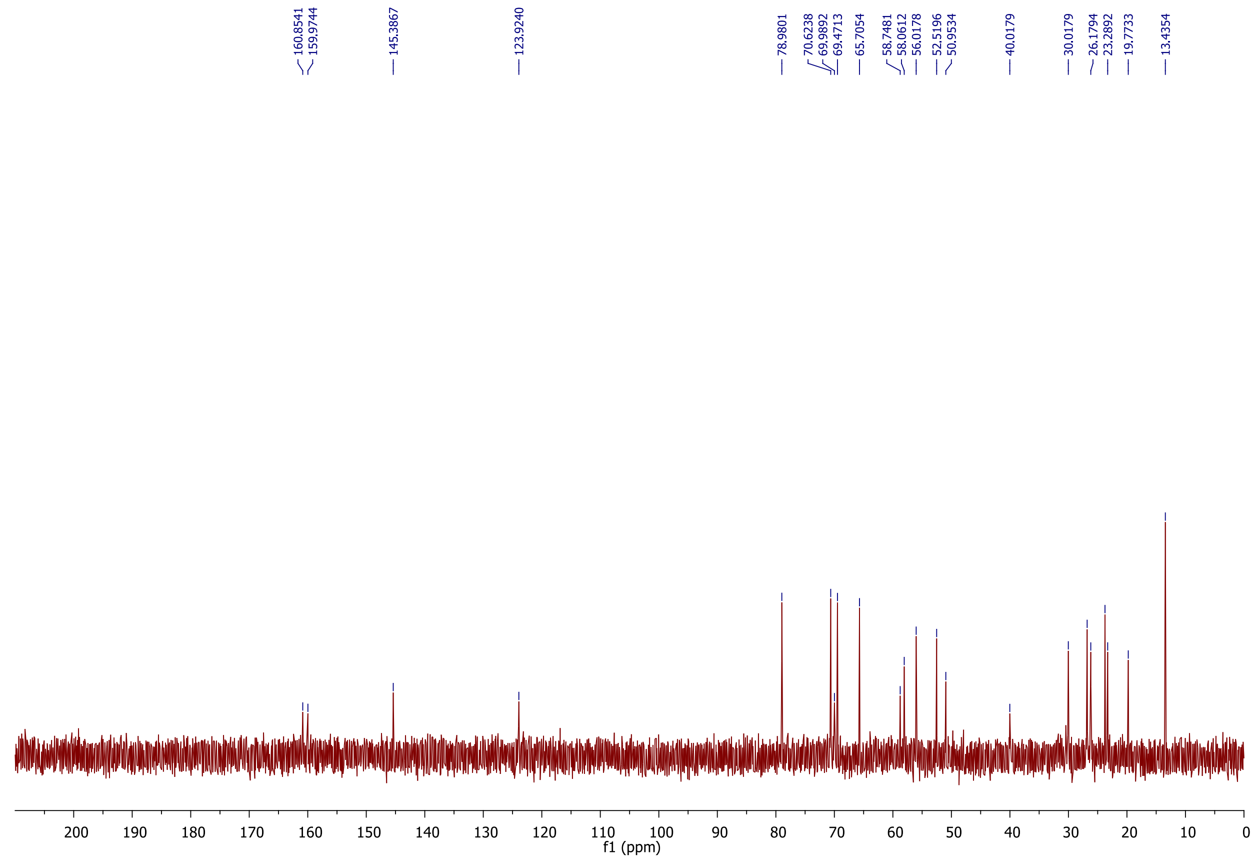

Br$^-$@(DNJ-C9)$_{12}$BU[6].TBA$^+$ **10**

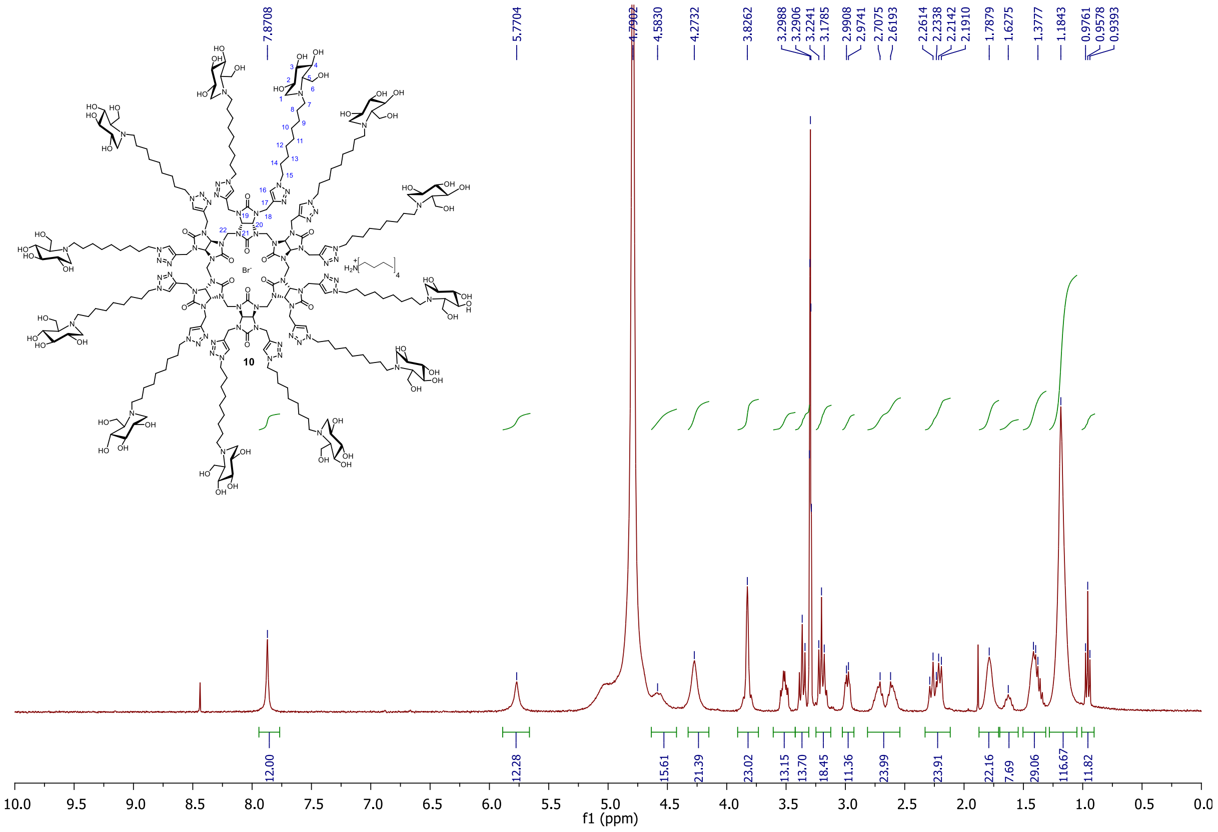

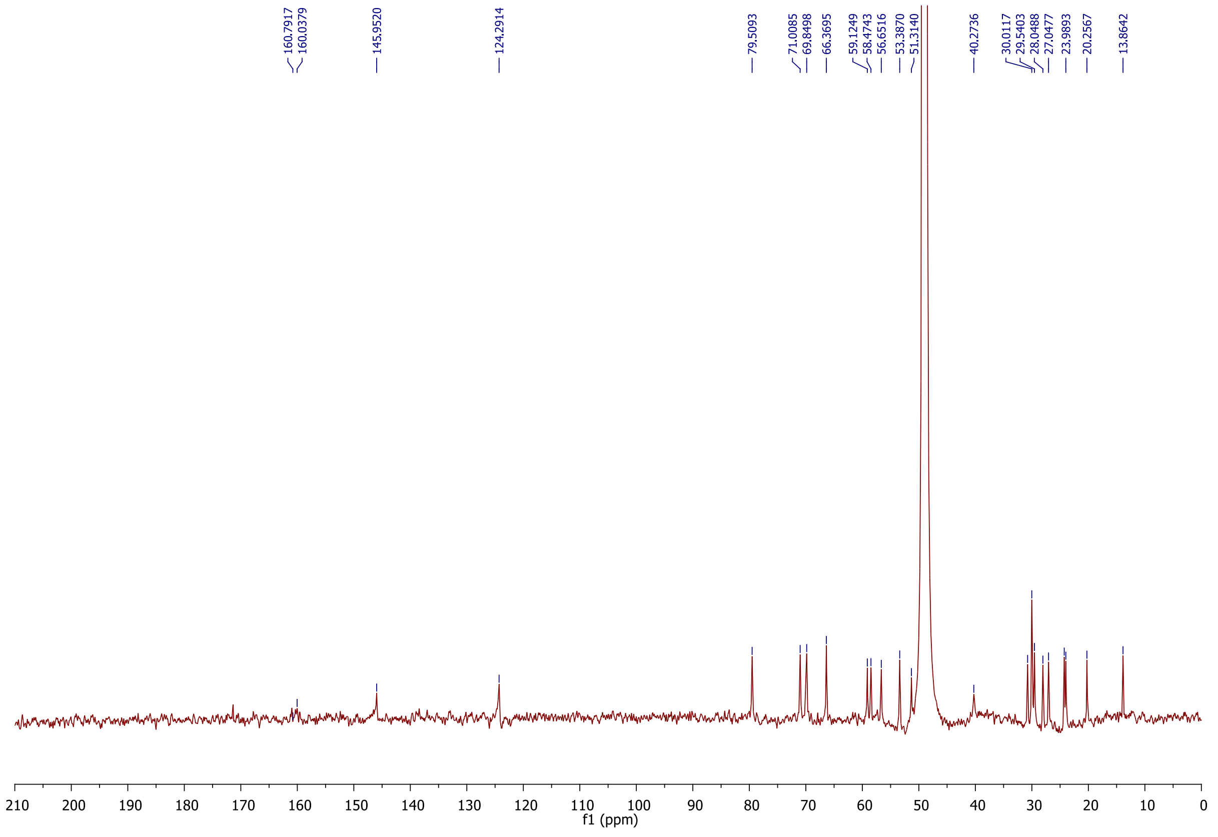

Br$^-$@(DNJ-Tripod)$_6$BU[6].Na$^+$ **11**[a)]

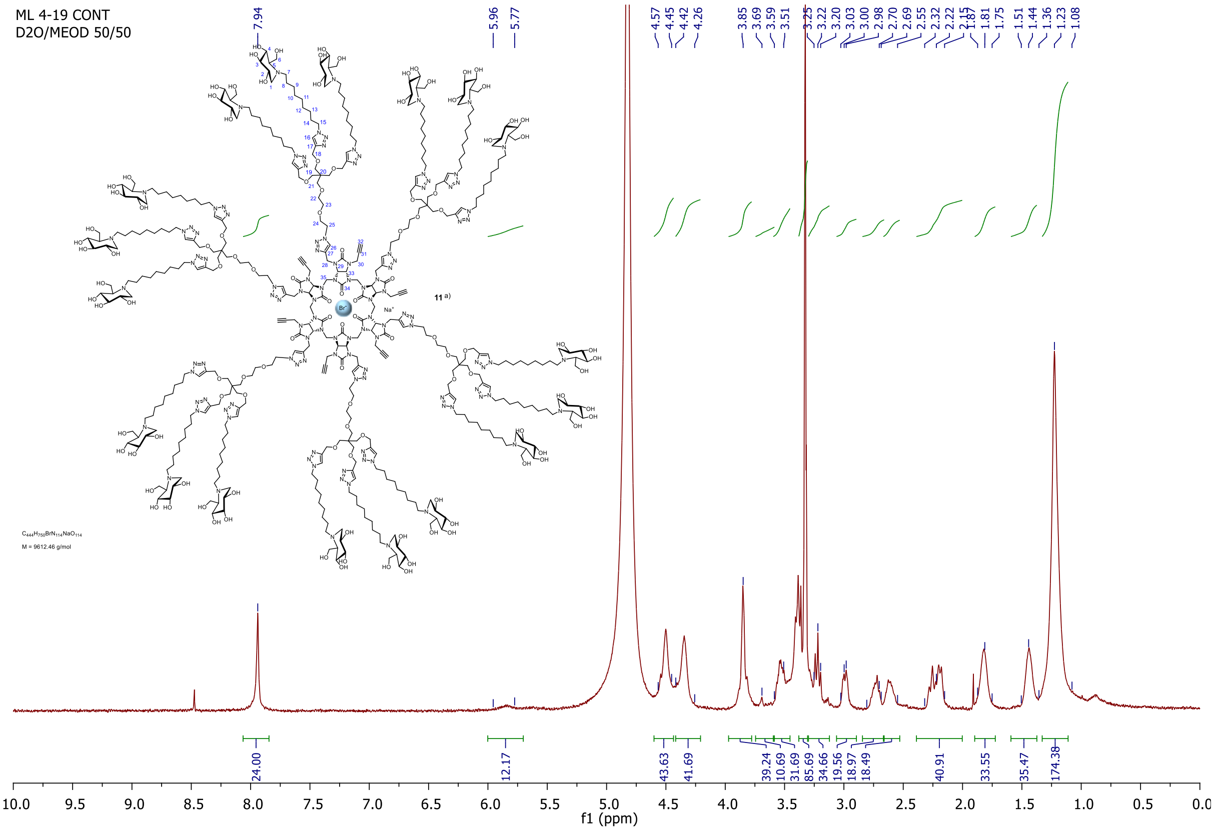

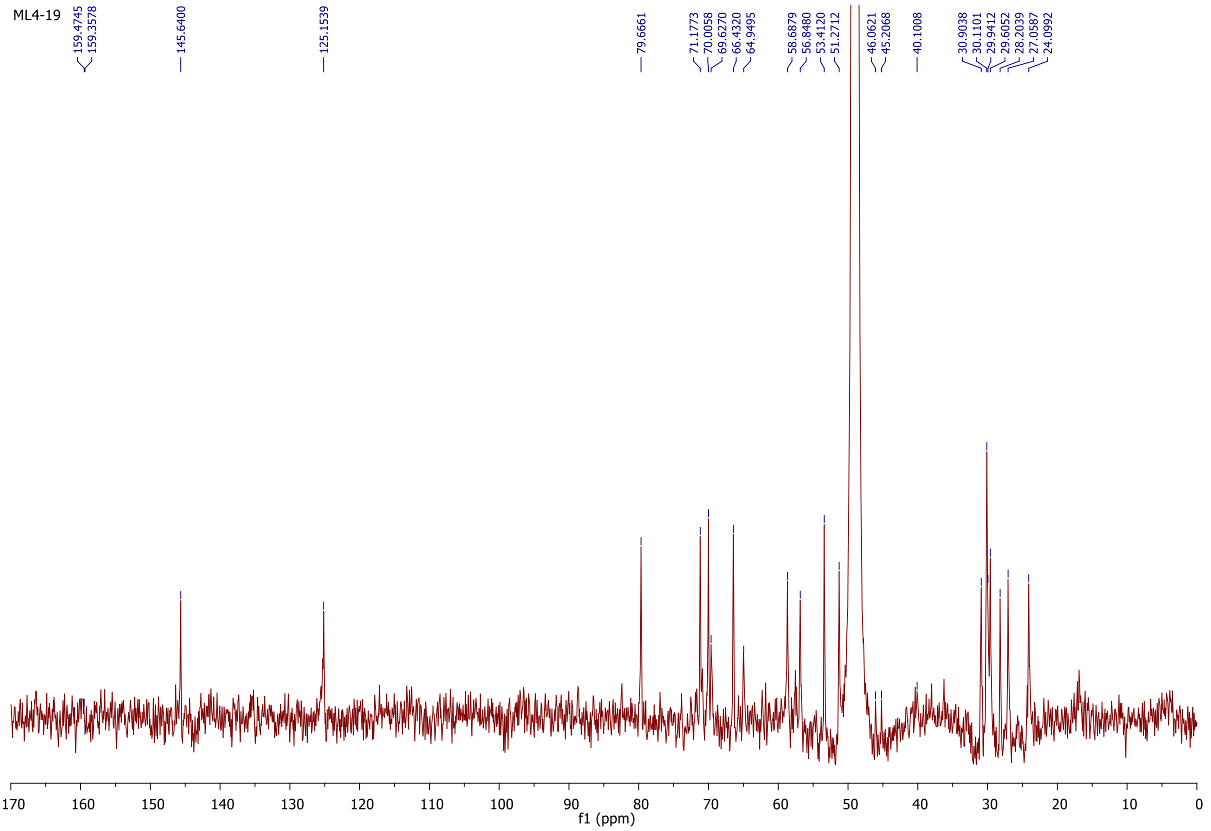

[a)]structure of **11** is an "hypothetical representation", physico chemical analyses only allowed to identify the grafting of six units of tripod **13** and the presence of six remaining alkyne functions (used of Mass Spectra analyses).

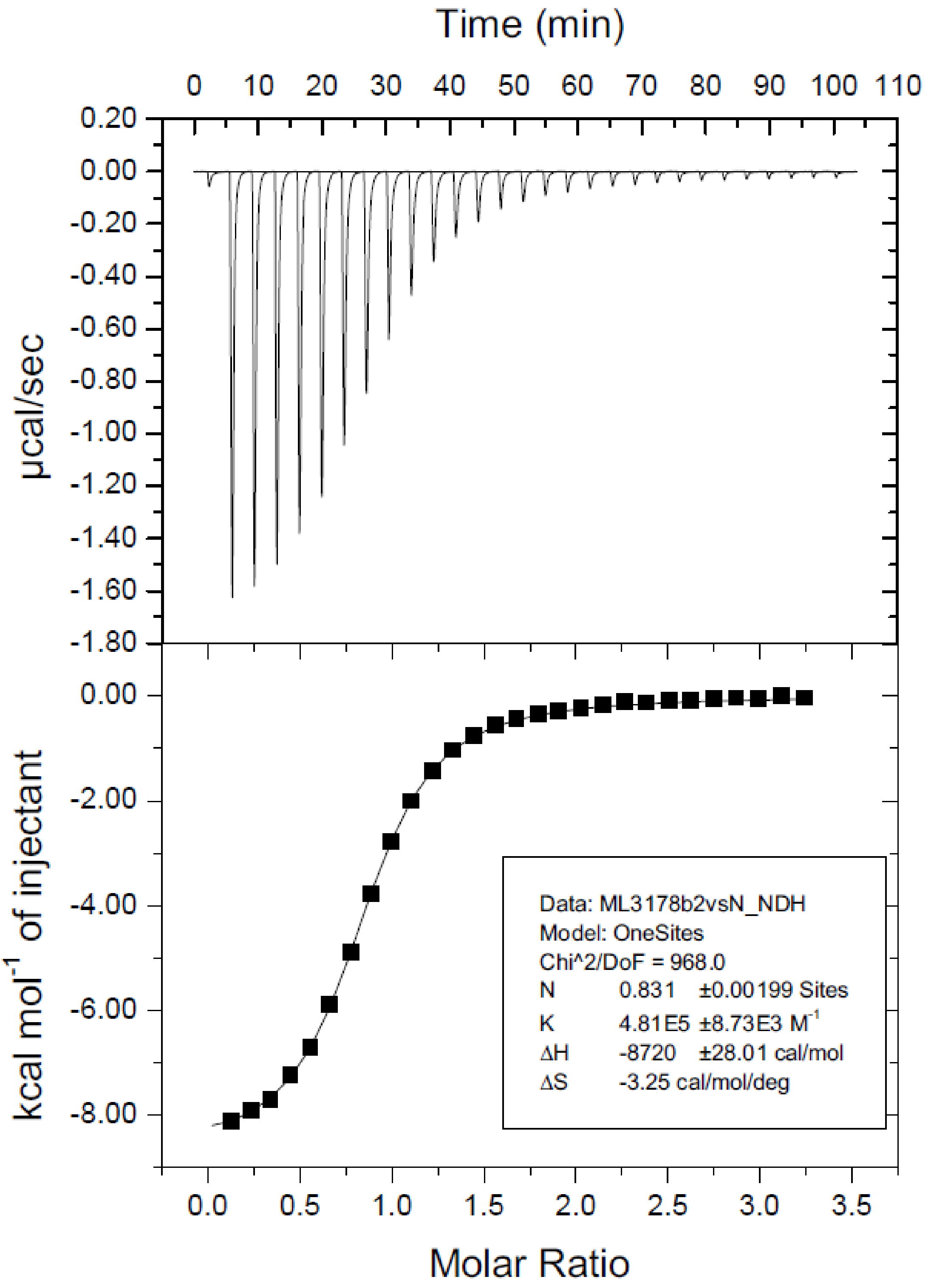

Figure S1. Isothermal titration calorimetry of I^- binding to $(DNJ\text{-}C6)_{12}BU[6]$ **6** in H_2O.

Top: Data were obtained from the sequential injections of 10µL of guest solution (0.6 mM NaI) to **BU 6** (0.05 mM). Bottom: Plot of the total heat released as a function of the total ligand concentration for the titration shown upper.

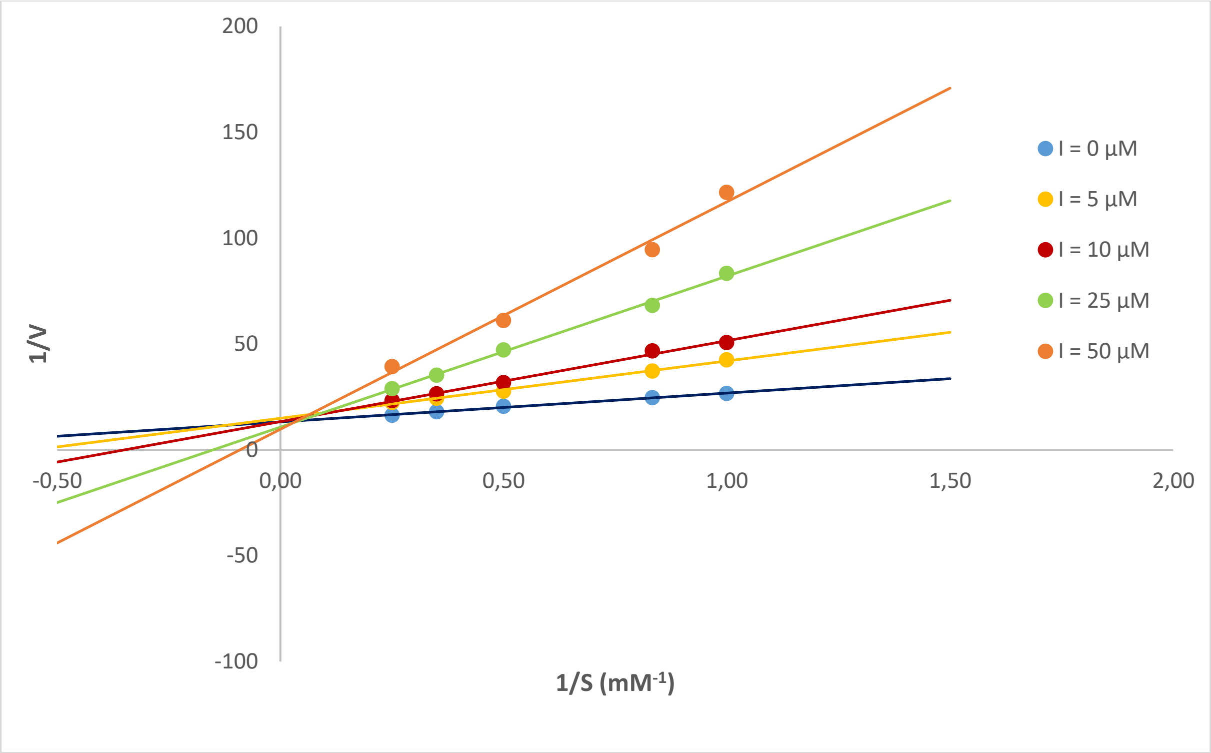

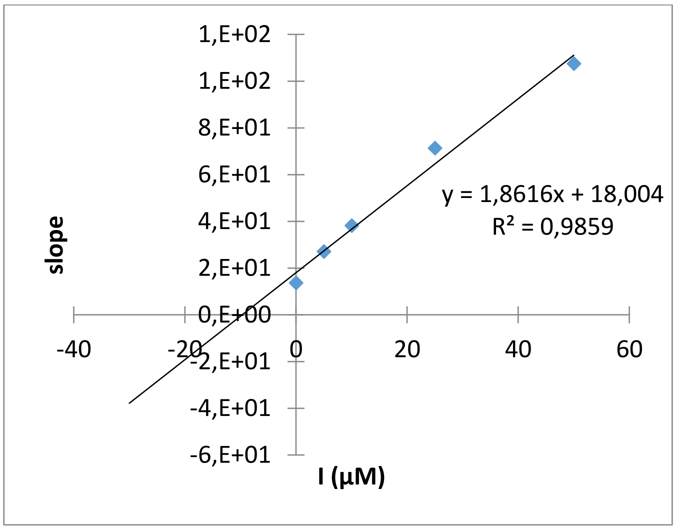

Figure S2 . Lineweaver-Burk plot of compound (DNJ-C6)$_8$BU[4] **3** against JB α-man and the corresponding replot showing a competitive inhibition *(K_I* = 9.7 +/- 2.9 µM)

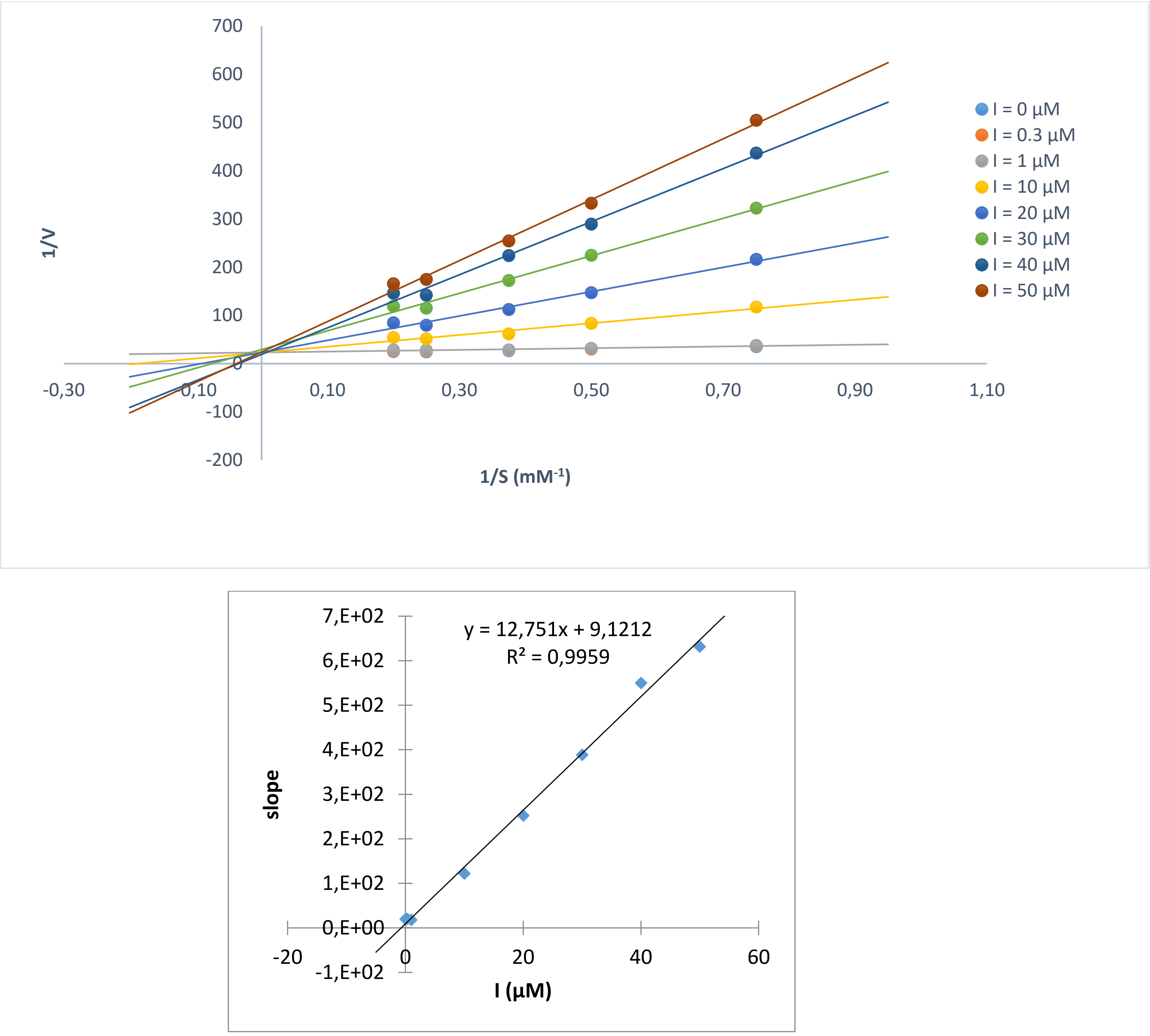

Figure S3 . Lineweaver-Burk plot of compound $(DNJ\text{-}C9)_8BU[4]$ **4** against JB α-man and the corresponding replot showing a competitive inhibition (K_I = 0.71 +/- 0.55 µM).

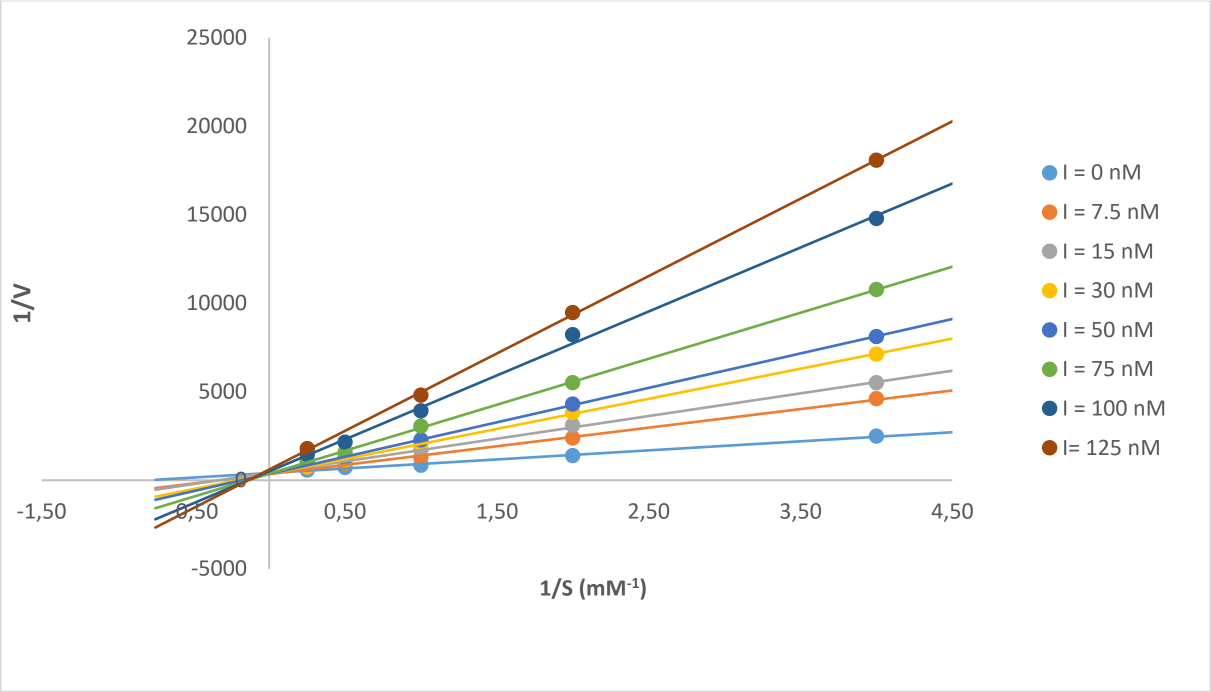

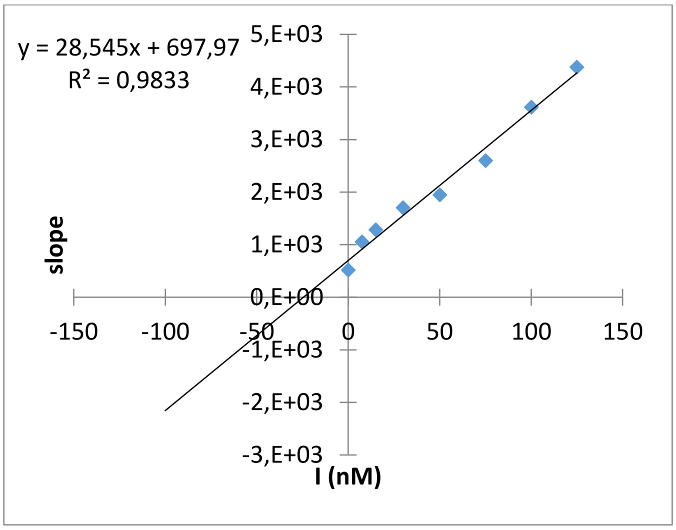

Figure S4. Lineweaver-Burk plot of compound (DNJ-Tripod)8BU[4] 5 against JB α-man and the corresponding replot showing a competitive inhibition *(K_I = 24 +/- 4 nM)*.

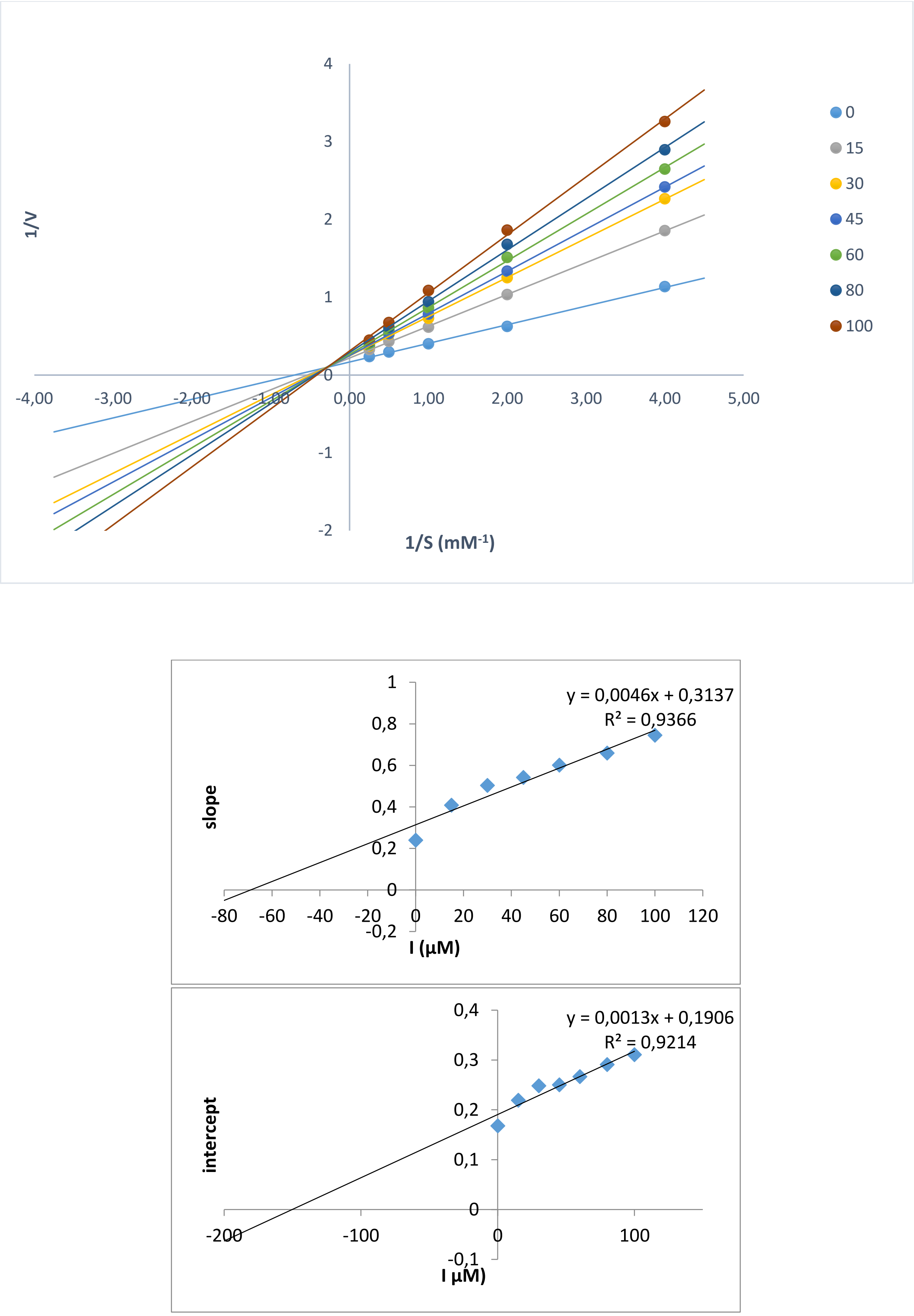

Figure S5. Lineweaver-Burk plot of compound (DNJ-C6)12BU[6] **6** against JB α-man and the corresponding replots showing a mixed inhibition (K_i = 68.2 +/- 10.3 µM, K_i' = 147 +/-21 µM).

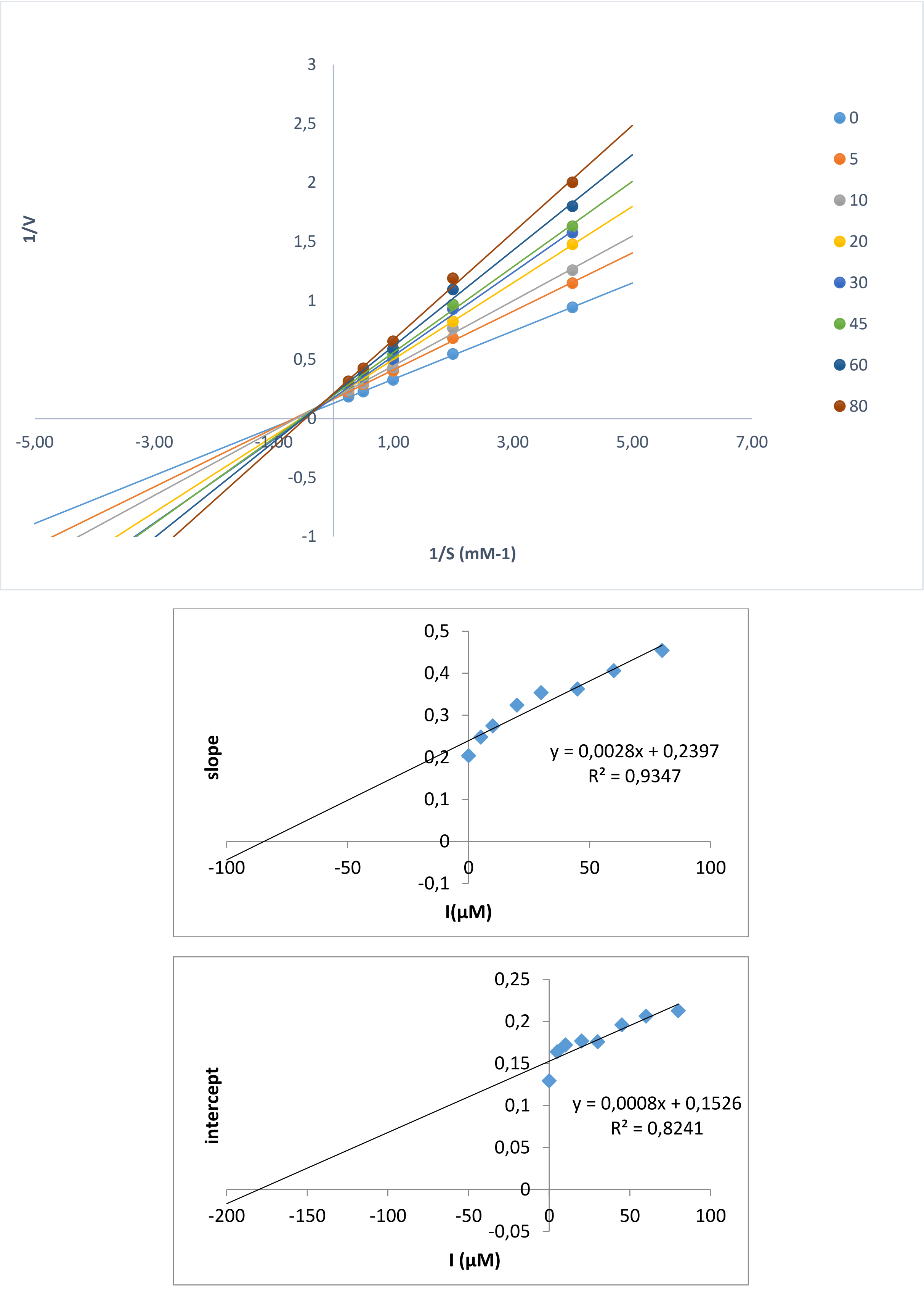

Figure S6. Lineweaver-Burk plot of compound $Br^{-}@(DNJ\text{-}C6)_{12}BU[6].TBA^{+}$ 8 against JB α-man and the corresponding replots showing a mixed inhibition (K_I = 85.6 +/- 10.3 µM, K_I' = 191 +/-37 µM).

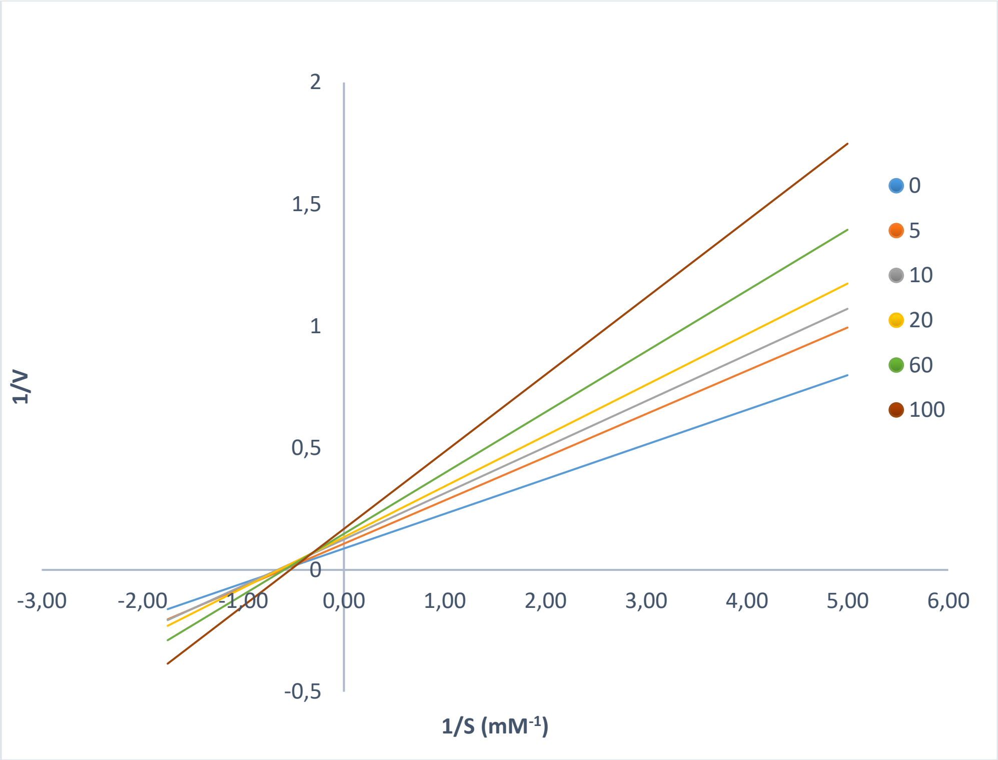

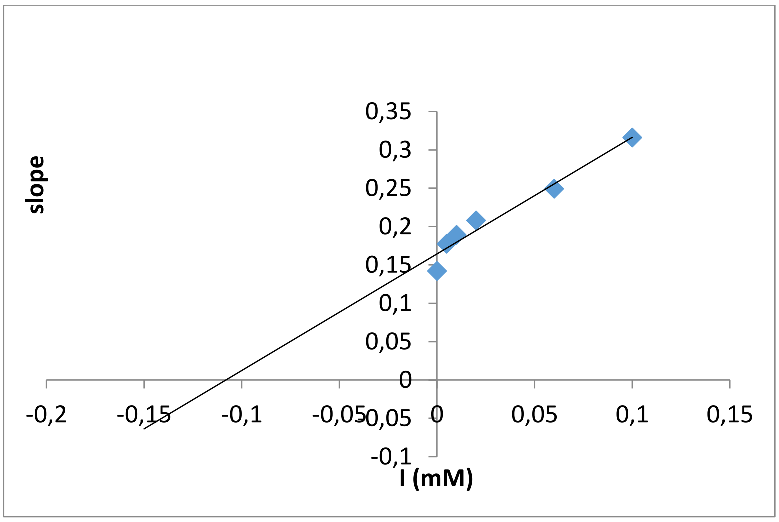

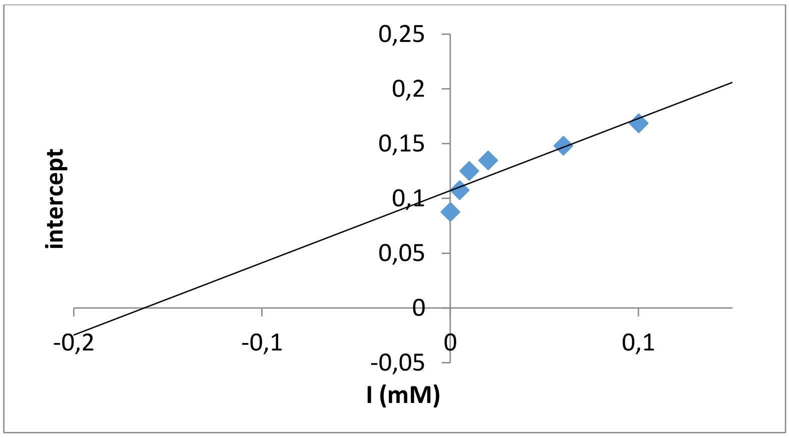

Figure S7. Lineweaver-Burk plot of compound I^-@(DNJ-C6)$_{12}$BU[6].TBA$^+$ **9** against JB α-man and the corresponding replots showing a mixed inhibition (K_i = 108 +/- 13 µM, K_i' = 162 +/-40 µM).

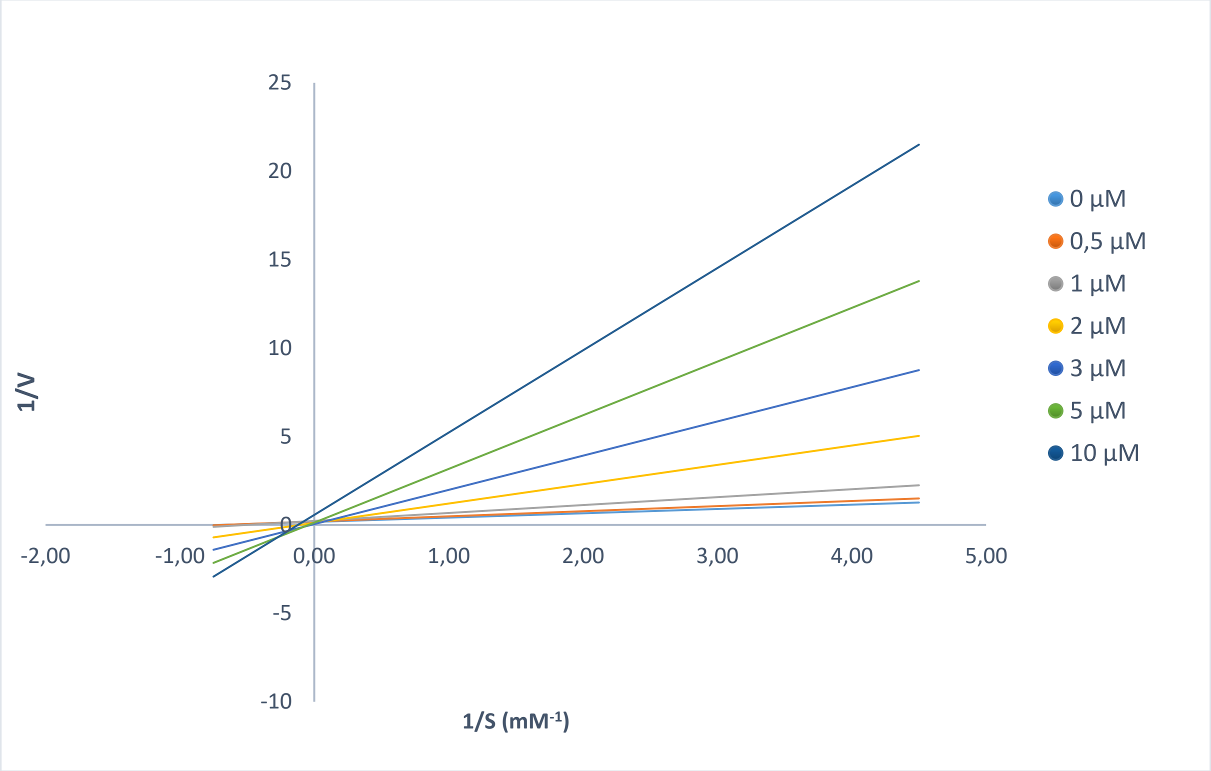

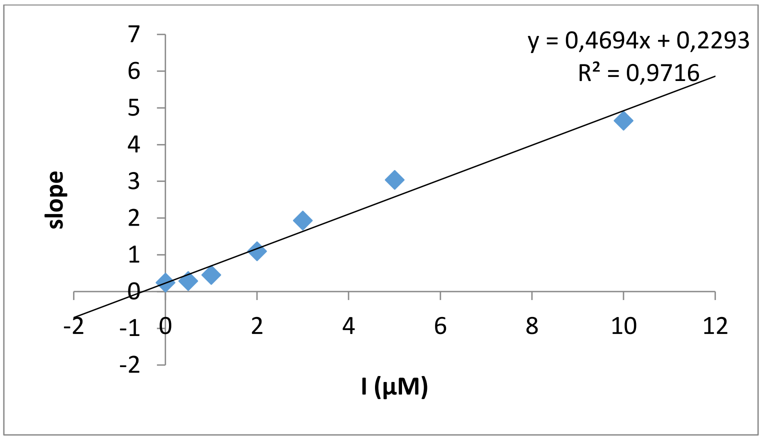

Figure S8. Lineweaver-Burk plot of compound Br^{-}@(DNJ-C9)$_{12}$BU[6].TBA^{+} **10** against JB α-man and the corresponding replot showing a competitive inhibition (K_I = 0.488 +/- 0.343 µM).

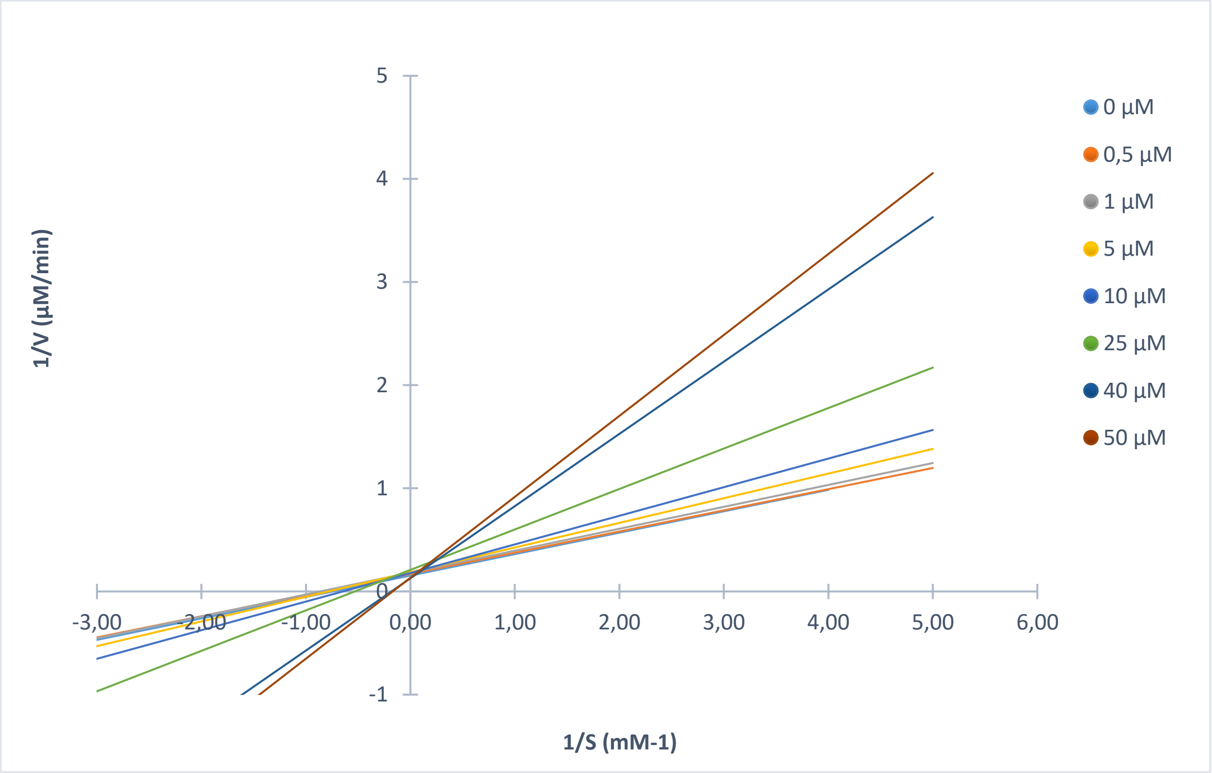

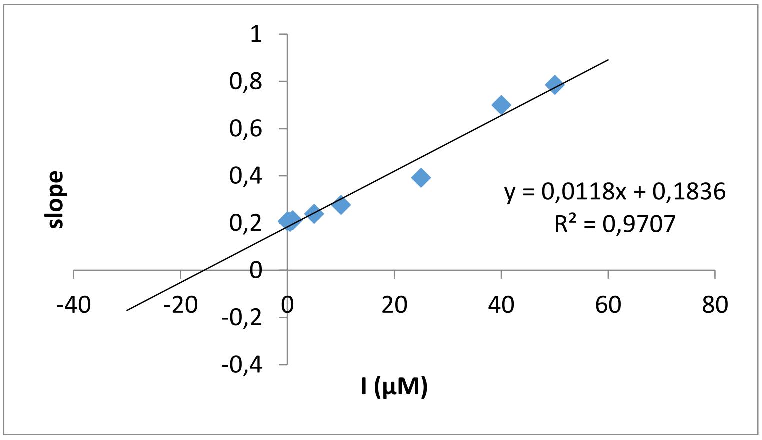

Figure S9. Lineweaver-Burk plot of compound Br^{-}@(DNJ-Tripod)$_6$BU[6].Na^{+} **11** against JB α-man and the corresponding replot showing a competitive inhibition (K_I = 15.6 +/- 2.1 nM)
